# Timing Is Everything: The Effect of Exposure to Pollution on Wildlife Gut Microbiota Is Contingent on Season

**DOI:** 10.1111/mec.70346

**Published:** 2026-04-22

**Authors:** Andrii Vasylenko, Eugene Tukalenko, Anton Lavrinienko, Timothy A. Mousseau, Tapio Mappes, Phillip C. Watts

**Affiliations:** ^1^ Department of Biological and Environmental Science University of Jyväskylä Jyväskylä Finland; ^2^ Department of Radiobiology and Radioecology Institute for Nuclear Research of the National Academy of Sciences of Ukraine Kyiv Ukraine; ^3^ Department of Health Sciences and Technology ETH Zurich Zürich Switzerland; ^4^ Department of Biological Sciences University of South Carolina Columbia South Carolina USA

**Keywords:** Anna Karenina principle, bacteria, gut microbiota, pollution, radionuclide, seasonal change

## Abstract

Exposure to environmental pollutants can disrupt the gut microbiota, but how pollutants impact natural, seasonal changes in wildlife gut microbiota remains unknown. We quantified how exposure to radionuclides affected temporal changes in the gut microbiota of bank voles ( *Clethrionomys glareolus* ) inhabiting the Chornobyl Exclusion Zone (CEZ), Ukraine. Wild‐caught bank voles from contaminated and uncontaminated areas within the CEZ were released into field enclosures that differed in their levels of environmental radionuclides so that the gut microbiota could be longitudinally sampled from animals experiencing a known difference in contamination. Using 16S rRNA amplicon sequencing, we uncovered pronounced seasonal changes in alpha and beta diversity. Underlying this seasonal variation, exposure to radionuclides had a significant influence on beta diversity. Notably, the Bacillota (formerly Firmicutes) to Bacteroidota (formerly Bacteroidetes) (F:B) ratio and the number and composition of differentially abundant bacterial genera differed with time. Exposure to environmental radionuclides did not increase the dispersion in gut microbiota beta diversity. This contrasts with expectations of the Anna Karenina principle for microbiota, which predicts that exposure to a stressor leads to more stochastic, individualised changes in community composition, resulting in increased variation among individuals. Our data demonstrate how a cross‐sectional study can fail to capture the wider effects of pollutants on the gut microbiota of wild animals because of natural, seasonal dynamics in the microbial communities. Also, we highlight the importance of temporal monitoring of wildlife to uncover context‐dependent effects of pollutants on microbiota. Exposure to radionuclides is associated with changes in the gut microbiota of wild bank voles, but in a seasonally contingent manner that is likely shaped by interactions between host diet, physiology and the level of environmental stress.

## Анотація

Полютанти навколишнього середовища можуть порушувати мікробіоту кишечника, водночас наразі невідомо, як вони впливають на природні сезонні зміни мікробіоти кишечника диких тварин. Ми кількісно визначили вплив радіонуклідів на часові зміни мікробіоти кишечника рудої нориці ( *Clethrionomys glareolus* ), що мешкала у зоні відчуження навколо Чорнобильської АЕС, Україна. Вловлених на забруднених та незабруднених територіях зони відчуження рудих нориць випускали у польові вольєри, які відрізнялися за рівнем радіонуклідів навколишнього середовища для подальшого логітудинального відбору зразків мікробіоти кишечника у тварин з визначеною різницею в забрудненні. Із використанням секвенування 16S рРНК амплікону ми виявили виразні сезонні зміни альфа‐ та бета‐різноманіття. Опромінення, що було в основі цих сезонних коливань, мало значний вплив на бета‐різноманіття. Зокрема, співвідношення Bacillota (= Firmicutes) до Bacteroidota (= Bacteroidetes) (F:B), а також кількість і склад диференційно поширених бактеріальних родин відрізнялися з часом. Вплив радіонуклідів навколишнього середовища не збільшував дисперсію бета‐різноманіття мікробіоти кишечника, всупереч очікуванням принципу Анни Кареніної для мікробіоти. Наші дані демонструють, як короткочасне дослідження може не охопити ширші наслідки впливу полютантів на мікробіоту кишечника диких тварин через природні сезонні зміни в мікробіомі. Також ми підкреслюємо значення довготривалого моніторингу диких тварин для виявлення контекстно‐залежного впливу полютантів на мікробіоту. Вплив радіонуклідів був асоційований зі змінами мікробіоти кишечника дикої рудої нориці, але сезонно обумовленим чином, який, ймовірно, формується взаємодією між рівнем стресу навколишнього середовища, дієтою та фізіологією тварин.

## 1 Introduction

Exposure to environmental pollutants has diverse impacts on wildlife, including a change in the composition of the gut microbiota (Claus et al. 2016; Handy et al. 2023; Jin et al. 2017). For example, rodents (Brila et al. 2021), polar bears (Watson et al. 2021) and birds (Gillingham et al. 2021; Zhang et al. 2023) with elevated levels of heavy metals in their tissues exhibit changes in alpha diversity and community composition of their gut microbiota. Also, reptiles with higher levels of PFAS exhibit compositional changes (an altered ratio of the bacterial phyla Bacillota (formerly Firmicutes) to Bacteroidota (formerly Bacteroidetes)) in their gut microbiota (Beale et al. 2022). Similarly, seabirds that ingest microplastics show changes in gut microbiota composition (Fackelmann et al. 2023).

Despite the substantial empirical evidence that exposure to pollutants can impact the gut microbiota of wild animals, an overlooked aspect is how pollution interacts with natural, temporal changes in gut microbiota composition. Rather than being static, the composition of the gut microbiota of wild animals often varies with season (Gillingham et al. 2025; Marsh et al. 2022; Maurice et al. 2015; Xiao et al. 2019), for example in response to changes in diet (Baniel et al. 2021; Fan et al. 2022; Hicks et al. 2018) or host physiology (Carey et al. 2013; Greene et al. 2022; Kohl et al. 2014; Sommer et al. 2016). Seasonal variation in microbiota composition can impact the outcome of a cross‐sectional study of the association between pollution and gut microbiota. For instance, species of bacteria may differ in their sensitivities to pollutants (Claus et al. 2016) and thus the strength and pattern of any apparent response by the gut microbiota to pollution may depend on the seasonal composition. It is also plausible that the outcome of any analysis of gut microbiota depends on seasonal variation in pollutant availability, for example, because of a shift in the host's diet among items that bioaccumulate pollutants (Ecke et al. 2018) or temporal changes in the amount of pollutant in the environment (Levin et al. 2020).

Temporal variation (i.e., plasticity) in gut microbiota composition is presumably adaptive (Gillingham et al. 2025), principally by facilitating an appropriate response to a change in host physiology or to exploit resources when there is a change in environment (Amato et al. 2015; Baniel et al. 2021; Scholier et al. 2024), although at present there is limited empirical evidence that changes in microbiota composition constitute an adaptive response in wild vertebrates (Bideguren et al. 2024). Nonetheless, it is relevant to determine whether exposure to environmental pollutants affects the temporal trajectory of the gut microbiota as this provides context to cross‐sectional data and/or insights into possible adaptation or acclimation. A stressor that elicits stochastic changes in microbiota composition can be expected to drive an increase in inter‐individual variation. Such increased dispersion (beta diversity) after stress has been called the ‘Anna Karenina principle’ for microbiota (Zaneveld et al. 2017). Alternatively, a stressful event may select for a distinct microbiota composition that is manifest as a temporal reduction in differences among individuals. Assessing how environmental pollution impacts wild animals thus requires a wider context of its interaction with natural, temporal changes in microbiota composition. With longitudinal studies of wild animal gut microbiota remaining rare (Gillingham et al. 2025), our understanding of the impacts of pollution on wildlife gut microbiota is based almost exclusively on cross‐sectional observations (but see Lavrinienko et al. 2020).

Quantifying the effect of environmental pollution on gut microbiota is important for two principal reasons. First, the gut microbiota provide important services, such as interacting with the host's immune system (Hooper et al. 2012; Rooks and Garrett 2016), helping resist colonisation by pathogens (Buffie and Pamer 2013; Caballero‐Flores et al. 2023) and digesting otherwise undigestible food items to provide metabolites and energy via production of short‐chain fatty acids (Louis and Flint 2017; Rowland et al. 2018); substantial changes in the gut microbiota may indicate some disruption to these services associated with pollution. Second, even without rapid changes in host health, specific features of the microbiota may nonetheless provide useful biomarkers of exposure to pollution and thus be used for environmental monitoring (e.g., Montenegro et al. 2020).

Wildlife inhabiting the Chornobyl Exclusion Zone (CEZ) present an excellent model to study the effects of pollution on the gut microbiota. The accident at the Chornobyl nuclear power plant, Ukraine in 1986 released large amounts of persistent radionuclides, including caesium‐137 and strontium‐90, and plutonium‐239 (Kashparov et al. 2018) into the environment, predominantly over northern Ukraine, southeastern Belarus and western Russia (Moller and Mousseau 2006; Mousseau 2021). The CEZ was established around the accident site to limit human exposure to environmental radionuclides, but much wildlife has since recolonised the impacted area (Baker et al. 1996; Deryabina et al. 2015; Webster et al. 2016). Even though more than three decades (the approximate half‐lives of caesium‐137 and strontium‐90) have passed since the accident, large amounts of radioisotopes persist within large areas of the CEZ to elevate the absorbed doses by exposed organisms to a level that is expected to have detrimental biological impacts (Beresford et al. 2020; Cannon and Kiang 2022).

The bank vole *Clethrionomys glareolus* is a small rodent that is an abundant inhabitant of the CEZ that can experience high absorbed doses of radiation (> 1,800 μGy/day) (Baker et al. 2017; Chesser et al. 2000). Exposure to environmental radionuclides has diverse biological impacts on bank voles (Kesäniemi et al. 2020; Kesäniemi, Jernfors, et al. 2019; Kesäniemi, Lavrinienko, et al. 2019; Mappes et al. 2019; Rodgers et al. 2001), including changes in the gut microbiota that manifest as, for example, an increase in the bacterial phyla Bacillota (= Firmicutes) and Proteobacteria (Lavrinienko, Mappes, et al. 2018; Lavrinienko, Tukalenko, et al. 2018). Exposure to radionuclides within the CEZ has no detectable effect on some aspects of gut microbiota function, such as production of faecal short‐chain fatty acids, but impacts markers of host gut condition, such as goblet cell size and mucus production (Jernfors et al. 2024), indicating the biological relevance of exposure to environmental radionuclides on gut microbiota and host health. Indeed, other rodents (i.e., species of *Apodemus* mice) exposed to radionuclides at the Fukushima accident site show changes in their gut microbiota (Lavrinienko et al. 2021). By contrast, a subsequent study found little effect of exposure to radionuclides on the gut microbiota of bank voles inhabiting the CEZ (Antwis et al. 2021). Of the different reasons for the discrepancy between studies (Watts et al. 2022), it is notable that the samples by Lavrinienko, Mappes, et al. (2018) and Antwis et al. (2021) were collected at different times of the year (May/June and August, respectively) raising the possibility that seasonal variation in gut microbiota interacts with exposure to radionuclides. Indeed, Jernfors et al. (2024) uncovered an increase in the gut bacterial phylum Bacteroidota (= Bacteroidetes) in bank voles inhabiting contaminated areas within the CEZ during October.

Interpreting the effect of exposure to radionuclides on wild animals presents numerous challenges. Notably, caesium‐137 has a short biological half‐life (~1.5–4 days) in small mammals (Bondarkov et al. 2002) and thus the radionuclide burden of wild caught animals is dynamic, for example, reflecting recent diet, soil exposure and physiology (Maklyuk et al. 2007). Also, since many animals can move among the matrix of contaminated and uncontaminated areas within the CEZ (e.g., bank voles may disperse several km per year; Kozakiewicz et al. 2007; White et al. 2013) any sample of wild animals from a contaminated or uncontaminated site may contain recent immigrants, not readily identified by the level of whole‐body radiation per se, whose gut microbiota will nonetheless retain differences from that of longer‐term residents (Scholier et al. 2024). The corollary is poor statistical power to identify any effects of a specific habitat and/or its associated pollutant on the gut microbiota. A more powerful approach than empirical sampling alone is to complete longitudinal monitoring of animals in controlled field environments so that their histories of exposure to radionuclides is known with confidence.

Here, we present a temporal study of animals exposed to environmental radionuclides designed to answer the questions: (1) is the apparent impact of exposure to an environmental pollutant contingent upon the time of sampling? (2) does exposure to environmental radionuclides affect the magnitude of any temporal changes in gut microbiota composition, in line with the theoretical framework provided by the Anna Karenina principle for microbiota? To address these knowledge gaps, we caught bank voles from contaminated and uncontaminated areas within the CEZ and released them into field enclosures that differed in level of contamination by radionuclides so that we could longitudinally monitor any changes in gut microbiota composition in controlled conditions. Although the use of field‐based experiments is often challenging (especially during times of armed conflict), it presents a rigorous approach to ensure that animals have been exposed to distinct levels of radionuclide contamination and thus discern the mechanistic bases for variation under natural conditions.

## 2 Materials and Methods

### 2.1 Experimental Design

Bank voles were caught during July 2021 from contaminated and uncontaminated locations within the Chornobyl Exclusion Zone (CEZ) using Ugglan live traps (see Lavrinienko et al. 2020 for methods). Animals were taken to the Chornobyl Research Initiative laboratory within the CEZ where the first samples of faeces were collected and the animals were implanted with subcutaneous microchips for future identification. Bank voles were housed in Makrolon Type III cages (Tecniplast, Italy), provided with *ad libitum* food (Rezon‐1, Ukraine) and water and given sawdust with hay as bedding. Animals were housed for up to 20 days to ensure that gravid females were not released and thus mating took place within the field enclosures.

**FIGURE 1:**
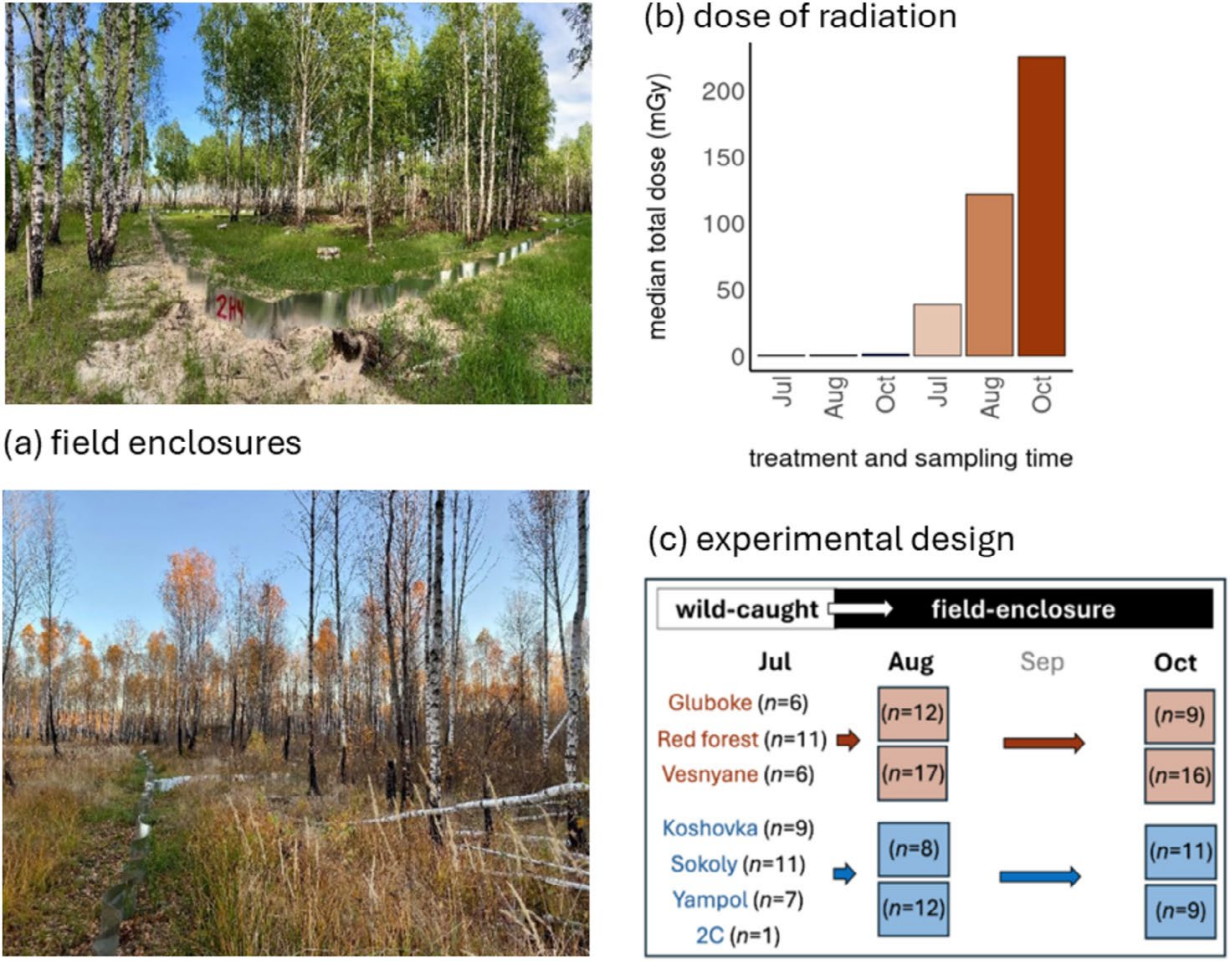
(a–c): (a) Example of seasonal changes (Summer = upper image, and Autumn = lower image) in habitat in experimental field enclosures located within the Chornobyl Exclusion Zone (CEZ), Ukraine (photo credit: Anton Lavrinienko). (b) Median absorbed dose of radiation (mGy) of bank voles ( *Clethrionomys glareolus* ) from either uncontaminated (blue) or contaminated (brown) locations (July) or from field enclosures (August and October) within the CEZ. (c) Overview of experimental design and sampling sizes, with wild‐caught bank voles from contaminated (brown) and uncontaminated (blue) locations within the CEZ (sampled during July) transferred to experimental field enclosures for subsequent sampling in August and October. Sample sizes and median doses of radiation are given in Table S1.

Bank voles were released into experimental field enclosures (Figure 1a) (four males and six females in each of the 16 enclosures) that were distributed among four locations within the CEZ—two locations on contaminated soils and two locations in uncontaminated areas—to provide experimental sites that differed in ambient dose rate from 0.07–0.11 μGy/h in uncontaminated areas and from 44.43–178.6 μGy/h in the contaminated locations (Figure 1b, Table S1). Animals were released into an enclosure that reflected the same experience of radionuclide pollution as the habitat from which they were trapped (i.e., animals from contaminated locations were released into contaminated enclosures, and those from uncontaminated habitats released to uncontaminated enclosures) After 18–21 days in the field enclosures, the surviving animals were recaptured to take a second faecal sample and so that females could give birth in the animal facility. Offspring were cross‐fostered among litters irrespective of their origin (while maintaining the mother's litter size) to prevent the enclosures from being dominated by one or two families. Survival of the cross‐fostered offspring did not significantly (*χ* ^2^ = 0.044, df = 1, *p* = 0.834) differ to that of offspring who remained with their birth mother. Females and their litters were returned to the enclosure and trapping location from which the female had been caught (as females will return their litter to their territorial burrows for nursing). Males were not returned to the field enclosures as male bank voles can exhibit aggressive behaviour towards females and offspring (Ylönen et al. 1997). The surviving animals were recaptured in October (i.e., after 48–60 days as male and female bank voles attain maturity at 4–8 weeks) to collect samples of faeces from a third point in time. Thus, we quantified the gut microbiota of bank voles from two treatments (contaminated and uncontaminated areas) at three times in the year (see Figure 1a for example change in habitat between Summer and Autumn, and Figure 1c for overview of sample sizes and experimental design).

### 2.2 Estimation of Internal, External and Total Absorbed Radiation Doses

External dose rates were quantified by measuring ambient radiation levels with a hand‐held Geiger counter (Gamma‐Scout, GmbH & Co, Germany) placed 1 cm above the ground, with measurements made at least nine times at each trapping location within the CEZ (from where we sourced the adults) and > 160 times within each field enclosure (to provide a 5–10 m resolution of the level of external dose rate). External dose rate at a trapping location predicts the recent (within approximately 24 h) caesium‐137 activity of small mammals inhabiting the CEZ (Chesser et al. 2000; Lavrinienko et al. 2020). Average external dose rate was calculated for each trapping location (in the field and in the enclosures).

Internal dose rate was estimated using a SAM 940 radionuclide identifier system (Berkeley Nucleonics Corporation, USA) equipped with a 3″ × 3″ NaI detector that measured caesium‐137 activity. The detector was enclosed in 10 cm lead shielding to reduce any signal from background radiation. The system was calibrated with reference standard sources. With corrections for background radiation, caesium‐137 activities of the whole animals were evaluated from the obtained spectra in the energies range 0.619–0.743 MeV (with photopeak of caesium‐137 at 0.662 MeV) with the use of a phantom with known activity, and geometry similar to the bank vole body. Individual body mass was used to standardise the estimated dose across animals. Further details of dosimetry calculations are provided in the Methods in Supporting Information.

Total absorbed doses of animals were calculated as the sum of the internal and external absorbed dose rates multiplied by the time (days) spent at the radiation exposure treatment (i.e., the estimated age of the founder animals and the subsequent number of days spent in the field enclosures). We approximated the ages of the wild‐caught animals using the following age‐size relationship for bank voles: head width (mm) = 8.4831 × age (days)^0.0928^ (Kallio et al. 2014). Animals from uncontaminated and contaminated treatments (field locations or enclosures) experienced significantly different (Dunn's test, *p* < 0.003 for all pairwise comparisons) absorbed doses of radiation at all sampling times (Table S2), with the median absorbed dose for animals in uncontaminated areas (0.5–1.6 mGy) being significantly (Table S2) less than the median absorbed dose of radiation experienced by animals inhabiting contaminated areas (39–225 mGy) at all three sampling times (Figure 1b, Table S1).

### 2.3 DNA Extraction and Amplicon Sequencing

We used a PowerSoil Pro DNA kit (Qiagen) to extract DNA from faecal samples, along with two negative controls in which water replaced the samples. Library preparation and amplicon sequencing were performed at the University of Jyväskylä using an Illumina MiSeq (300 bp paired‐end reads) and the PCR primers 515F and 806R (Apprill et al. 2015; Parada et al. 2016) to amplify the V4 region of the 16S rRNA.

Read data were imported into *qiime2* v.2023.5 (Bolyen et al. 2019), and primer sequences were removed using the *cutadapt* plugin (Martin 2011). Data were denoised using *DADA2* (Callahan et al. 2016) (dada2 denoise paired parameters: –p‐trunc‐len‐f 220 –p‐trunc‐len‐r 200). To reduce the amount of artificially split bacterial genomes (Schloss 2021), the amplicon sequence variants clustered at 97% identity (vsearch cluster‐features‐de‐novo parameters: –p‐perc‐identity 0.97) using the *vsearch* (Rognes et al. 2016) plugin for *qiime2*. Taxonomy was assigned to the features using a naive Bayes classifier (Bokulich, Dillon, et al. 2018) and a reference database trained on SILVA v.138_99 bacterial V4 16S rRNA sequences (Yilmaz et al. 2014). We removed the features whose taxonomy was unassigned, as well as those features that were classified as either mitochondria, chloroplasts, Archaea, or eukaryotes (qiime taxa filter‐table parameters: –p‐include p__ –p‐exclude Archaea, Mitochondria, Chloroplast, Eukaryota) and then constructed a mid‐point rooted phylogenetic tree using *fasttree* (Price et al. 2009) (qiime phylogeny align‐to‐tree‐mafft‐fasttree). The feature table, phylogenetic tree, taxonomic classification and metadata were imported into *phyloseq* v.1.40.0 (McMurdie and Holmes 2013) and likely contaminant features removed using the prevalence method (threshold = 0.5) implemented by *decontam* v.1.16.0 (Davis et al. 2018). The filtered data comprised 3,667,994 reads from 174 faecal samples.

### 2.4 Statistical Analyses

Except where stated (e.g., using *qiime2* plugins), statistics were performed using *R* v.4.4.0 (R Core Team 2025) and graphics produced by *ggplot2* (Wickham 2009). For analyses of alpha and beta diversity, the feature table was even sampled to 12,010 reads per sample as rarefaction curves had plateaued well below this threshold (Figure S1b), indicating adequate sampling of microbial diversity, and we could retain 84% of the samples (Figure S1a) while losing just 90 features. After this, there were read data from 52 (*n* = 23 and *n* = 29 for contaminated and uncontaminated source locations respectively), 49 (*n* = 29 and *n* = 20 for contaminated and uncontaminated enclosures) and 45 (*n* = 25 and *n* = 20 for contaminated and uncontaminated enclosures) bank voles during the first (July), second (July–August) and third (October) sampling times respectively (Figure 1c, Table S1). Following the recommendations of Schloss (2024a, 2024b), we performed even sampling 100 times and used the mean values of estimates of alpha and beta diversity for statistical analyses. Alpha diversity was calculated as observed richness and Shannon's index (evenness) in *phyloseq* and as Faith's phylogenetic diversity (Faith 1992) in *picante* v.1.8.2 (Kembel et al. 2010). Principal drivers of variation n alpha diversity were analysed using generalised linear mixed‐effects models (GLMMs) using the package *glmmTMB* v.1.1.9 (Brooks et al. 2017; McGillycuddy et al. 2025). Observed features were rounded to the nearest integer and modelled using a negative binomial GLMM; Shannon and Faith's phylogenetic diversities were modelled using Gaussian GLMMs and Gamma GLMMs with a log link, respectively. Fixed effects included Treatment (contaminated or uncontaminated locations) and Time (sampling during July, August, or October) and their interaction, with Sex and Body Mass (standardised weight, as a proxy for variation in age and body condition of animals) included as covariates; we included individual identity and sample location (capture location or enclosure) as random effects. We also modelled the main effect of time (with location and identity as random effects) on alpha diversity as other predictors had no significant effect (Table S3a,b). Estimates of marginal *R* ^2^ and conditional *R* ^2^ were calculated with *performance* v.0.11.0. (Lüdecke et al. 2021) and fit with model assumptions (e.g., assessments of dispersion, zero inflation and outliers) were examined and confirmed using *DHARMa* v.0.4.6 (Hartig 2025) (see Figures S3a–c and S4a–c for output). Beta diversity was estimated using Jaccard's, Bray‐Curtis and UniFrac (weighted and unweighted) (Lozupone et al. 2011) metrics, with variation among individuals and treatments visualised using Principal Coordinates Analysis (PCoA). Possible predictors of variation in beta diversity examined were radionuclide contamination (Treatment, as a binary variable contaminated/uncontaminated habitats, and the continuous variable of absorbed dose), sampling time, sex and body mass (*z*‐score standardised weight to a mean of zero and a standard deviation of one), whilst accounting for individual identity and sample location (capture location or enclosure); statistical significance of any difference in beta diversity was analysed using permutational multivariate analysis of variance (PERMANOVA) implemented with the adonis2 function in the *R* package *vegan* v.2.6.4 (Oksanen et al. 2001). To examine whether the effect of exposure to radionuclides varied over time, we fitted a model including an interaction between treatment and time (distance~Treatment × Time + Sex + Body Mass); to assess the main effects of each predictor, we fitted a model without the interaction term (distance~Treatment + Time + Sex + Body Mass). All models were run with 9999 permutations using marginal tests (by = “marginal”), and with the permutations restricted within individuals and blocked by the capture location or enclosure.

As previous studies indicated that exposure to environmental radionuclides may (Lavrinienko, Mappes, et al. 2018) or may not (Antwis et al. 2021) impact the ratio of the phyla Bacteroidota (= Bacteroidetes) to Bacillota (= Firmicutes) we calculated the centred log ratio (CLR) of Bacteroidota:Bacillota (but retained the term F:B for Firmicutes:Bacteroidetes) for samples in uncontaminated and contaminated locations at each time, and used a Gaussian GLMM (package *glmmTMB*) to determine significance of treatment, sampling time, sex and body weight (as defined above) on the CLR of F:B, after confirming fit with model assumptions (Figure S6).

Homogeneity of dispersion in the four metrics of beta diversity was calculated using betadisper in *vegan*, with the permutest function (9999 permutations) used to determine the significance of any differences in dispersion between uncontaminated and contaminated treatments and also at each of the three sampling times and among the composite groups of Treatment × Time (to reflect an interaction between these predictors). Since the Anna Karenina principle for microbiota (Zaneveld et al. 2017) predicts an increase in beta diversity with time since exposure to a stressor, we examined whether there was a difference in the mean or dispersion of the temporal changes in beta diversity within adults (as juveniles were sampled only once in October) between successive sampling times (of July, August and October); this is equivalent to calculating first distances (Bokulich, Kaehler, et al. 2018). We modelled these temporal distances using generalised linear mixed models (GLMMs) using *glmmTMB* v.1.1.9 (Brooks et al. 2017; McGillycuddy et al. 2025), with treatment, time and their interaction as fixed effects, and bank vole's individual identity and sampling location included as random effects to account for repeated measures and site‐level variation (except for analyses of the Bray‐Curtis metric, where the models did not converge unless the sampling location random effect was dropped). To assess changes in dispersion, we modelled the absolute deviation of each observation from its group mean. Model diagnostics (dispersion, zero inflation and outliers) were evaluated using *DHARMa* v.0.4.6 (Hartig 2025) (see Figures S8a–d and S9a–d).

Finally, we used *ancom‐bc2* v.1.6.4 (Lin and Peddada 2020, 2024) (parameters: fix_formula = “Treatment × Time + Location”, *p*_adj_method = “fdr”, pseudo = FALSE, pseudo_sens = TRUE, prv_cut = 0.01, lib_cut = 4800, struc_zero = FALSE, neg_lb = FALSE, alpha = 0.05) on the unrarefied feature table to calculate differential abundance of bacterial genera in response to the absorbed total dose (mGy^−1^) while allowing for an interaction between exposure to radionuclides and the time in year. We included location (capture or enclosure) as a fixed effect because the sample size in some locations was too small to allow a reliable estimate of random‐effect variance components (and the model would not converge).

## 3 Results

**FIGURE 2:**
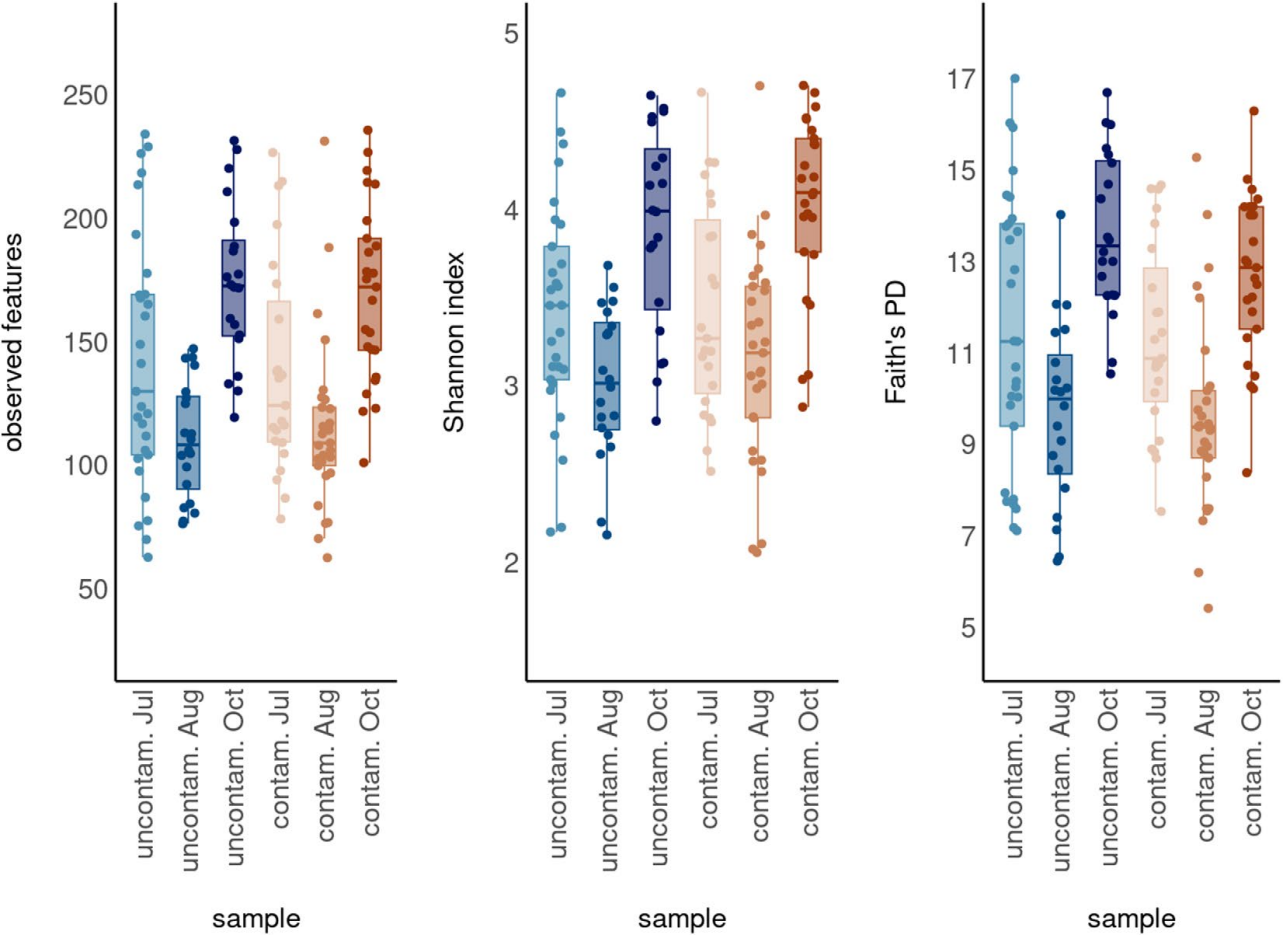
Temporal variation in three indices of alpha diversity (observed features, Shannon's index and Faith's phylogenetic diversity) of the gut microbiota of bank voles ( *Clethrionomys glareolus* ) from either contaminated or uncontaminated locations (July) or field enclosures (August and October) in the Chornobyl Exclusion Zone (CEZ), Ukraine. See Table S3a–c for statistical significance of changes in alpha diversity among samples.

Alpha diversity (observed features, Shannon index and Faith's phylogenetic diversity) of the bank vole gut microbiota varied significantly (*p* < 0.001) among sampling times (Table S3a), being lowest in August and highest in October, while other predictors (contamination, sex and body mass) had no significant effect and nor was there a significant interaction between Treatment (= contamination) and Time (Table S3a,b). The non‐significant (Table S3b) reduction in alpha diversity between July and August was significant when Time was modelled alone (i.e., in models that excluded the non‐significant predictors of treatment, sex and body mass) (*cf*. Table S3b,c). These data indicate that seasonal change, rather than radionuclide exposure, was the primary driver of shifts in bank vole gut bacterial alpha diversity, explaining about 29% of the variation (Figure 2, Table S3a–c).

**FIGURE 3:**
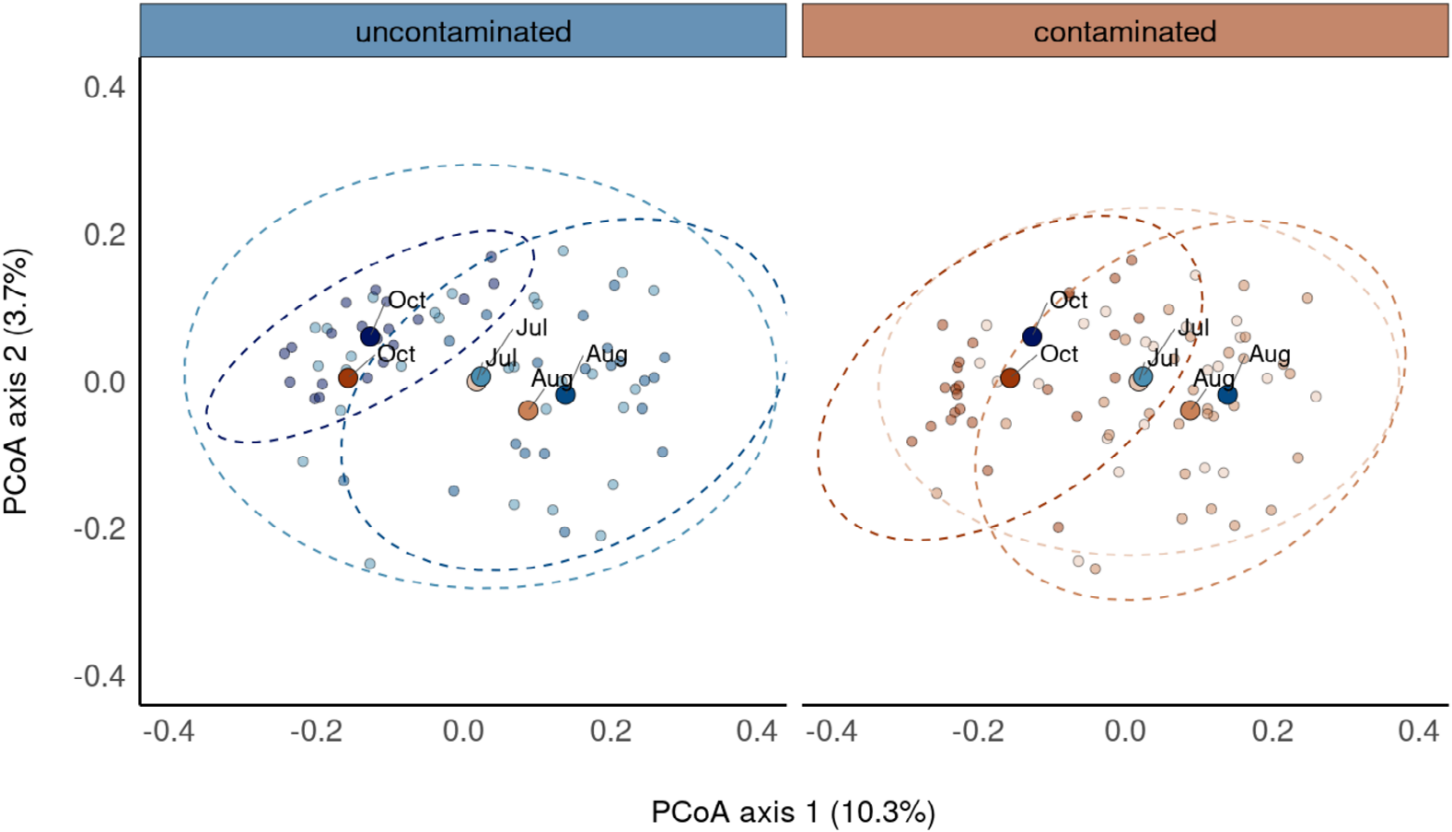
Temporal (during July, August and October) differences in beta diversity (Jaccard's dissimilarity) of gut bacteria from bank voles ( *Clethrionomys glareolus* ) inhabiting contaminated and uncontaminated areas (July) or field enclosures (August and October) located within the Chornobyl Exclusion Zone (CEZ), Ukraine. Individual samples and confidence ellipses are shown in separate, faceted panels by the level of radionuclide contamination for clarity, while group centroids are displayed in both panels to facilitate comparison of group‐level changes (see Table 1 for summary of PERMANOVA analyses). Principal coordinates analysis (PCoA) visualisations of three other metrics of beta diversity (Bray Curtis dissimilarity, and unweighted and weighted UniFrac metrics) are given in Figure S5a–c.

A significant amount of variation in beta diversity of bank vole gut microbiota among samples was attributed to sampling Time and Treatment (contaminated or uncontaminated locations) in additive models (*p* ≤ 0.003 for both predictors and all four metrics of beta diversity; Table 1, Table S4a, Figure 3, Figure S5a–c). When modelled as a continuous variable (i.e., total dose), time remained a significant predictor (*p* < 0.001) of variation in beta diversity but the effect of exposure to radionuclide was weak, with significant (*p* = 0.028, unweighted UniFrac) or non‐significant (*p* ≥ 0.06 for Jaccard, Bray‐Curtis, and weighted UniFrac metrics) effects detected (Table 1, Table S4b). For all analyses of beta diversity, we found no clear evidence for a significant interaction between Treatment and Time (*p* = 0.048 for Jaccard's index, and *p* > 0.05 for the other measures of beta diversity; Table 1, Table S4a,b). While many of the models uncovered sex as a significant predictor of variation in bank vole gut microbiota beta diversity, the effect size was weak (*R* ^2^ < 0.007) (Table 1). Rather, the dominant effect on bank vole gut microbiota beta diversity was associated with variation in sampling time (*R* ^2^ = 0.05–0.07), with an apparently lesser impact of exposure to environmental radionuclides (*R* ^2^ ~ 0.01 for the binary classification of radionuclide exposure) (Table 1).

**TABLE 1:** Statistical significance (by PERMANOVA) and effect sizes of exposure to environmental radionuclides (treatment = contaminated or uncontaminated areas, or absorbed dose) and temporal changes (Time = sampling during July, August, or October) on variation in beta diversity of gut bacteria from the bank vole ( *Clethrionomys glareolus* ) inhabiting contaminated and uncontaminated areas (July) or field enclosures (August and October) located within the Chornobyl Exclusion Zone (CEZ), Ukraine.

Predictor	cont./uncont.		Total dose (mGy)	
	*R* ^2^	*p*	*R* ^2^	*p*
(a) Jaccard				
Treatment	0.013	< 0.001	0.013	0.068
Time	0.052	< 0.001	0.050	< 0.001
Sex	0.007	< 0.001	0.007	0.001
Body mass	0.009	0.436	0.010	0.394
Residual	0.902		0.902	
Total	1.000		1.000	
(b) Bray‐Curtis				
Treatment	0.009	0.003	0.011	0.314
Time	0.061	< 0.001	0.056	< 0.001
Sex	0.004	0.017	0.005	0.022
Body mass	0.012	0.236	0.013	0.243
Residual	0.885		0.883	
Total	1.000		1.000	
(c) Unweighted UniFrac				
Treatment	0.014	0.002	0.015	0.028
Time	0.060	< 0.001	0.058	< 0.001
Sex	0.006	0.002	0.006	< 0.001
Body mass	0.008	0.375	0.010	0.273
Residual	0.889		0.888	
Total	1.000		1.000	
(d) Weighted UniFrac				
Treatment	0.007	0.003	0.009	0.061
Time	0.073	< 0.001	0.068	< 0.001
Sex	0.004	0.045	0.004	0.043
Body mass	0.010	0.321	0.012	0.283
Residual	0.870		0.868	
Total	1.000		1.000	

*Note:* PCoA visualisations of beta diversity are given in Figure 3 (Jaccard's metric) and Figure S5a–c (all other metrics).

Abbreviations: *p*, statistical significance; *R* ^2^, effect size.

There was no strong support that exposure to environmental radionuclides drives a change in the level of microbiota dispersion (as predicted by the Anna Karenina principle for microbiota). Thus, there were few (*p* < 0.05 for only 2 out of the 12 tests over all four metrics of beta diversity) significant differences in dispersion among the uncontaminated and contaminated samples at the three sampling times (Table S5a). There were some significant differences in dispersion among the treatment, time and their interaction (Treatment × Time); these were not consistently associated with a specific predictor for all measures of beta diversity (Table S5b). Moreover, there were no significant differences (*p* > 0.05 for all tests) in either the mean (Table S6a) or dispersion (Table S6b) of the temporal change in beta diversity within individual bank voles from uncontaminated and contaminated enclosures (Figure S7).

**FIGURE 4:**
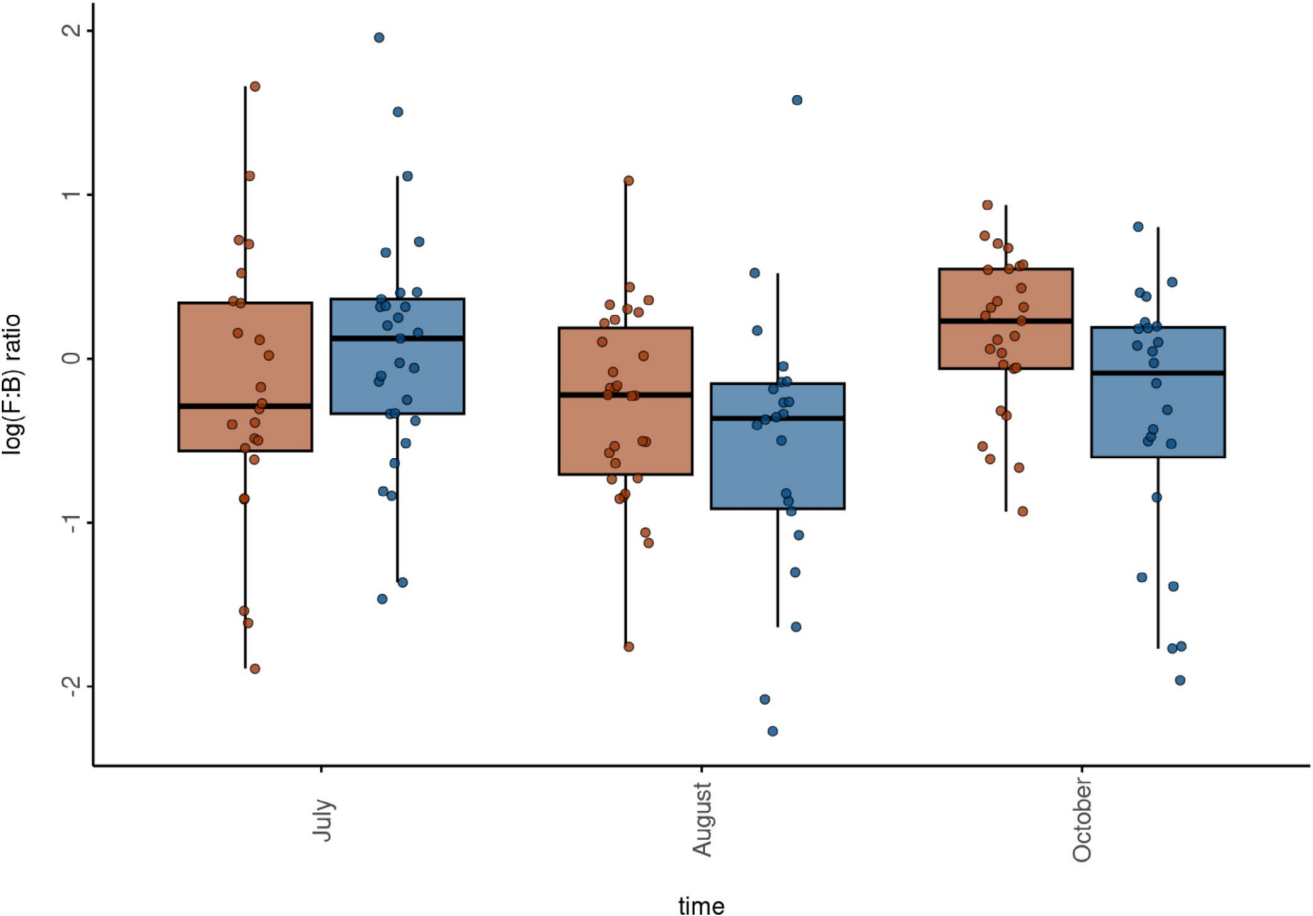
Temporal variation in the ratio of the phyla of gut bacteria Bacillota (= Firmicutes) and Bacteroidota (= Bacteroidetes) (F:B ratio calculated as centre log ratio, CLR) from bank voles ( *Clethrionomys glareolus* ) inhabiting contaminated (brown) and uncontaminated (blue) areas (July) or field enclosures (August and October) located within the Chornobyl Exclusion Zone (CEZ), Ukraine. A CLR ratio of 0 indicates an F:B ratio of 1. See Table 2 for statistical significance of variation in CLR among samples.

Bank vole gut bacteria were dominated by the two phyla Bacteroidota (= Bacteroidetes) and Bacillota (= Firmicutes), which accounted for an average relative proportion of 0.49 and 0.43 of the read data, and with minor contributions of Spirochaetota (average relative proportion = 0.01) and Actinobacteriota (average relative proportion = 0.03) (Figure S2, Table S7). Bacillota were dominated by the families *Lactobacillaceae*, *Lachnospiraceae*, *Christensenellaceae* and *Oscillospiraceae*, while the Bacteroidota were dominated by the family *Muribaculaceae* (Figure S2, Table S7). Consistent with the significant effect of time in driving changes in alpha and beta diversity, there was a notable main effect of sampling time on the abundance of the bank vole gut bacteria phyla, orders and families (Figure S2) and a secondary effect of exposure to environmental radionuclides; for example, the relative proportions of *Lactobacillaceae* (the most abundant Bacillota family) exhibited a seasonal decline in both areas (but more so in uncontaminated locations), while the *Lachnospiraceae* (the second most abundant Bacillota family) increased in abundance during October and the *Muribaculaceae* (Bacteroidota) decreased more in the contaminated than uncontaminated areas between August and October. The outcome of these seasonal changes in taxonomic composition is that the F:B ratio is higher in bank voles inhabiting uncontaminated areas than contaminated areas in July, but not in August and October when the reverse pattern occurs (Figure 4). Hence, the F:B ratio of bank voles is not only significantly (*p* < 0.04) affected by sampling time, but there is a significant interaction (*p* = 0.007 during October) between sampling time and exposure to radionuclide contamination (Table 2).

**TABLE 2:** Statistical significance of generalised linear mixed effects model (GLMM) that examine predictors of variation in the ratio of the phyla of gut bacteria Bacillota (= Firmicutes) and Bacteroidota (= Bacteroidetes) (F:B ratio calculated as centre log ratio, CLR) from bank voles ( *Clethrionomys glareolus* ) inhabiting contaminated and uncontaminated areas (July) or field enclosures (August and October) located within the Chornobyl Exclusion Zone (CEZ), Ukraine.

Predictor	Est.	SE	CI lower	CI upper	*p*
Intercept	0.053	0.130	−0.202	0.308	0.683
Treatment (contaminated)	−0.251	0.193	−0.630	0.128	0.194
Time (Aug)	−0.587	0.198	−0.975	−0.199	0.003
Time (Oct)	−0.403	0.193	−0.782	−0.025	0.037
Treatment (contaminated):Aug	0.498	0.276	−0.042	1.038	0.071
Treatment (contaminated):Oct	0.748	0.276	0.208	1.288	0.007
Marginal *R* ^2^	0.098				
Conditional *R* ^2^	0.098				

Abbreviations: CI lower/CI upper, lower and upper confidence intervals; Est., estimate; *p*, statistical significance; SE, standard error.

**FIGURE 5:**
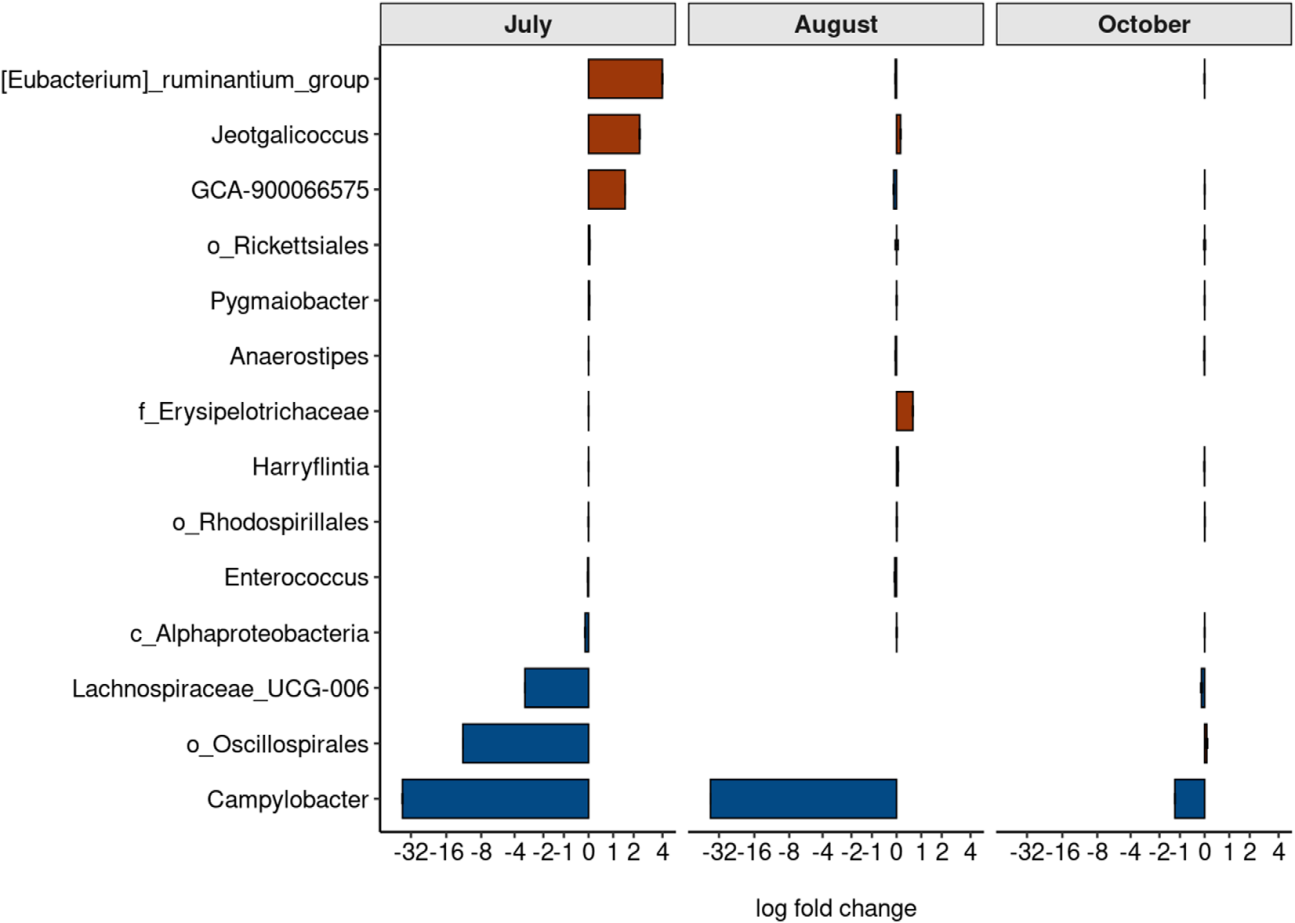
Temporal (over July, August and October) differences in the genera of gut bacteria from the bank vole ( *Clethrionomys glareolus* ) that are significantly differentially abundant in response to exposure to elevated levels of radiation (log fold change in abundance per mGy total dose). Brown bars indicate an increase in abundance with dose of radiation, whereas blue bars indicate a decrease in abundance with dose.

In line with the changes in taxonomic composition over time and in response to exposure to radionuclides, distinct genera of gut bacteria were significantly (*p* < 0.05 after fdr correction) differentially abundant in response to absorbed dose in July, August and October (Table S8), consistent with a significant dose by time interaction. However, both the magnitude of the association between dose and abundance and the number of differentially abundant genera differed between sampling times (for instance, with 9, 10 and 7 differentially abundant genera identified during June, August and October, respectively) (Figure 5, Table S8). Thus, the taxonomic composition and identity of the significantly differentially abundant bacterial genera as a response to exposure to environmental radionuclides depend upon the sampling time (Figure 5), in line with seasonal changes in composition or abundance of specific taxonomic groups (Figure 4, Figure S2).

## 4 Discussion

While exposure to environmental pollutants can disrupt the gut microbiota, it is not known whether pollutants impact the natural, seasonal changes in wildlife gut microbiota. By longitudinal monitoring of wild bank voles ( *Clethrionomys glareolus* ) inhabiting contaminated and uncontaminated areas within the Chornobyl Exclusion Zone (CEZ), Ukraine, we found (1) marked temporal changes in gut microbiota and (2) a smaller effect of exposure to environmental radionuclides on the gut microbiota, with the outcome that (3) the apparent effect of inhabiting a contaminated area is contingent upon the time in the year during which samples were collected.

Marked temporal changes in the bank vole gut microbiota are consistent with other longitudinal studies of wild animals (Baniel et al. 2021; Fan et al. 2022; Hicks et al. 2018; Xiao et al. 2019), including rodents (Marsh et al. 2022; Maurice et al. 2015). Despite the diversity of functions among different bacterial features (Bedu‐Ferrari et al. 2024; Lozupone et al. 2012), such seasonal variation in gut microbiota can manifest as differences in the ratio of the dominant phyla of gut bacteria. In some mammals, the proportion of Bacillota (= Firmicutes) increases at the expense of Bacteroidota (= Bacteroidetes) when resources are scarce such as during winter (Jiang et al. 2021; Wang et al. 2024; Xia et al. 2021) or in the dry season (Chen et al. 2023; Gomez et al. 2016; Springer et al. 2017). In the mammalian gut, Bacillota and Bacteroidota ferment polysaccharides (Flint et al. 2012) but with somewhat divergent roles. Bacteroidota tend to be the primary degraders of soluble fibre (e.g., starch, pectin, xylan, hemicellulose) (Bedu‐Ferrari et al. 2024; Lapébie et al. 2019) but also feed on host‐derived glycans (Marcobal et al. 2013; Ormerod et al. 2016; Zafar and Saier 2021). Bacillota often utilise insoluble fibres (e.g., cellulose, resistant starch, long‐chain inulin) or oligosaccharides released by the primary degraders (Bedu‐Ferrari et al. 2024). A greater proportion of Bacillota (higher F:B ratio) increases the amount of energy harvested from the diet in humans and in animal models. The natural reduction in the proportion of Bacillota in summer and Autumn would be consistent with seasonal changes in bank vole diet (e.g., Ecke et al. 2018; Viro and Sulkava 1985) and physiology.

We have previously demonstrated that bank voles exposed to radionuclides experience an altered gut microbiota, notably via an increased proportion of Bacillota (Lavrinienko, Mappes, et al. 2018; Lavrinienko, Tukalenko, et al. 2018). Our longitudinal data, however, provide the important, new perspective that the impact of radionuclide exposure on the F:B ratio differs throughout the year. An elevated (in October) F:B ratio in bank voles exposed to radionuclides (compared with animals from uncontaminated areas) could imply some need to increase energy harvest in response to physiological demands imposed by inhabiting a polluted environment; for example, some animals inhabiting the CEZ exhibit signs of oxidative stress (Bonisoli‐Alquati et al. 2010), and bank voles exposed to radionuclides show changes in their metabolism (Kesäniemi, Jernfors, et al. 2019) which may reflect an energetic investment towards cellular detoxification, maintenance and repair (Kesäniemi et al. 2020; Kesäniemi, Lavrinienko, et al. 2019). It is also possible that changes in gut community reflect temporal differences in radionuclide burden (Maklyuk et al. 2007) and thus the absorbed dose (Figure 1b). Alternatively, since radionuclide contamination can affect the abundance of insects (Møller and Mousseau 2009) and plant germination (Boratyński et al. 2016), there is a likely seasonal impact on the gut microbiota via differences in the seasonal availability and diversity of the bank vole's diet that occur between contaminated and uncontaminated areas. Deconfounding the direct effects of radionuclide exposure on the gut microbiota from likely indirect impacts (such as a change in diet) present an interesting avenue for further research and could be examined, for example, using a combination of field enclosures and supplemental feeding (e.g., Mappes et al. 2019) to reduce any effect of variation in diet between contaminated areas. Irrespective of the underlying driver(s), exposure to radionuclides nonetheless associates with a change in gut microbiota community composition of wild animals, comparable with the impact of other pollutants (Beale et al. 2022; Brila et al. 2021; Teffera et al. 2024; Watson et al. 2021; Zhang et al. 2023).

Beneath the broad community/taxonomic changes associated with season are specific features that are differentially abundant in response to environmental radionuclide contamination. An interesting aspect to these data with regard to the notable changes in F:B ratio is the identification of differentially abundant taxa within the Bacillota (e.g., *Enterococcus*, *Anaerostipes*, *Eubacterium*, *Lachnospiraceae* (UCG‐006 and GCA‐900066575), *Harryflintia*, *Jeotgalicoccus*, Erysipelotrichaceae and Oscillospirales) but not members of the Bacteroidota (as other differentially abundant taxa are members of Proteobacteria and Proteobacteria). This apparent discrepancy between the two dominant phyla within the rodent gastrointestinal tract highlights functional diversity within the Bacillota and that diversity of functional groups within the Bacteroidota (Lagkouvardos et al. 2019; Ormerod et al. 2016) is not captured by higher‐level taxonomic classification, either at the level of genera or because the 16s v4 region cannot provide genus/species‐level taxonomic resolution for Bacteroidota, at least in wild animals. Importantly, the number and taxonomic composition of differentially abundant features differs with times. The lowest number of differentially abundant taxa in October is interesting as this is the period of greatest alpha diversity and potentially indicates an increasingly dominant effect of host diet or physiology on gut microbiota during autumn‐winter compared with the effects of pollution earlier in the year, consistent with a winter increase in certain bacteria taxa (e.g., certain Bacillota such as *Ruminococcaceae*) and convergence of gut microbiota in rodents (Marsh et al. 2022).

Evidence that exposure to environmental pollutants elicits stochastic changes in the gut microbiota in wild animals (and a temporal increase in dispersion), in line with the Anna Karenina principle for microbiota (Zaneveld et al. 2017), is equivocal. That exposure to environmental radionuclides did not affect the level of inter‐individual heterogeneity in gut microbiota in our data contrasts with the homogenising effect of pesticides on the honeybee gut microbiota (Cuesta‐Maté et al. 2021) and with a prior study on bank voles whereby the animals inhabiting contaminated areas within the CEZ had a more stable gut microbiota over time (Lavrinienko et al. 2020). Reasons for the differences between these studies on bank voles inhabiting the CEZ are not known, but Lavrinienko et al. (2020) took samples over a shorter period of time and earlier in the season (May and June) than the current experiment (July–October) indicating that the degree of plasticity exhibited by the gut microbiota depends on the season and/or the length of time of the study. With few studies using longitudinal monitoring of gut microbiota of wild animals exposed to pollutants, it remains unclear whether the Anna Karenina principle for microbiota would be a typical response to pollution by wildlife. In humans, only about half of the microbiota associated with diseases meet the predictions of the Anna Karenina principle for microbiota (Ma 2020). Our data indicate that over a period of several months, the gut microbiota retains a capacity for seasonal change in both contaminated and uncontaminated sites.

From an environmental monitoring perspective, our study highlights the need for greater spatiotemporal resolution in ecotoxicological studies of wildlife (Brand et al. 2025) by demonstrating how a single cross‐sectional study is unlikely to fully capture the impact of pollution on gut microbiota and might even be misleading. An important corollary of seasonal variation in gut microbiota is that different features of the gut microbiota are differentially abundant among sampling times. Indeed, Lavrinienko, Mappes, et al. (2018) and Jernfors et al. (2024) reported increases in the proportions of Bacillota and Bacteroidota respectively in bank voles taken from contaminated sites. As these were separate studies conducted during different years, it was not clear whether the difference was, for example, due to turnover of the bank vole populations or differences in sample locations, rather than seasonal changes in microbiota occurring in resident animals. Our temporal monitoring of animals confirms that there is a detectable impact of inhabiting a habitat contaminated by radionuclides on the gut microbiota but that this effect is contingent on season. This context dependency is overlooked in studies of environmental impacts on gut microbiota and requires further study. Crucially, there might be certain periods in an animal's life when certain features of the environment (e.g., habitat, diet) and/or physiology (e.g., during reproduction, as developing animals, and/or hibernation) exert a greater impact on the gut microbiota than pollution. As an example, there are few differences in F:B ratio in response to radionuclide pollution in August, which coincides with the timing of Antwis et al.'s (2021) sampling of bank voles in which they found no effect of absorbed dose on the F:B ratio. Sampling animals from experimental field enclosures overcomes many of the challenges of interpreting data that differ, for instance, in sampling protocol, sample locations and sampling dates. Rather than any discrepancy among studies, the cross‐sectional data collected by Lavrinienko, Mappes, et al. (2018), Antwis et al. (2021) and Jernfors et al. (2024) appear to be remarkably consistent with the seasonal variation in gut microbiota uncovered by this study, under controlled conditions. The apparent consistency of these previous studies with our data implies that it may be possible to combine data from several cross‐sectional studies to gain an insight into how environmental pollutants affect gut microbiota over time (e.g., for future meta‐analyses). Nonetheless, until other studies can confirm this, robust assessments of the impacts of pollutants on gut microbiota should, where possible, implement longitudinal monitoring. Importantly, seasonal variation in gut microbiota poses a clear challenge to the use of gut microbes as simple biomarkers of exposure to pollutants.

It is important to note that the Chornobyl radioactive landscape is dynamic with large temporal and spatial heterogeneity within the exclusion zone. First, it has been almost four decades since the accident (in 1986), with overall levels of radioactive contamination gradually declining during this period providing ample time for evolutionary responses in the bank vole populations and their symbionts. Second, the radioactive landscape is highly spatially variable with “islands” of high and low contamination distributed randomly within and beyond the CEZ. These two factors make it likely that evolutionary responses have, and will continue to have, affected population‐level traits and ecological interactions, although few rigorous studies have addressed this issue (Møller and Mousseau 2016). The one experimental study of adaption to date suggests that some microbiota may have evolved adaptative responses to the radioactivity (Ruiz‐González et al. 2016) which highlights the need for an understanding of the relative importance of acute versus chronic environmental perturbations on populations.

Although the direct impacts of exposure to pollutants on a host and its microbiota can be measured using laboratory studies (e.g., Liang et al. 2019; Meng et al. 2022; Tang et al. 2020), it is important to examine impacts in natural habitats since wild animals are exposed to diverse stressors that cannot be replicated in the laboratory (such as competition, parasites; Turko et al. 2023) and which may lower the threshold dose at which the pollutant exerts a biological impact. For example, wildlife inhabiting the CEZ are more radiosensitive than animals in controlled irradiation experiments (Garnier‐Laplace et al. 2013). Wild animals have a dynamic gut microbiota (Gillingham et al. 2025), however, and the degree of plasticity of the gut microbiota composition may be a key feature of animal health (e.g., Zaneveld et al. 2017). Even though exposure to radionuclides can elicit changes in gut microbiota composition, this apparent effect of pollution did not have a major impact on the extent of temporal change in beta diversity likely reflecting the greater effect size of seasonal changes on the gut microbiota than radionuclides. Further longitudinal studies that assess whether pollutants impact microbiota plasticity would be informative. For instance, given that gut microbiota exhibit major changes during development (Hanski et al. 2024; Schoultz et al. 2025), a key next step would be to determine whether pollution exposure during early life or in late life elicit a similar impact on microbiota assembly and subsequent plasticity. Nonetheless, our experiment demonstrates how a single cross‐sectional study cannot fully capture the effect of exposure to pollution on wildlife gut microbiota.

## Author Contributions

Designed research (A.V., E.T., A.L., T.M., P.C.W.), performed research (A.V., E.T.), contributed reagents or analytical tools (T.A.M., T.M., P.C.W.), completed laboratory work (A.V.), analysed data (A.V., P.C.W.), wrote the main paper (A.V., P.C.W.), and contributed to editing and revisions (A.V., E.T., A.L., T.A.M., T.M., P.C.W.).

## Funding

This research was funded by the Academy of Finland (project numbers 324602, 329334, 326534 and 363740 to P.C.W., and 324605 to T.M.), by the University of Jyväskylä (to A.V.), and the Samuel Freeman Charitable Trust (to T.A.M.).

## Disclosure

Benefit‐sharing statement: This project is part of a continuing research collaboration with scientists from the Ukraine, who are involved in all stages of the project from design to implementation (e.g., conducting fieldwork, providing samples and laboratory work) and analyses. Our collaborators are included as principal‐ and co‐authors and the results of this research are shared among the research team and the broader scientific community (see Data Accessibility section). This research addresses a priority concern—understanding the biological consequences of exposure to radionuclides—with the development of field enclosures aimed at creating unique facilities to study the biological impacts of radionuclide exposure within the Chornobyl Exclusion Zone (CEZ).

## Ethics Statement

Experimental procedures complied with the legal requirements and international guidelines for the use of animals in research, with permission obtained from the Animal Experimentation Committee (ESAVI/7256/04.10.07/2014) and under the Permission for the use of natural resources 164/2021 issued on 22.09.2021 by the Ministry of Environmental Protection and Natural Resources (Ukraine).

## Conflicts of Interest

The authors declare no conflicts of interest.

## Supporting information


**Figure S1a:** Histogram of the number of reads per sample in samples of gut bacteria from bank voles ( *Clethrionomys glareolus* ) inhabiting contaminated and uncontaminated areas or field enclosures located within the Chornobyl Exclusion Zone (CEZ), Ukraine. Dashed line indicates the chosen even sampling depth (12,010 reads) as this retained 84% of samples and lost only 90 features.
**Figure S1b:** Rarefaction curves for samples of gut bacteria from bank voles ( *Clethrionomys glareolus* ) inhabiting contaminated and uncontaminated areas or field enclosures located within the Chornobyl Exclusion Zone (CEZ), Ukraine. Blue line indicates the chosen even sampling depth (12,010 reads).
**Figure S2:** Relative abundance of phyla, orders and families of gut bacteria from bank voles ( *Clethrionomys glareolus* ) inhabiting contaminated and uncontaminated areas (July) or field enclosures (August and October) located within the Chornobyl Exclusion Zone (CEZ), Ukraine. Corresponding data on taxonomic relative proportions are given in Table S7.
**Figure S3:** (a–c) Model diagnostic plots and tests of simulated residuals (output from the package *DHARMa* v.0.4.6 that examines whether there are substantial deviations from model assumptions by examining uniformity of residuals, over/under dispersion and occurrence of outliers). Diagnostic analyses completed for generalised linear mixed effects models (GLMMs) that examine predictors of variation in three measures of alpha diversity in gut bacteria from bank voles ( *Clethrionomys glareolus* ) inhabiting contaminated and uncontaminated areas or field enclosures located within the Chornobyl Exclusion Zone (CEZ), Ukraine. Model predictors were the fixed effects of Treatment × Time + sex + body weight and the random effects of capture location (or field enclosure) and animal identity. Measures of alpha diversity were (a) observed features, (b) Shannon index and (c) Faith's phylogenetic diversity.
**Figure S4:** (a–c) Model diagnostic plots and tests of simulated residuals (output from the package *DHARMa* v.0.4.6 that examines whether there are substantial deviations from model assumptions by examining uniformity of residuals, over/under dispersion and occurrence of outliers). Diagnostic analyses completed for generalised linear mixed effects models (GLMMs) that examine predictors of variation in three measures of alpha diversity in gut bacteria from bank voles ( *Clethrionomys glareolus* ) inhabiting contaminated and uncontaminated areas or field enclosures located within the Chornobyl Exclusion Zone (CEZ), Ukraine. Model predictors were the fixed effects of Treatment + Time, with the random effects of capture location (or enclosure) and animal identity. Measures of alpha diversity were (a) observed features, (b) Shannon index and (c) Faith's phylogenetic diversity.
**Figure S5:** (a–c) Temporal (during July, August and October) variation in three measures of beta diversity ((a) Bray Curtis index, (b) unweighted UniFrac metric and (c) weighted UniFrac metric) for gut bacteria of bank voles ( *Clethrionomys glareolus* ) inhabiting contaminated (brown) and uncontaminated (blue) areas (July) or field enclosures (August and October) located within the Chornobyl Exclusion Zone (CEZ), Ukraine. Individual samples and confidence ellipses are shown in separate, faceted panels by the level of radionuclide contamination for clarity, while group centroids are displayed in both panels to facilitate comparison of group‐level changes (see Table 1 for summary of PERMANOVA analyses). The principal coordinate analysis (PCoA) visualisation of Jaccard's dissimilarity for the same samples is provided in Figure 3. Statistical testing for a difference in mean (centroid) beta diversity among groups are given in Table 1 (main text) and in Table S4a,b.
**Figure S6:** Model diagnostic plots and tests of simulated residuals (output from the package *DHARMa* v.0.4.6 that examines whether there are substantial deviations from model assumptions by examining uniformity of residuals, over/under dispersion and occurrence of outliers). Diagnostic analyses completed for generalised linear mixed effects model (GLMM) that examine predictors of variation in the ratio of the phyla of gut bacteria Firmicutes (= Bacillota) and Bacteroidetes (= Bacteroidota) (F:B ratio calculated as centre log ratio, CLR) from bank voles ( *Clethrionomys glareolus* ) inhabiting contaminated and uncontaminated areas (July) or field enclosures (August and October) located within the Chornobyl Exclusion Zone (CEZ), Ukraine. Model predictors were the fixed effects of Treatment + Time, with the random effects of capture location (or enclosure) and animal identity. See Table 2 for model results.
**Figure S7:** Longitudinal changes (first distances) in four metrics of beta diversity for gut bacteria of bank voles ( *Clethrionomys glareolus* ) inhabiting contaminated (brown) and uncontaminated (blue) areas and field enclosures located within the Chornobyl Exclusion Zone (CEZ), Ukraine.
**Figure S8:** (a–d) Model diagnostic plots and tests of simulated residuals (output from the package *DHARMa* v.0.4.6 that examines whether there are substantial deviations from model assumptions by examining uniformity of residuals, over/under dispersion and occurrence of outliers). Diagnostic analyses completed for generalised linear mixed effects models (GLMMs) that examine predictors of variation in mean temporal change in beta diversity (for four measures of beta diversity) in gut bacteria from bank voles ( *Clethrionomys glareolus* ) inhabiting contaminated and uncontaminated areas or field enclosures located within the Chornobyl Exclusion Zone (CEZ), Ukraine. Model predictors were the fixed effects of Treatment and Time interval and their interaction, with the random effects of capture location (or enclosure) and animal identity. Measures of beta diversity were (a) Jaccard's index, (b) Bray‐Curtis index, (c) UniFrac metric and (d) weighted UniFrac metric.
**Figure S9:** (a–d) Model diagnostic plots and tests of simulated residuals (output from the package *DHARMa* v.0.4.6 that examines whether there are substantial deviations from model assumptions by examining uniformity of residuals, over/under dispersion and occurrence of outliers). Diagnostic analyses completed for generalised linear mixed effects models (GLMMs) that examine predictors of variation in the dispersion of temporal changes in beta diversity, quantified as the absolute deviation of pairwise temporal distances from their group mean, in gut bacteria from bank voles ( *Clethrionomys glareolus* ) inhabiting contaminated and uncontaminated areas or field enclosures located within the Chornobyl Exclusion Zone (CEZ), Ukraine. Model predictors were the fixed effects of Treatment and Time interval and their interaction, with the random effects of capture location (or enclosure) and animal identity. Measures of beta diversity were (a) Jaccard's index, (b) Bray‐Curtis index, (c) UniFrac metric and (d) weighted UniFrac metric.
**Table S1:** Sample sizes and summary statistics for absorbed doses of radiation experienced by bank voles ( *Clethrionomys glareolus* ) inhabiting contaminated and uncontaminated areas (July) or field enclosures (August and October) located within the Chornobyl Exclusion Zone (CEZ), Ukraine. IQR, interquartile range; *n*, sample size.
**Table S2:** Statistical significance of pairwise tests (Dunn's pairwise comparisons with Benjamini‐Hochberg correction for multiple testing) for differences in the absorbed dose of radiation experienced by bank voles ( *Clethrionomys glareolus* ) inhabiting contaminated and uncontaminated areas (July) or field enclosures (August and October) located within the Chornobyl Exclusion Zone (CEZ), Ukraine. *p*, statistical significance; *p.adj*, adjusted statistical significance; *Z*, test statistic.
**Table S3a:** Likelihood ratio test results for fixed effects in generalised linear mixed‐effects models (GLMMs) examining predictors of variation in three measures of alpha diversity in the gut microbiota of bank voles ( *Clethrionomys glareolus* ) from contaminated and uncontaminated areas in the Chornobyl Exclusion Zone (CEZ), Ukraine. (a) Observed features, (b) Shannon index and (c) Faith's phylogenetic diversity. Chisq., chi‐square statistic; df, degrees of freedom; *p*, statistical significance.
**Table S3b:** Statistical significance of generalised linear mixed effects models (GLMMs) (with an interaction between Treatment and Time) that examine predictors of variation in three measures of alpha diversity in gut bacteria of bank voles ( *Clethrionomys glareolus* ) inhabiting contaminated and uncontaminated areas or field enclosures located within the Chornobyl Exclusion Zone (CEZ), Ukraine. (a) Observed features, (b) Shannon index and (c) Faith's phylogenetic diversity. CI lower/CI upper, lower and upper confidence intervals; Est., estimate; *p*, statistical significance; SE, standard error.
**Table S3c:** Statistical significance of generalised linear mixed effects models (GLMMs) that examine predictors (only the main effects of Treatment and Time) of variation in three measures of alpha diversity in gut bacteria of bank voles ( *Clethrionomys glareolus* ) inhabiting contaminated and uncontaminated areas or field enclosures located within the Chornobyl Exclusion Zone (CEZ), Ukraine. (a) Observed features, (b) Shannon index and (c) Faith's phylogenetic diversity. CI lower/CI upper, lower and upper confidence intervals; Est., estimate; *p*, statistical significance; SE, standard error.
**Table S4a:** Statistical significance (by PERMANOVA) and effect sizes of exposure to environmental radionuclides (Treatment = contaminated or uncontaminated areas) or temporal changes (Time = sampling in July, August, or October) on variation in beta diversity of gut bacteria from the bank vole ( *Clethrionomys glareolus* ) inhabiting contaminated and uncontaminated areas (July) or field enclosures (August and October) located within the Chornobyl Exclusion Zone (CEZ), Ukraine. Models were run with (Treatment × Time) and without (Treatment + Time) an interaction between Treatment and Time. df, degrees of freedom; *F*, test statistic; *MS*, mean square; *p*, statistical significance; *R* ^*2*^, effect size; *SS*, sum of squares. PCoA visualisations of beta diversity are given in Figure 3 main text (Jaccard's metric) and in Figure S5a–c (all other metrics).
**Table S4b:** Statistical significance (by PERMANOVA) and effect sizes of exposure to environmental radionuclides (treatment = total absorbed dose in mGy) or temporal changes (time = three sampling times—July, August, or October) on variation in beta diversity of gut bacteria from the bank vole ( *Clethrionomys glareolus* ) inhabiting contaminated and uncontaminated areas (July) or field enclosures (August and October) located within the Chornobyl Exclusion Zone (CEZ), Ukraine. df, degrees of freedom; *F*, test statistic; *MS*, mean square; *p*, statistical significance; *R* ^*2*^, effect size; *SS*, sum of squares. PCoA visualisations of beta diversity are given in Figure 3 main text (Jaccard's metric) and in Figure S5a–c (all other metrics).
**Table S5a:** Statistical significance of differences in dispersion in beta diversity of gut bacteria from the bank vole ( *Clethrionomys glareolus* ) inhabiting contaminated and uncontaminated areas (July) or field enclosures (August and October) located within the Chornobyl Exclusion Zone (CEZ), Ukraine. df, degrees of freedom; *F*, test statistic; *MS*, mean square; *p*, statistical significance; *SS*, sum of squares. PCoA visualisations of beta diversity are given in Figure 3 (Jaccard's dissimilarity) and Figure S2a–c (all other metrics).
**Table S5b:** Statistical significance of differences in dispersion in beta diversity of gut bacteria from the bank vole ( *Clethrionomys glareolus* ) inhabiting contaminated and uncontaminated areas (July) or field enclosures (August and October) located within the Chornobyl Exclusion Zone (CEZ), Ukraine. df, degrees of freedom; *F*, test statistic; *MS*, mean square; *p*, statistical significance; *SS*, sum of squares. PCoA visualisations of beta diversity are given in Figure 3 (Jaccard's dissimilarity) and Figure S2a–c (all other metrics).
**Table S6a:** Outcomes from generalised linear mixed models (GLMMs) testing the effects of Treatment (uncontaminated or contaminated habitat) and Time interval (July‐Augus and Augst‐October) on mean temporal changes in beta diversity of gut bacteria of bank voles ( *Clethrionomys glareolus* ) inhabiting contaminated and uncontaminated areas and field enclosures located within the Chornobyl Exclusion Zone (CEZ), Ukraine. Models were fitted for each metric of beta diversity, with Treatment, Time interval and their interaction as fixed effects (and individual identity and sample location as random effects). (a) Jaccard's metric, (b) Bray‐Curtis index, (c) UniFrac metric and (d) weighted UniFrac metric. CI lower/CI upper, lower and upper confidence intervals; Est., estimate; *p*, statistical significance; SE, standard error.
**Table S6b:** Outcomes from generalised linear mixed models (GLMMs) testing the effects of Treatment (uncontaminated or contaminated habitat) and Time interval (July‐August and August‐October) on the dispersion of temporal changes (deviation of observations from the group mean) in beta diversity of gut bacteria of bank voles ( *Clethrionomys glareolus* ) inhabiting contaminated and uncontaminated areas and field enclosures located within the Chornobyl Exclusion Zone (CEZ), Ukraine. Models were fitted for each metric of beta diversity, with Treatment, Time interval and their interaction as fixed effects (and individual identity and sample location as random effects). (a) Jaccard's metric, (b) Bray‐Curtis index, (c) UniFrac metric and (d) weighted UniFrac metric. CI lower/CI upper, lower and upper confidence intervals; Est., estimate; *p*, statistical significance; SE, standard error.
**Table S7:** Relative abundance of the dominant phyla, orders and families of gut bacteria from bank voles ( *Clethrionomys glareolus* ) inhabiting contaminated and uncontaminated areas (July) or field enclosures (August and October) located within the Chornobyl Exclusion Zone (CEZ), Ukraine.
**Table S8:** Results of ANCOM‐BC2 analysis showing estimated coefficients for differential abundance of gut bacterial taxa in relation to radiation dose and time from bank voles ( *Clethrionomys glareolus* ) inhabiting contaminated and uncontaminated areas located within the Chornobyl Exclusion Zone (CEZ), Ukraine. *lfc*, log fold change (mGy^−1^); *q*, statistical significance adjusted for multiple testing; SE, standard error of lfc; *W*, test statistic.

## Data Availability

Raw sequence reads are deposited in the SRA (BioProject ID PRJNA1304226) with the metadata stored under the same BioProject using the Package MIMARKS: survey, host‐associated v.6.0. R code to analyse data and prepare figures are available at https://github.com/phillipwattsJYU/CEZ_enclosures/.
