# Supplementary material for: Timing Is Everything: The Effect of Exposure to Pollution on Wildlife Gut Microbiota Is Contingent on Season
Source: Mol Ecol. 2026 Apr 22;35:e70346. doi: 10.1111/mec.70346 (PMC13100865; doi:10.1111/mec.70346)
Supplement: Supplementary file 1 — Figure S1a: Histogram of the number of reads per sample in samples of gut bacteria from bank voles ( Clethrionomys glareolus ) inhabiting contaminated and uncontaminated areas or field enclosures located within the Chornobyl Exclusion Zone (CEZ), Ukraine. Dashed line indicates the chosen even sampling depth (12,010 reads) as this retained 84% of samples and lost only 90 features. Figure S1b: Rarefaction curves for samples of gut bacteria from bank voles ( Clethrionomys glareolus ) inhabiting contaminated and uncontaminated areas or field enclosures located within the Chornobyl Exclusion Zone (CEZ), Ukraine. Blue line indicates the chosen even sampling depth (12,010 reads). Figure S2: Relative abundance of phyla, orders and families of gut bacteria from bank voles ( Clethrionomys glareolus ) inhabiting contaminated and uncontaminated areas (July) or field enclosures (August and October) located within the Chornobyl Exclusion Zone (CEZ), Ukraine. Corresponding data on taxonomic relative proportions are given in Table S7. Figure S3: (a–c) Model diagnostic plots and tests of simulated residuals (output from the package DHARMa v.0.4.6 that examines whether there are substantial deviations from model assumptions by examining uniformity of residuals, over/under dispersion and occurrence of outliers). Diagnostic analyses completed for generalised linear mixed effects models (GLMMs) that examine predictors of variation in three measures of alpha diversity in gut bacteria from bank voles ( Clethrionomys glareolus ) inhabiting contaminated and uncontaminated areas or field enclosures located within the Chornobyl Exclusion Zone (CEZ), Ukraine. Model predictors were the fixed effects of Treatment × Time + sex + body weight and the random effects of capture location (or field enclosure) and animal identity. Measures of alpha diversity were (a) observed features, (b) Shannon index and (c) Faith's phylogenetic diversity. Figure S4: (a–c) Model diagnostic plots and tests of simulate [file MEC-35-e70346-s001.docx]

**Supplemental Information for:**

**Timing is everything: the effects of exposure to pollution on wildlife gut microbiota is contingent on season.**

Andrii Vasylenko^1^, Eugene Tukalenko^1,2^, Anton Lavrinienko^1,3^, Timothy A. Mousseau^4^, Tapio Mappes^1^, Phillip C. Watts^1^

^1^Department of Biological and Environmental Science, University of Jyväskylä, Finland

^2^Department of Radiobiology and Radioecology, Institute for Nuclear Research of the National Academy of Sciences of Ukraine, Kyiv, Ukraine

^3^Department of Health Sciences and Technology, ETH Zurich, Zürich, Switzerland

^4^Department of Biological Sciences, University of South Carolina, Columbia, USA

**Table of Contents:**

| **Supplementary Methods** | Page 5 |
| --- | --- |
| **Supplementary Figure 1a**. Histogram of the number of reads per sample in samples of gut bacteria from bank voles (*Clethrionomys glareolus*) inhabiting contaminated and uncontaminated areas or field enclosures located within the Chornobyl Exclusion Zone (CEZ), Ukraine. Dashed line indicates the chosen even sampling depth (=12,010 reads) as this retained 84% of samples and lost only 90 features. | Page 6 |
| **Supplementary Figure 1b**. Rarefaction curves for samples of gut bacteria from bank voles (*Clethrionomys glareolus*) inhabiting contaminated and uncontaminated areas or field enclosures located within the Chornobyl Exclusion Zone (CEZ), Ukraine. Blue line indicates the chosen even sampling depth (=12,010 reads). | Page 7 |
| **Supplementary Figure 2.** Relative abundance of phyla, orders, and families of gut bacteria from bank voles (*Clethrionomys glareolus*) inhabiting contaminated and uncontaminated areas (July) or field enclosures (August and October) located within the Chornobyl Exclusion Zone (CEZ), Ukraine. Corresponding data on taxonomic relative proportions are given in Supplementary Table 7. | Page 8 |
| **Supplementary Figure 3a, b, c.** Model diagnostic plots and tests of simulated residuals (output from the package *DHARMa* v.0.4.6 that examines whether there are substantial deviations from model assumptions by examining uniformity of residuals, over/under dispersion, and occurrence of outliers). Diagnostic analyses completed for generalised linear mixed effects models (GLMMs) that examine predictors of variation in three measures of alpha diversity in gut bacteria from bank voles (*Clethrionomys glareolus*) inhabiting contaminated and uncontaminated areas or field enclosures located within the Chornobyl Exclusion Zone (CEZ), Ukraine. Model predictors were the fixed effects of Treatment * Time + sex + body weight and the random effects of capture location (or field enclosure) and animal identity. Measures of alpha diversity were (a) observed features, (b) Shannon index, and (c) Faith’s phylogenetic diversity. | Page 9-10 |
| **Supplementary Figure 4a, b, c.** Model diagnostic plots and tests of simulated residuals (output from the package *DHARMa* v.0.4.6 that examines whether there are substantial deviations from model assumptions by examining uniformity of residuals, over/under dispersion, and occurrence of outliers). Diagnostic analyses completed for generalised linear mixed effects models (GLMMs) that examine predictors of variation in three measures of alpha diversity in gut bacteria from bank voles (*Clethrionomys glareolus*) inhabiting contaminated and uncontaminated areas or field enclosures located within the Chornobyl Exclusion Zone (CEZ), Ukraine. Model predictors were the fixed effects of Treatment + Time, with the random effects of capture location (or enclosure) and animal identity. Measures of alpha diversity were (a) observed features, (b) Shannon index, and (c) Faith’s phylogenetic diversity. | Page 11-12 |
| **Supplementary Figures 5a, b, c**. Temporal (during July, August, and October) variation in three measures of beta diversity ((a) Bray Curtis index, (b) unweighted UniFrac metric, and (c) weighted UniFrac metric) for gut bacteria of bank voles (*Clethrionomys glareolus*) inhabiting contaminated (brown) and uncontaminated (blue) areas (July) or field enclosures (August and October) located within the Chornobyl Exclusion Zone (CEZ), Ukraine. Individual samples and confidence ellipses are shown in separate, faceted panels by the level of radionuclide contamination for clarity, while group centroids are displayed in both panels to facilitate comparison of group-level changes (see Table 1 for summary of PERMANOVA analyses). The principal coordinate analysis (PCoA) visualisation of Jaccard’s dissimilarity for the same samples is provided in Figure 3. Statistical testing for a difference in mean (centroid) beta diversity among groups are given in Table 1 (main text) and in Supplementary Tables 4a, b. | Page 13-14 |
| **Supplementary Figure 6**. Model diagnostic plots and tests of simulated residuals (output from the package *DHARMa* v.0.4.6 that examines whether there are substantial deviations from model assumptions by examining uniformity of residuals, over/under dispersion, and occurrence of outliers). Diagnostic analyses completed for generalised linear mixed effects model (GLMM) that examine predictors of variation in the ratio of the phyla of gut bacteria Firmicutes (=Bacillota) and Bacteroidetes (=Bacteroidota) (F:B ratio calculated as centre log ratio, CLR) from bank voles (*Clethrionomys glareolus*) inhabiting contaminated and uncontaminated areas (July) or field enclosures (August and October) located within the Chornobyl Exclusion Zone (CEZ), Ukraine. Model predictors were the fixed effects of Treatment + Time, with the random effects of capture location (or enclosure) and animal identity. See Table 2 for model results. | Page 15 |
| **Supplementary Figure 7**. Longitudinal changes (first distances) in four metrics of beta diversity for gut bacteria of bank voles (*Clethrionomys glareolus*) inhabiting contaminated (brown) and uncontaminated (blue) areas and field enclosures located within the Chornobyl Exclusion Zone (CEZ), Ukraine. | Page 16 |
| **Supplementary Figures 8a, b, c, d**. Model diagnostic plots and tests of simulated residuals (output from the package *DHARMa* v.0.4.6 that examines whether there are substantial deviations from model assumptions by examining uniformity of residuals, over/under dispersion, and occurrence of outliers). Diagnostic analyses completed for generalised linear mixed effects models (GLMMs) that examine predictors of variation in mean temporal change in beta diversity (for four measures of beta diversity) in gut bacteria from bank voles (*Clethrionomys glareolus*) inhabiting contaminated and uncontaminated areas or field enclosures located within the Chornobyl Exclusion Zone (CEZ), Ukraine. Model predictors were the fixed effects of Treatment and Time interval and their interaction, with the random effects of capture location (or enclosure) and animal identity. Measures of beta diversity were (a) Jaccard’s index, (b) Bray-Curtis index, (c) UniFrac metric, and (d) weighted UniFrac metric. | Page 17-19 |
| **Supplementary Figures 9a, b, c, d**. Model diagnostic plots and tests of simulated residuals (output from the package *DHARMa* v.0.4.6 that examines whether there are substantial deviations from model assumptions by examining uniformity of residuals, over/under dispersion, and occurrence of outliers). Diagnostic analyses completed for generalised linear mixed effects models (GLMMs) that examine predictors of variation in the dispersion of temporal changes in beta diversity, quantified as the absolute deviation of pairwise temporal distances from their group mean, in gut bacteria from bank voles (*Clethrionomys glareolus*) inhabiting contaminated and uncontaminated areas or field enclosures located within the Chornobyl Exclusion Zone (CEZ), Ukraine. Model predictors were the fixed effects of Treatment and Time interval and their interaction, with the random effects of capture location (or enclosure) and animal identity. Measures of beta diversity were (a) Jaccard’s index, (b) Bray-Curtis index, (c) UniFrac metric, and (d) weighted UniFrac metric. | Page 20-22 |
| **Supplementary Table 1**. Sample sizes and summary statistics for absorbed doses of radiation experienced by bank voles (*Clethrionomys glareolus*) inhabiting contaminated and uncontaminated areas (July) or field enclosures (August and October) located within the Chornobyl Exclusion Zone (CEZ), Ukraine. *n*, sample size; *IQR*, interquartile range. | Page 23 |
| **Supplementary Table 2**. Statistical significance of pairwise tests (Dunn’s pairwise comparisons with Benjamini-Hochberg correction for multiple testing) for differences in the absorbed dose of radiation experienced by bank voles (*Clethrionomys glareolus*) inhabiting contaminated and uncontaminated areas (July) or field enclosures (August and October) located within the Chornobyl Exclusion Zone (CEZ), Ukraine. *Z*, test statistic; *p*, statistical significance; *p.adj*, adjusted statistical significance). | Page 24 |
| **Supplementary Table 3a**. Likelihood ratio test results for fixed effects in generalized linear mixed-effects models (GLMMs) examining predictors of variation in three measures of alpha diversity in the gut microbiota of bank voles (*Clethrionomys glareolus*) from contaminated and uncontaminated areas in the Chornobyl Exclusion Zone (CEZ), Ukraine. (a) observed features, (b) Shannon index, and (c) Faith’s phylogenetic diversity. Chisq., chi-square statistic; df, degrees of freedom; p, statistical significance. | Page 25 |
| **Supplementary Table 3b**. Statistical significance of generalised linear mixed effects models (GLMMs) (with an interaction between Treatment and Time) that examine predictors of variation in three measures of alpha diversity in gut bacteria of bank voles (*Clethrionomys glareolus*) inhabiting contaminated and uncontaminated areas or field enclosures located within the Chornobyl Exclusion Zone (CEZ), Ukraine. (a) observed features, (b) Shannon index, and (c) Faith’s phylogenetic diversity. Est., estimate; SE, standard Error; CI lower/CI upper, lower and upper confidence intervals; *p*, statistical significance. | Page 26 |
| **Supplementary Table 3c**. Statistical significance of generalised linear mixed effects models (GLMMs) that examine predictors (only the main effects of Treatment and Time) of variation in three measures of alpha diversity in gut bacteria of bank voles (*Clethrionomys glareolus*) inhabiting contaminated and uncontaminated areas or field enclosures located within the Chornobyl Exclusion Zone (CEZ), Ukraine. (a) observed features, (b) Shannon index, and (c) Faith’s phylogenetic diversity. Est., estimate; SE, standard Error; CI lower/CI upper, lower and upper confidence intervals; *p*, statistical significance. | Page 27 |
| **Supplementary Table 4a**. Statistical significance (by PERMANOVA) and effect sizes of exposure to environmental radionuclides (Treatment=contaminated or uncontaminated areas) or temporal changes (Time=sampling in July, August, or October) on variation in beta diversity of gut bacteria from the bank vole (*Clethrionomys glareolus*) inhabiting contaminated and uncontaminated areas (July) or field enclosures (August and October) located within the Chornobyl Exclusion Zone (CEZ), Ukraine. Models were run with (Treatment*Time) and without (Treatment+Time) an interaction between Treatment and Time. *df*, degrees of freedom; *SS*, sum of squares; *MS* mean square; *R^2^*, effect size; *F*, test statistic; *p*, statistical significance. PCoA visualisations of beta diversity are given in Figure 3 main text (Jaccard’s metric) and in Supplementary Figures 5a, b, c (all other metrics). | Page 28 |
| **Supplementary Table 4b.** Statistical significance (by PERMANOVA) and effect sizes of exposure to environmental radionuclides (treatment=total absorbed dose in mGy) or temporal changes (time=three sampling times - July, August, or October) on variation in beta diversity of gut bacteria from the bank vole (*Clethrionomys glareolus*) inhabiting contaminated and uncontaminated areas (July) or field enclosures (August and October) located within the Chornobyl Exclusion Zone (CEZ), Ukraine. *df*, degrees of freedom; *SS*, sum of squares; *MS* mean square; *R^2^* , effect size; *F*, test statistic; *p*, statistical significance. PCoA visualisations of beta diversity are given in Figure 3 main text (Jaccard’s metric) and in Supplementary Figures 5a, b, c (all other metrics). | Page 29 |
| **Supplementary Table 5a**. Statistical significance of differences in dispersion in beta diversity of gut bacteria from the bank vole (*Clethrionomys glareolus*) inhabiting contaminated and uncontaminated areas (July) or field enclosures (August and October) located within the Chornobyl Exclusion Zone (CEZ), Ukraine. *df*, degrees of freedom; *SS*, sum of squares; *MS* mean square; *F*, test statistic; *p*, statistical significance. PCoA visualisations of beta diversity are given in Figure 3 (Jaccard’s dissimilarity) and Supplementary Figures 2a, b, c (all other metrics). | Page 30 |
| **Supplementary Table 5b**. Statistical significance of differences in dispersion in beta diversity of gut bacteria from the bank vole (*Clethrionomys glareolus*) inhabiting contaminated and uncontaminated areas (July) or field enclosures (August and October) located within the Chornobyl Exclusion Zone (CEZ), Ukraine. *df*, degrees of freedom; *SS*, sum of squares; *MS* mean square; *F*, test statistic; *p*, statistical significance. PCoA visualisations of beta diversity are given in Figure 3 (Jaccard’s dissimilarity) and Supplementary Figures 2a, b, c (all other metrics). | Page 31 |
| **Supplementary Table 6a.** Outcomes from generalised linear mixed models (GLMMs) testing the effects of Treatment (uncontaminated or contaminated habitat) and Time interval (July-Augus and Augst-October) on mean temporal changes in beta diversity of gut bacteria of bank voles (*Clethrionomys glareolus*) inhabiting contaminated and uncontaminated areas and field enclosures located within the Chornobyl Exclusion Zone (CEZ), Ukraine. Models were fitted for each metric of beta diversity, with Treatment, Time interval, and their interaction as fixed effects (and individual identity and sample location as random effects). (a) Jaccard’s metric, (b) Bray-Curtis index, (c) UniFrac metric, and (d) weighted UniFrac metric. Est., estimate; SE, standard Error; CI lower/CI upper, lower and upper confidence intervals; *p*, statistical significance. | Page 32 |
| **Supplementary Table 6b.** Outcomes from generalised linear mixed models (GLMMs) testing the effects of Treatment (uncontaminated or contaminated habitat) and Time interval (July-Augus and Augst-October) on the dispersion of temporal changes (deviation of observations from the group mean) in beta diversity of gut bacteria of bank voles (*Clethrionomys glareolus*) inhabiting contaminated and uncontaminated areas and field enclosures located within the Chornobyl Exclusion Zone (CEZ), Ukraine. Models were fitted for each metric of beta diversity, with Treatment, Time interval, and their interaction as fixed effects (and individual identity and sample location as random effects). (a) Jaccard’s metric, (b) Bray-Curtis index, (c) UniFrac metric, and (d) weighted UniFrac metric. Est., estimate; SE, standard Error; CI lower/CI upper, lower and upper confidence intervals; *p*, statistical significance. | Page 33 |
| **Supplementary Table 7**. Relative abundance of the dominant phyla, orders, and families of gut bacteria from bank voles (*Clethrionomys glareolus*) inhabiting contaminated and uncontaminated areas (July) or field enclosures (August and October) located within the Chornobyl Exclusion Zone (CEZ), Ukraine. | Page 34 |
| **Supplementary Table 8**. Results of ANCOM-BC2 analysis showing estimated coefficients for differential abundance of gut bacterial taxa in relation to radiation dose and time from bank voles (*Clethrionomys glareolus*) inhabiting contaminated and uncontaminated areas located within the Chornobyl Exclusion Zone (CEZ), Ukraine. *lfc*, log fold change (mGy^-1^); *se*, standard error of lfc; *W*, test statistic; *q*, statistical significance adjusted for multiple testing. | Page 35-40 |

**Supplementary Methods**

*Dosimetry*

For each measurement of caesium–137 activity, the critical detectable level (*Lc*, decision threshold) was estimated as *Lc=k[Rb/Tb(1+Tb/Ts]^1/2^*, where *k* is 1.65 (coefficient, which determines a 0.05 probability of type I error), *Rb* is the background rate of radiation, *Tb* is time of background measurement, *Ts* is time of sample measurement (International Standard ISO 11929-3, Isaev et al., 2010). Caesium activities above the critical level (decision threshold) were used for internal dose rate estimation, otherwise the caesium activity was treated as a zero.

Individual internal absorbed doses from the incorporated ^137^Cs acquired during one day were calculated (mGy day^-1^) as a product of the activity of ^137^Cs incorporated in the animal body (Bq kg^-1^) and the unit conversion coefficient and the sum of all electron, positron, and photon energies emitted per decay of ^137^Cs and it daughter radionuclide ^137m^Ba (Cristy et al., 1987; Baltas et al., 2006). Thus, energies were calculated while taking account for the absorbed fractions for electron or positron or photon of the specific energy line, the intensity (or emission frequency) of the specific energy line (MeV) emitted per decay of ^137^Cs and its daughter radionuclide ^137m^ Ba (ICRP Publication 38, 1983), the assumption that ^137^Cs source is uniformly distributed throughout homogeneous sphere of 20 g mass of unit density and tissue-equivalent composition (Stabin et al., 2000). Despite the potentially significant contribution of ^90^Sr to the total internal absorbed dose, approximately the same as from ^137^Cs in Chornobyl (Beresford et al., 2020), we neglected the contribution of ^90^Sr because it is a beta-emitting radionuclide that is predominantly deposited (and thus acts) in bone tissue. Energies from alpha-emitters (radioisotopes of transuranium elements, plutonium, and americium) were also omitted because of their low contribution (<5 %) to total internal absorbed doses in animals (Beresford et al., 2020).

*References*

Baltas D, Sakelliou L, Zamboglou N. (2006) The Physics of Modern Brachytherapy for Oncology. Series in Medical Physics and Biomedical Engineering. CRC Press.

Beresford NA, Barnett CL, Gashchak S, Maksimenko A, Guliaichenko E, Wood MD, Izquierdo M. Radionuclide transfer to wildlife at a ‘Reference site’ in the Chernobyl Exclusion Zone and resultant radiation exposures (2020) J Environ Radioact, 211: https://doi.org/10.1016/j.jenvrad.2018.02.007.

Cristy M, Eckerman KF. (1987) Specific absorbed fraction of energy at various ages from internal photon sources. Oak Ridge, TN: Oak RidgeNational Laboratory; ReportORNL/TM-8381/V1-7.

Isaev A.G., Babenko V.V., Kazimirov A.S., Grishin S.N., Ievlev S.M. (2010) Minimum detectable activity. Basic concepts and definitions. Problemy bezpeky atomnykh elektrostancij i Chornobylja — The problem of the safety of nuclear power plants and Chernobyl), 13: 103—110 [In Russian].

Stabin M G and Konijnenberg MW (2000) Re-evaluation of absorbed fractions for photons and electrons in spheres of various sizes J. Nucl. Med. 41 149–60.


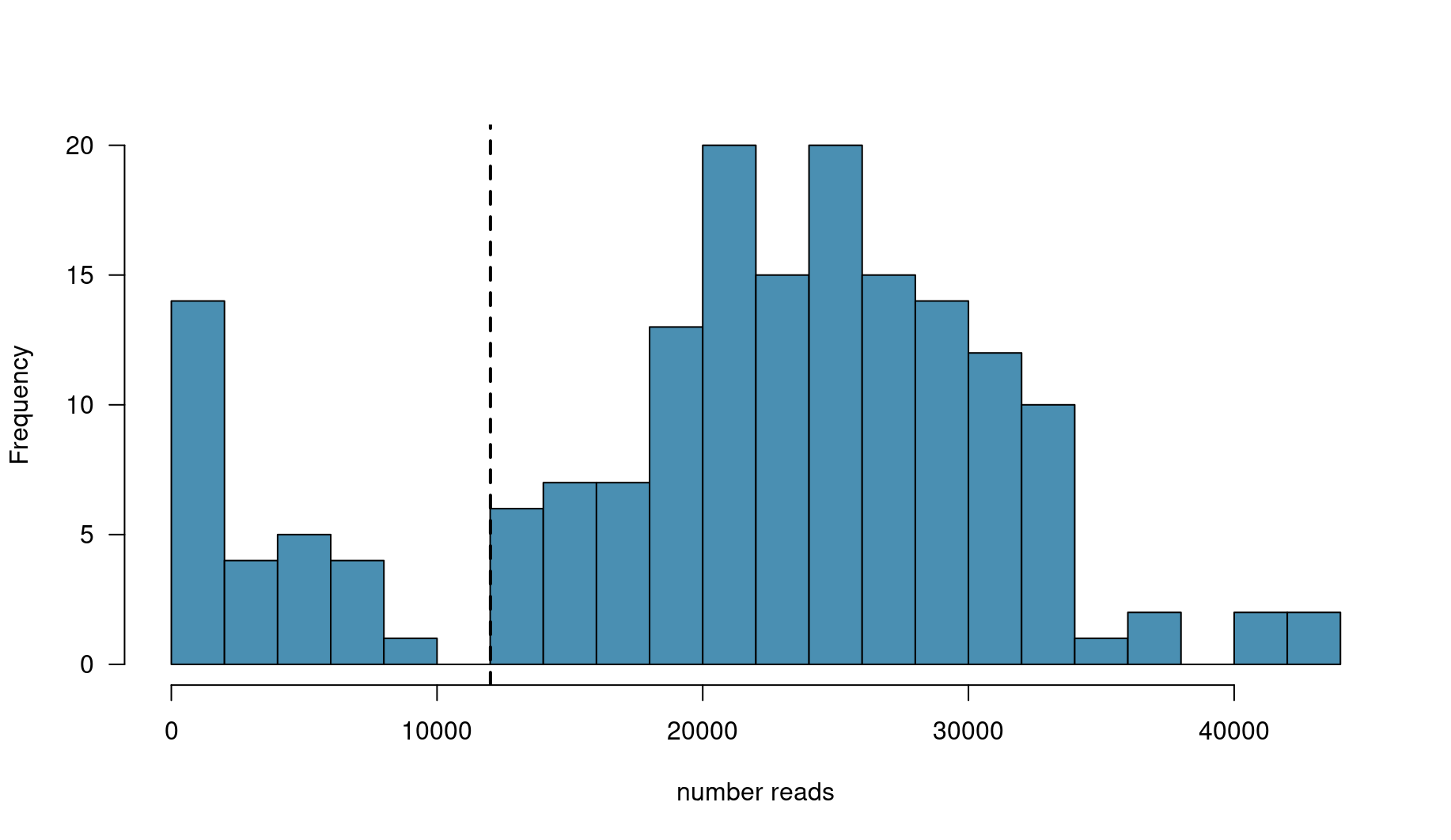


Supplementary Figure 1a. Histogram of the number of reads per sample in samples of gut bacteria from bank voles (*Clethrionomys glareolus*) inhabiting contaminated and uncontaminated areas or field enclosures located within the Chornobyl Exclusion Zone (CEZ), Ukraine. Dashed line indicates the chosen even sampling depth (=12,010 reads) as this retained 84% of samples and lost only 90 features.


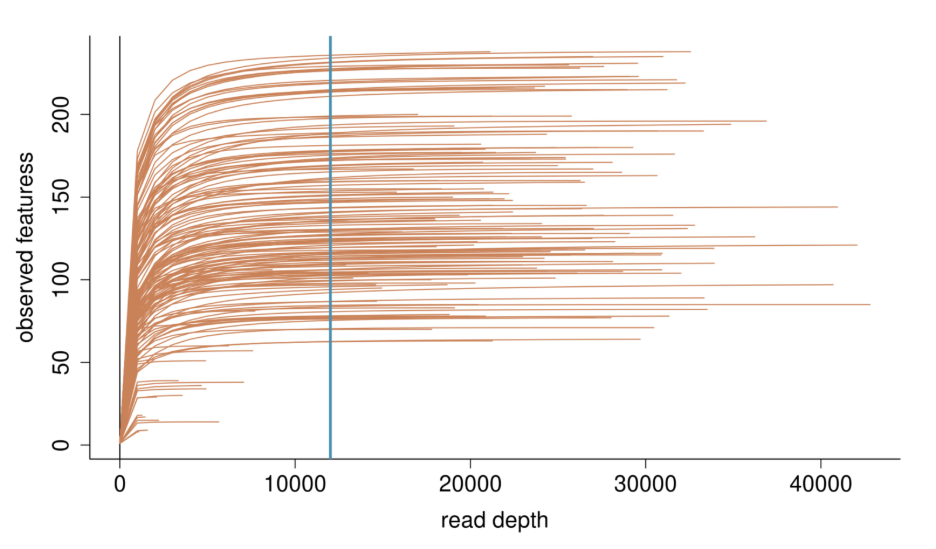


Supplementary Figure 1b. Rarefaction curves for samples of gut bacteria from bank voles (*Clethrionomys glareolus*) inhabiting contaminated and uncontaminated areas or field enclosures located within the Chornobyl Exclusion Zone (CEZ), Ukraine. Blue line indicates the chosen even sampling depth (=12,010 reads).


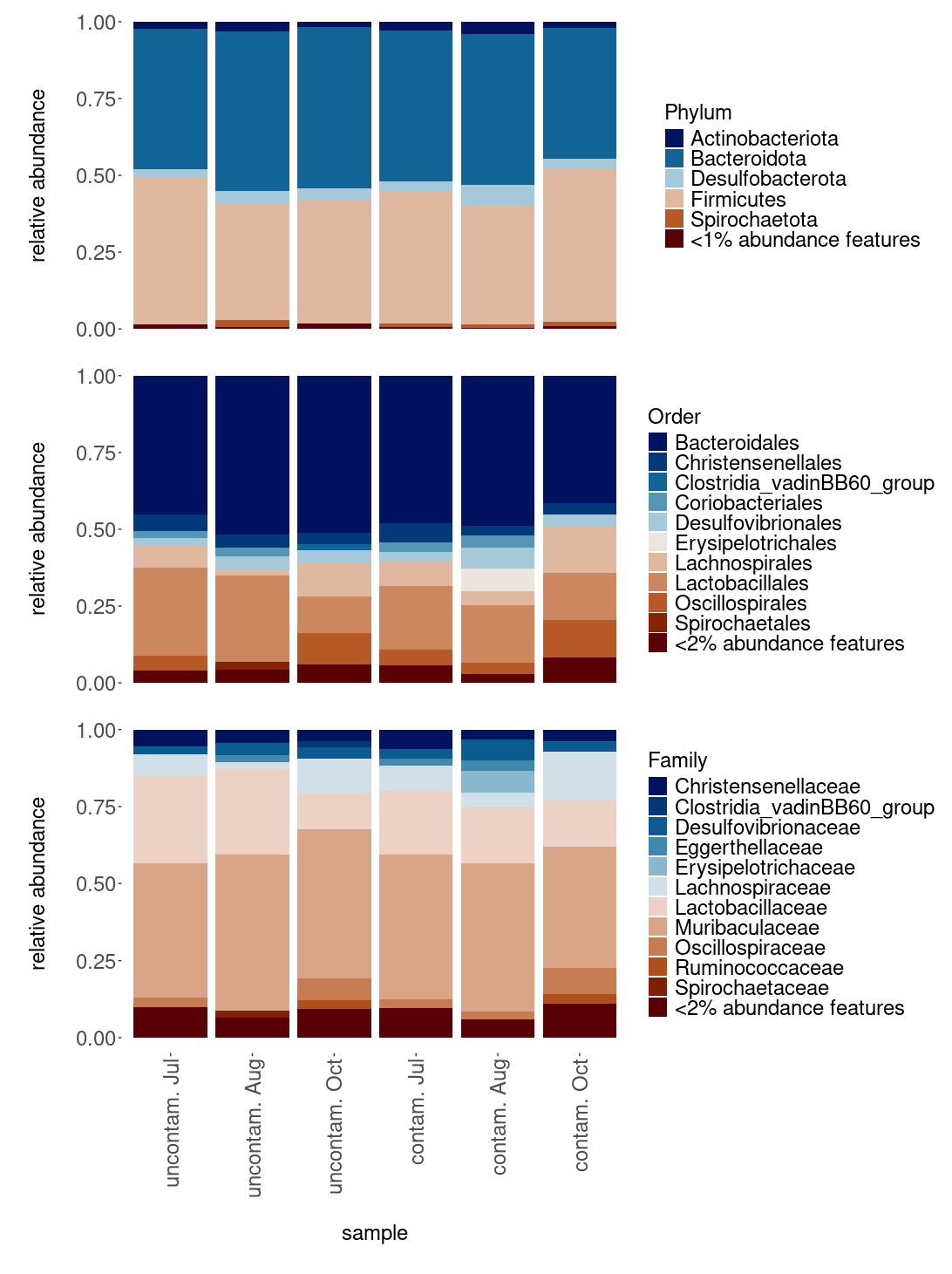


Supplementary Figure 2. Relative abundance of phyla, orders, and families of gut bacteria from bank voles (*Clethrionomys glareolus*) inhabiting contaminated and uncontaminated areas (July) or field enclosures (August and October) located within the Chornobyl Exclusion Zone (CEZ), Ukraine. Corresponding data on taxonomic relative proportions are given in Supplementary Table 7.

1. Observed features


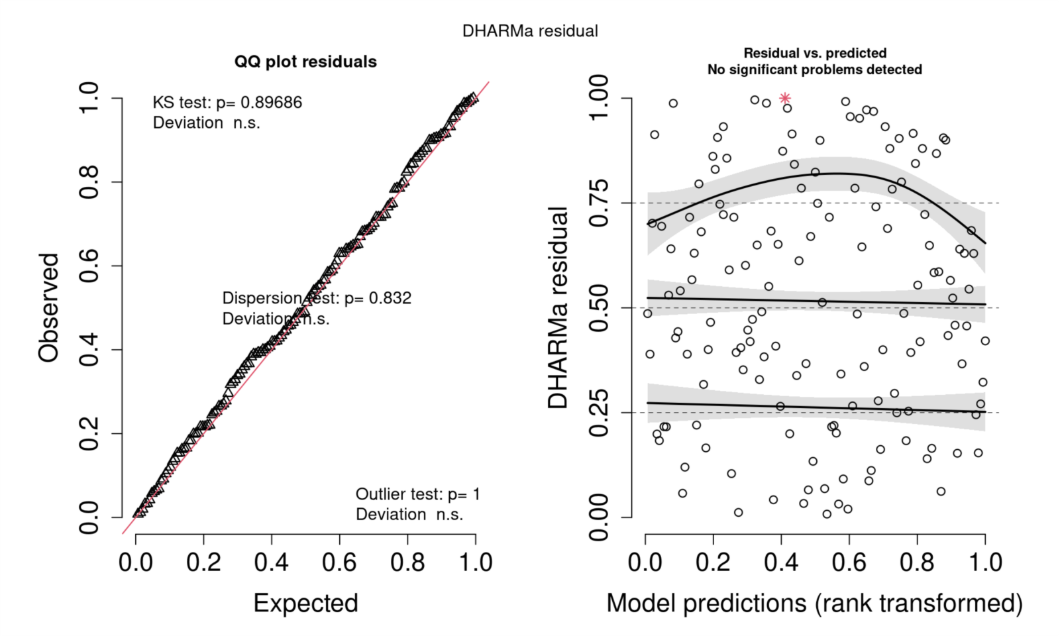


1. Shannon index


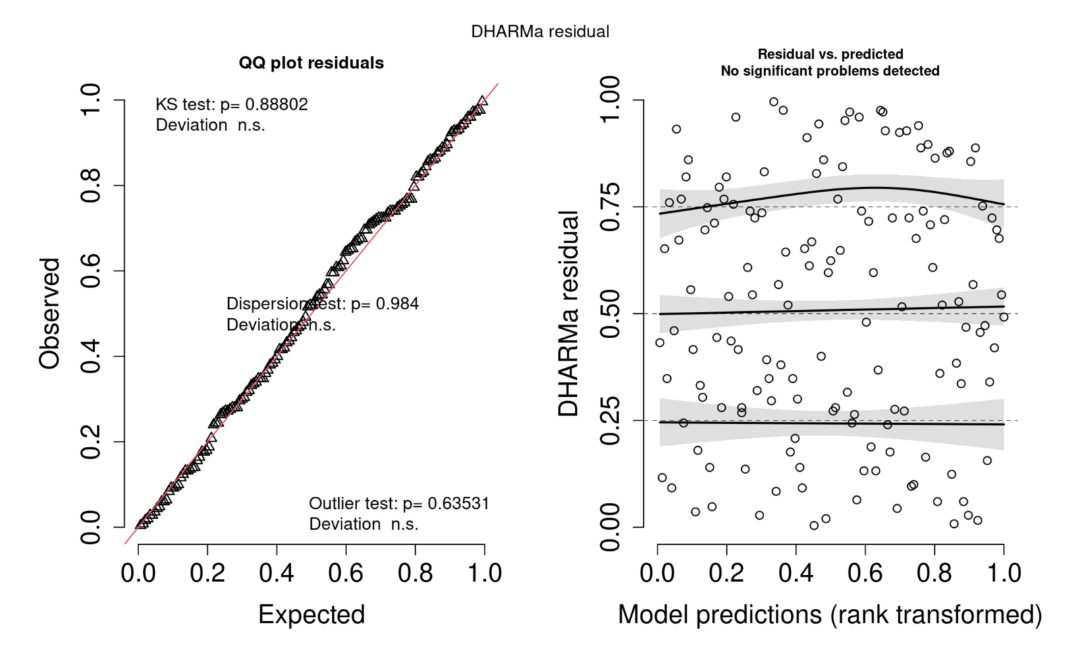


1. Faith’s phylogenetic diversity


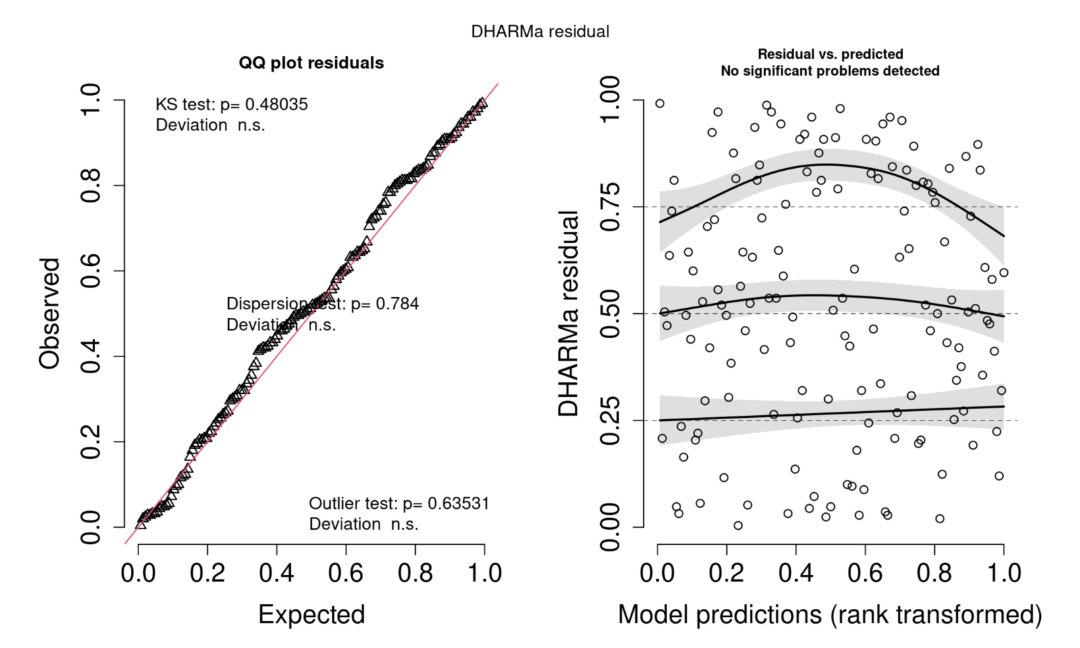


Supplementary Figure 3a, b, c. Model diagnostic plots and tests of simulated residuals (output from the package *DHARMa* v.0.4.6 that examines whether there are substantial deviations from model assumptions by examining uniformity of residuals, over/under dispersion, and occurrence of outliers). Diagnostic analyses completed for generalised linear mixed effects models (GLMMs) that examine predictors of variation in three measures of alpha diversity in gut bacteria from bank voles (*Clethrionomys glareolus*) inhabiting contaminated and uncontaminated areas or field enclosures located within the Chornobyl Exclusion Zone (CEZ), Ukraine. Model predictors were the fixed effects of Treatment * Time + sex + body weight and the random effects of capture location (or field enclosure) and animal identity. Measures of alpha diversity were (a) observed features, (b) Shannon index, and (c) Faith’s phylogenetic diversity.

1. Observed features


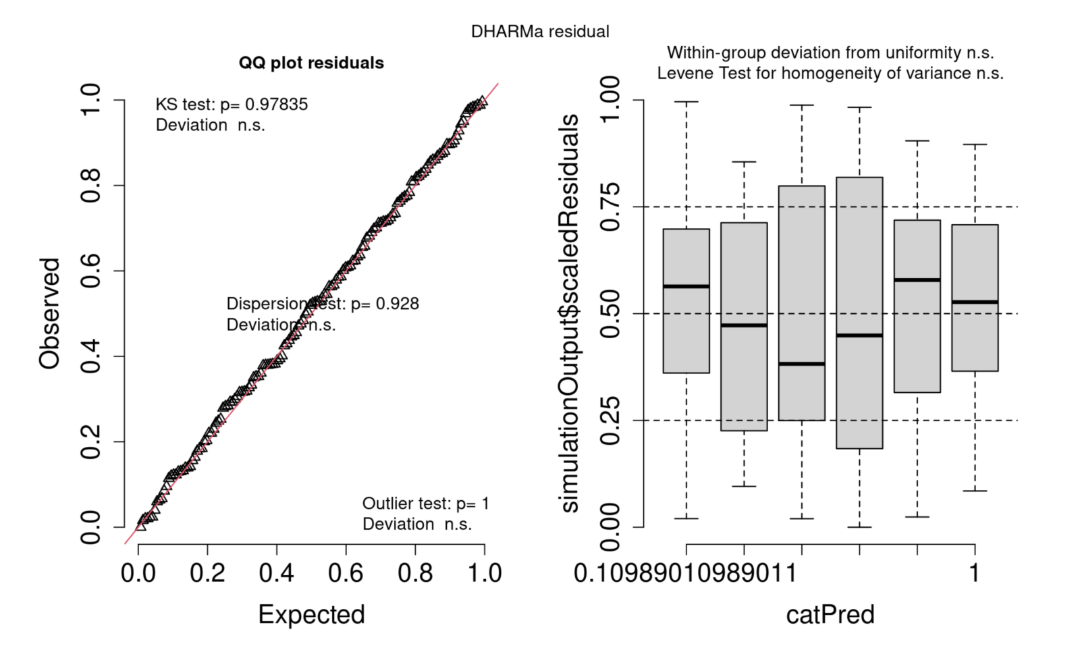


1. Shannon index


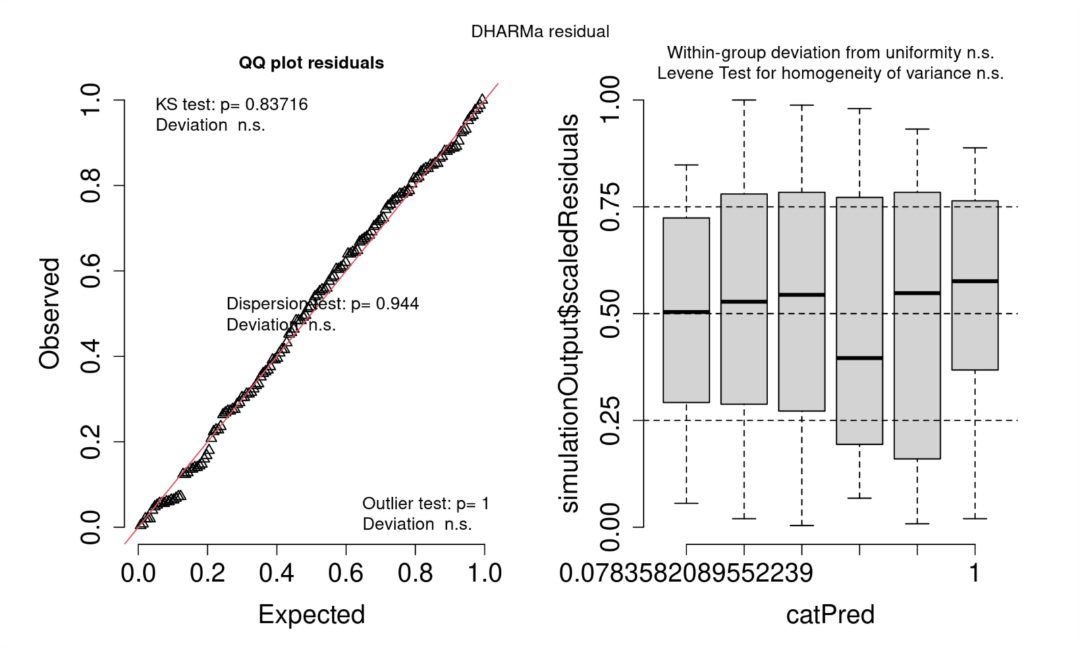


1. Faith’s phylogenetic diversity


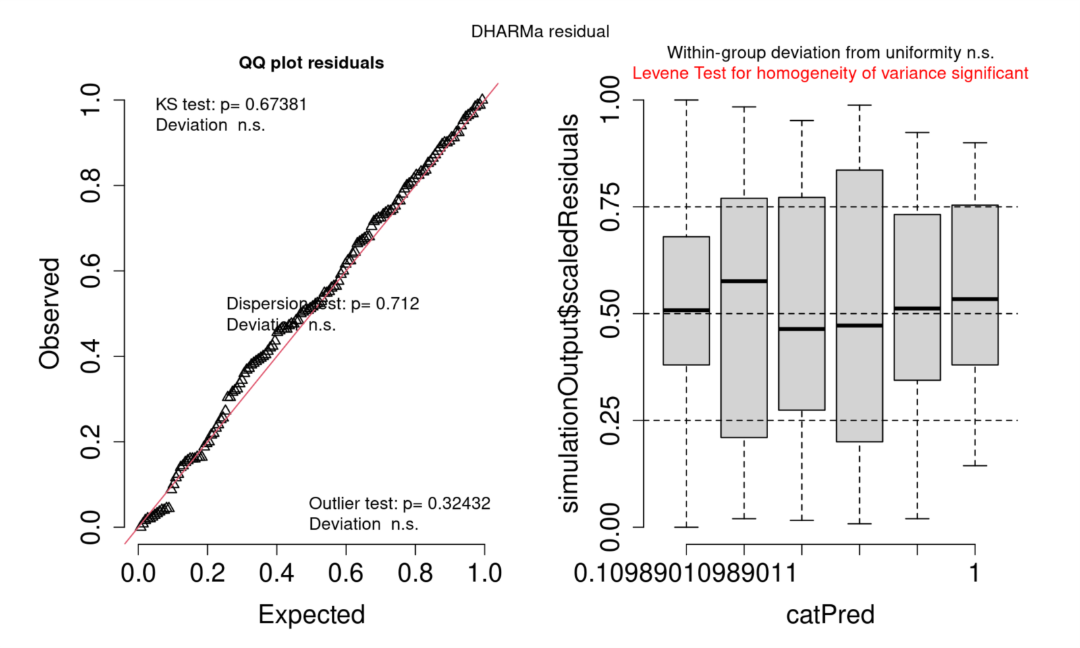


Supplementary Figure 4a, b, c. Model diagnostic plots and tests of simulated residuals (output from the package *DHARMa* v.0.4.6 that examines whether there are substantial deviations from model assumptions by examining uniformity of residuals, over/under dispersion, and occurrence of outliers). Diagnostic analyses completed for generalised linear mixed effects models (GLMMs) that examine predictors of variation in three measures of alpha diversity in gut bacteria from bank voles (*Clethrionomys glareolus*) inhabiting contaminated and uncontaminated areas or field enclosures located within the Chornobyl Exclusion Zone (CEZ), Ukraine. Model predictors were the fixed effects of Treatment + Time, with the random effects of capture location (or enclosure) and animal identity. Measures of alpha diversity were (a) observed features, (b) Shannon index, and (c) Faith’s phylogenetic diversity.

1.
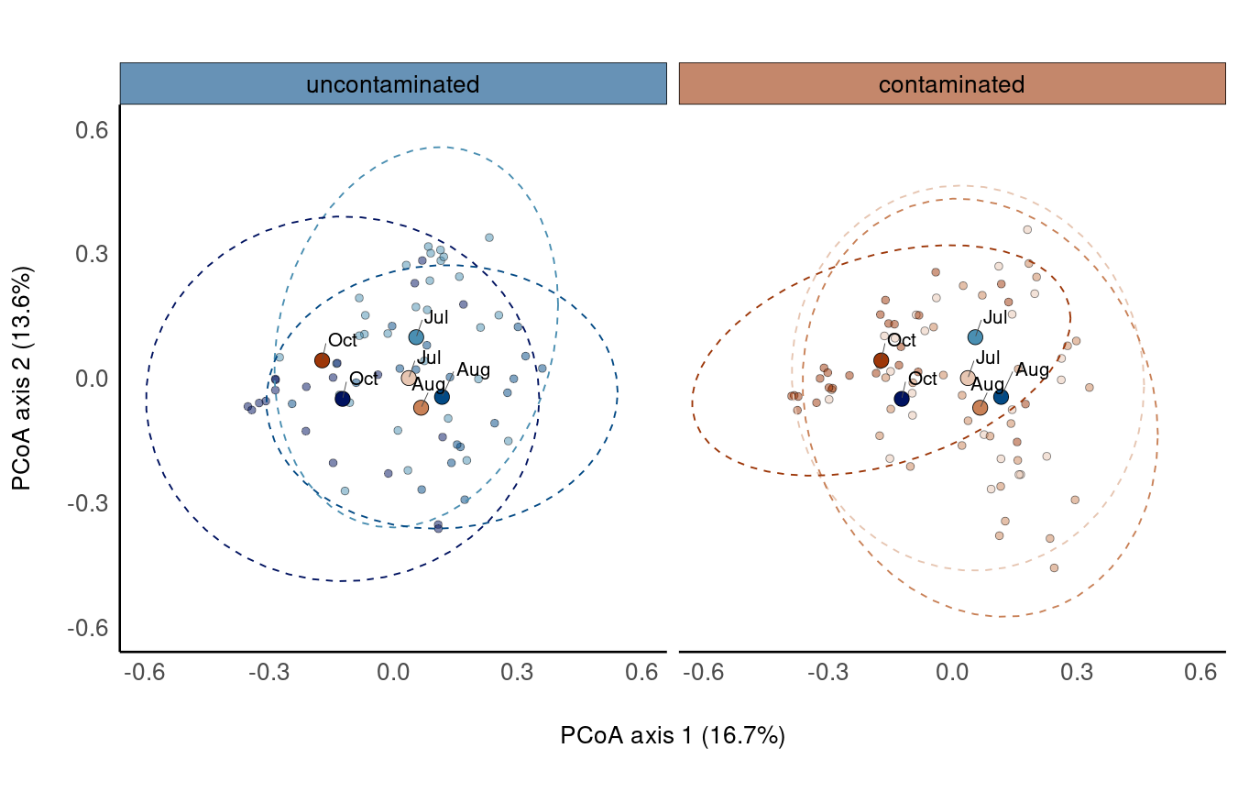
Bray Curtis
2. Unweighted UniFrac


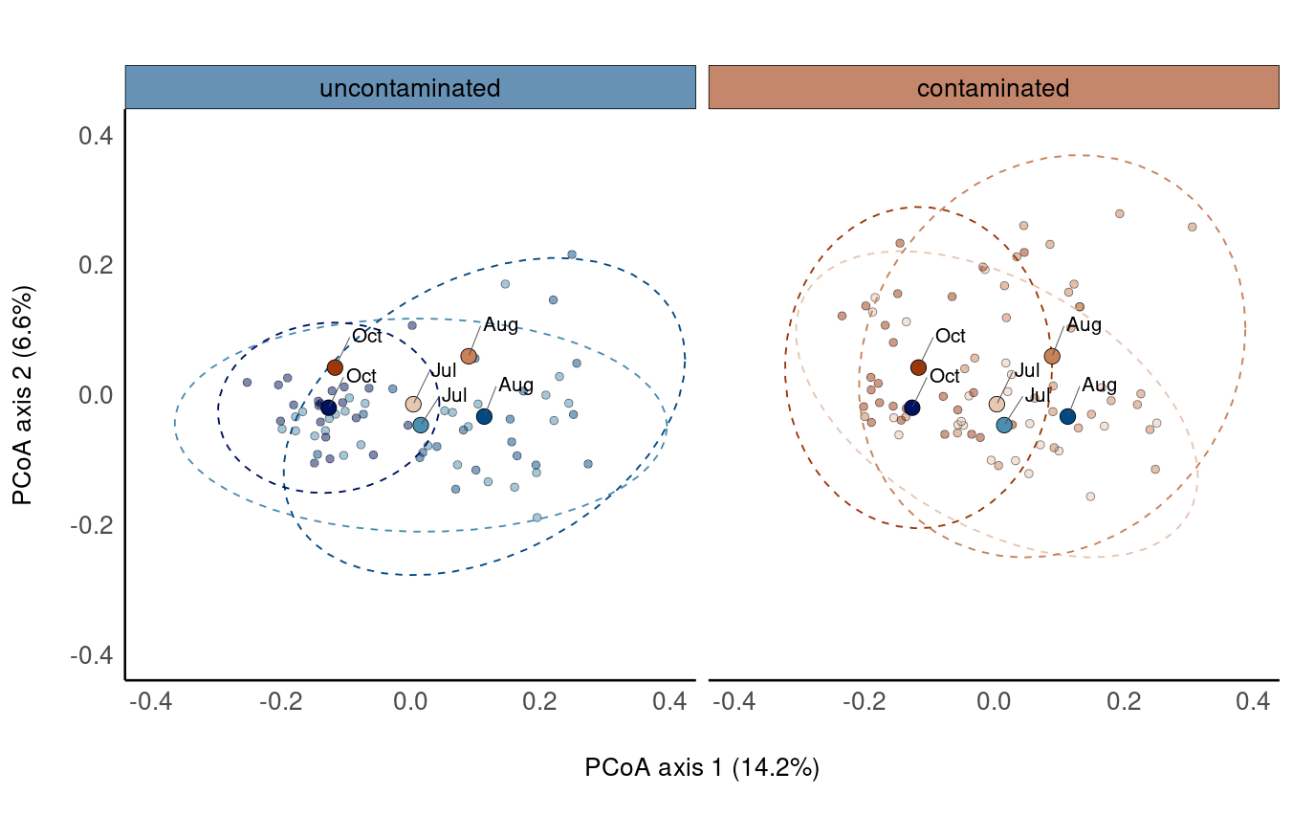


1. Weighted UniFrac


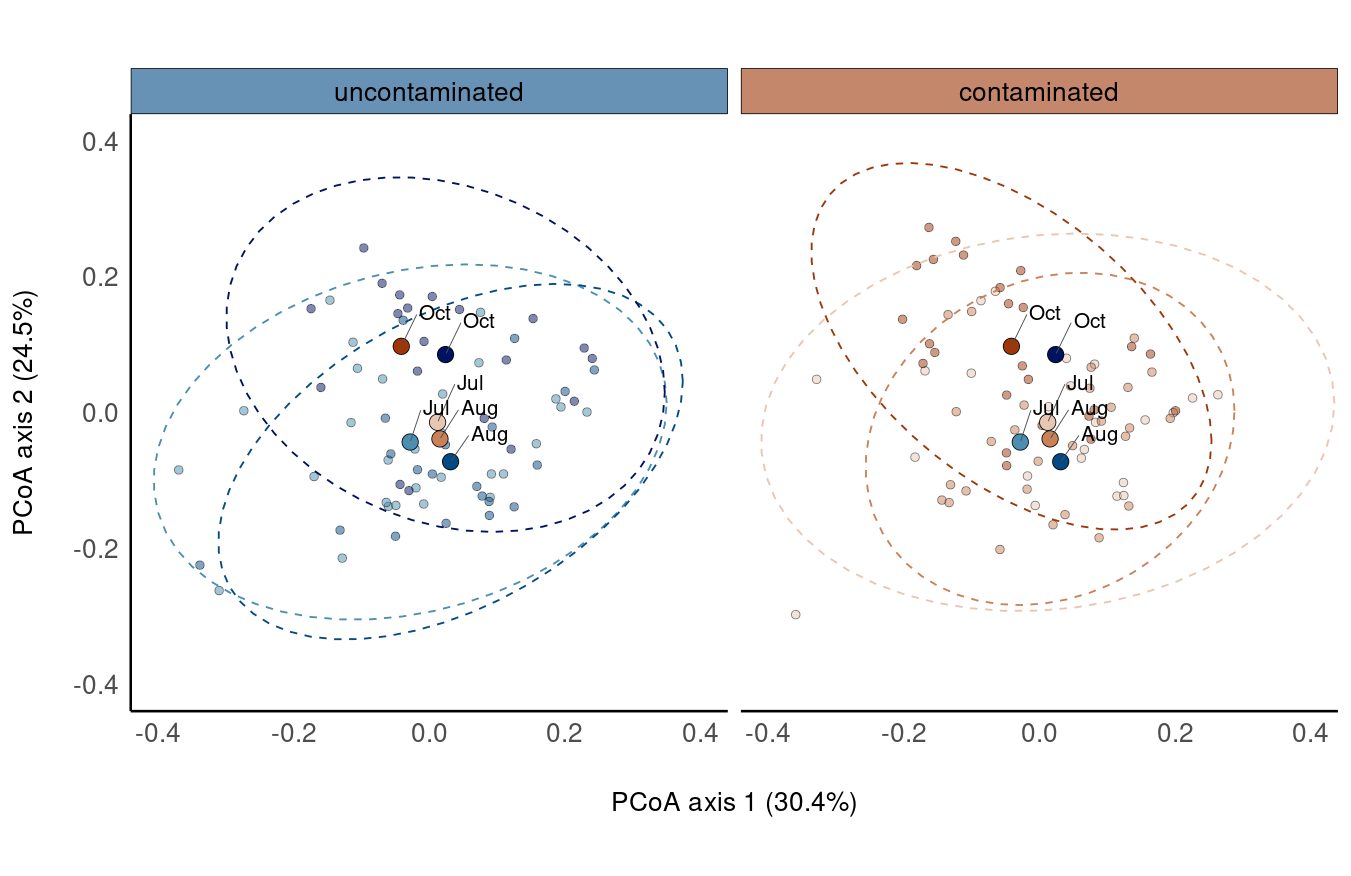


Supplementary Figures 5a, b, c. Temporal (during July, August, and October) variation in three measures of beta diversity ((a) Bray Curtis index, (b) unweighted UniFrac metric, and (c) weighted UniFrac metric) for gut bacteria of bank voles (*Clethrionomys glareolus*) inhabiting contaminated (brown) and uncontaminated (blue) areas (July) or field enclosures (August and October) located within the Chornobyl Exclusion Zone (CEZ), Ukraine. Individual samples and confidence ellipses are shown in separate, faceted panels by the level of radionuclide contamination for clarity, while group centroids are displayed in both panels to facilitate comparison of group-level changes (see Table 1 for summary of PERMANOVA analyses). The principal coordinate analysis (PCoA) visualisation of Jaccard’s dissimilarity for the same samples is provided in Figure 3. Statistical testing for a difference in mean (centroid) beta diversity among groups are given in Table 1 (main text) and in Supplementary Tables 4a, b.


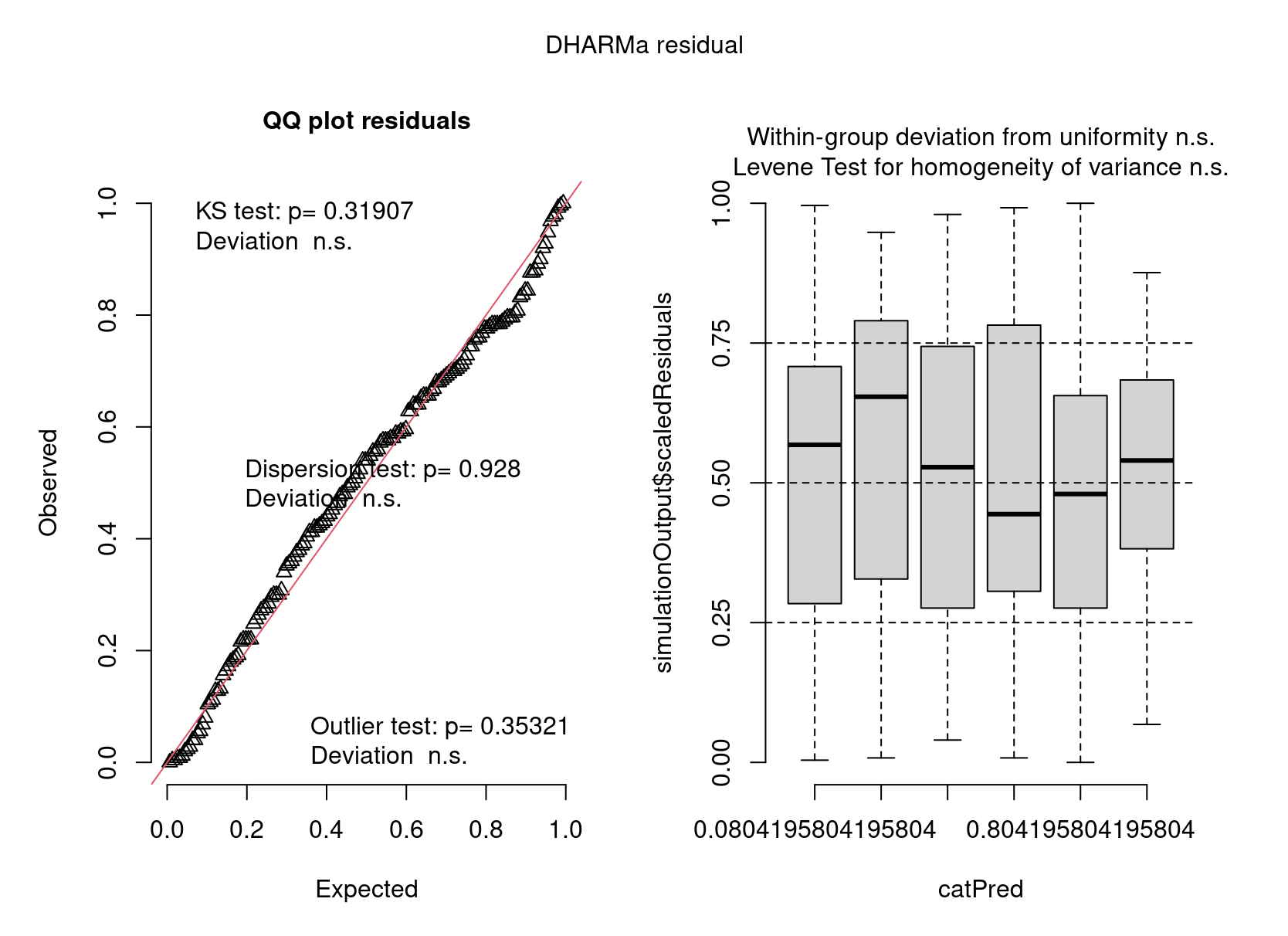


Supplementary Figure 6. Model diagnostic plots and tests of simulated residuals (output from the package *DHARMa* v.0.4.6 that examines whether there are substantial deviations from model assumptions by examining uniformity of residuals, over/under dispersion, and occurrence of outliers). Diagnostic analyses completed for generalised linear mixed effects model (GLMM) that examine predictors of variation in the ratio of the phyla of gut bacteria Firmicutes (=Bacillota) and Bacteroidetes (=Bacteroidota) (F:B ratio calculated as centre log ratio, CLR) from bank voles (*Clethrionomys glareolus*) inhabiting contaminated and uncontaminated areas (July) or field enclosures (August and October) located within the Chornobyl Exclusion Zone (CEZ), Ukraine. Model predictors were the fixed effects of Treatment + Time, with the random effects of capture location (or enclosure) and animal identity. See Table 2 for model results.


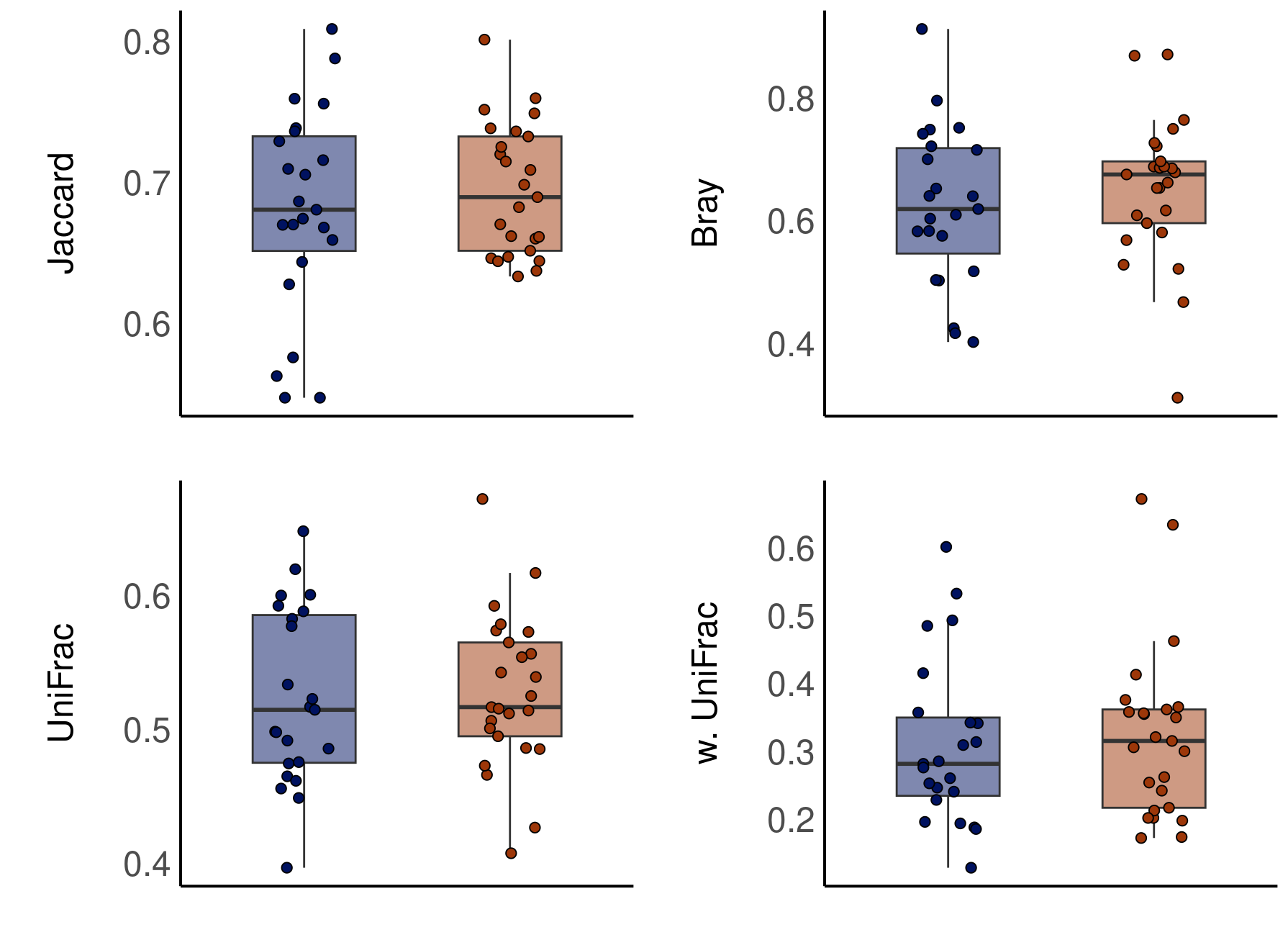


Supplementary Figure 7. Longitudinal changes within individuals (between successive sampling times in July, August, and October) in four metrics of beta diversity for gut bacteria of bank voles (*Clethrionomys glareolus*) inhabiting contaminated (brown) and uncontaminated (blue) areas and field enclosures located within the Chornobyl Exclusion Zone (CEZ), Ukraine.

1. Jaccard


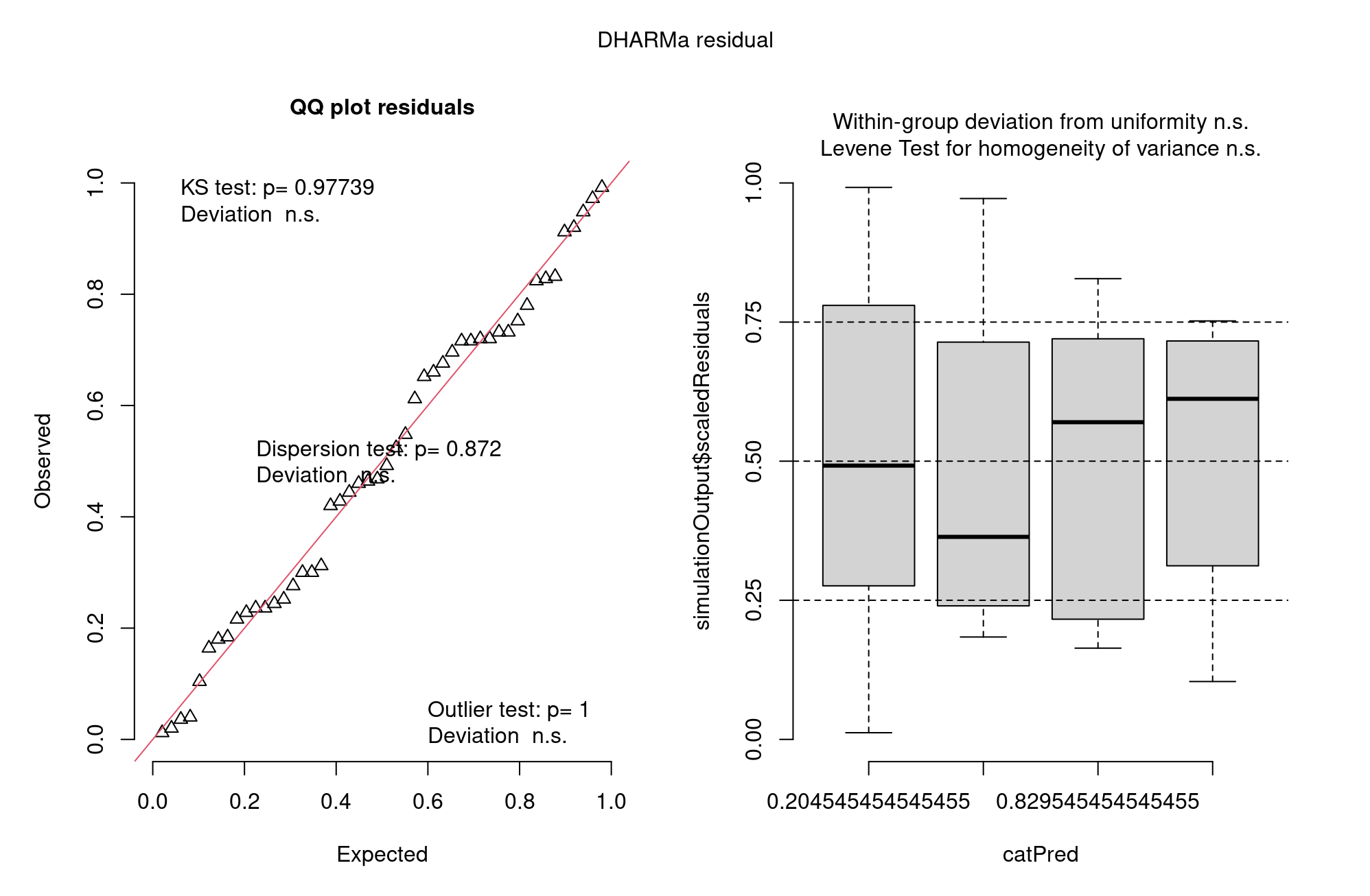


1. Bray


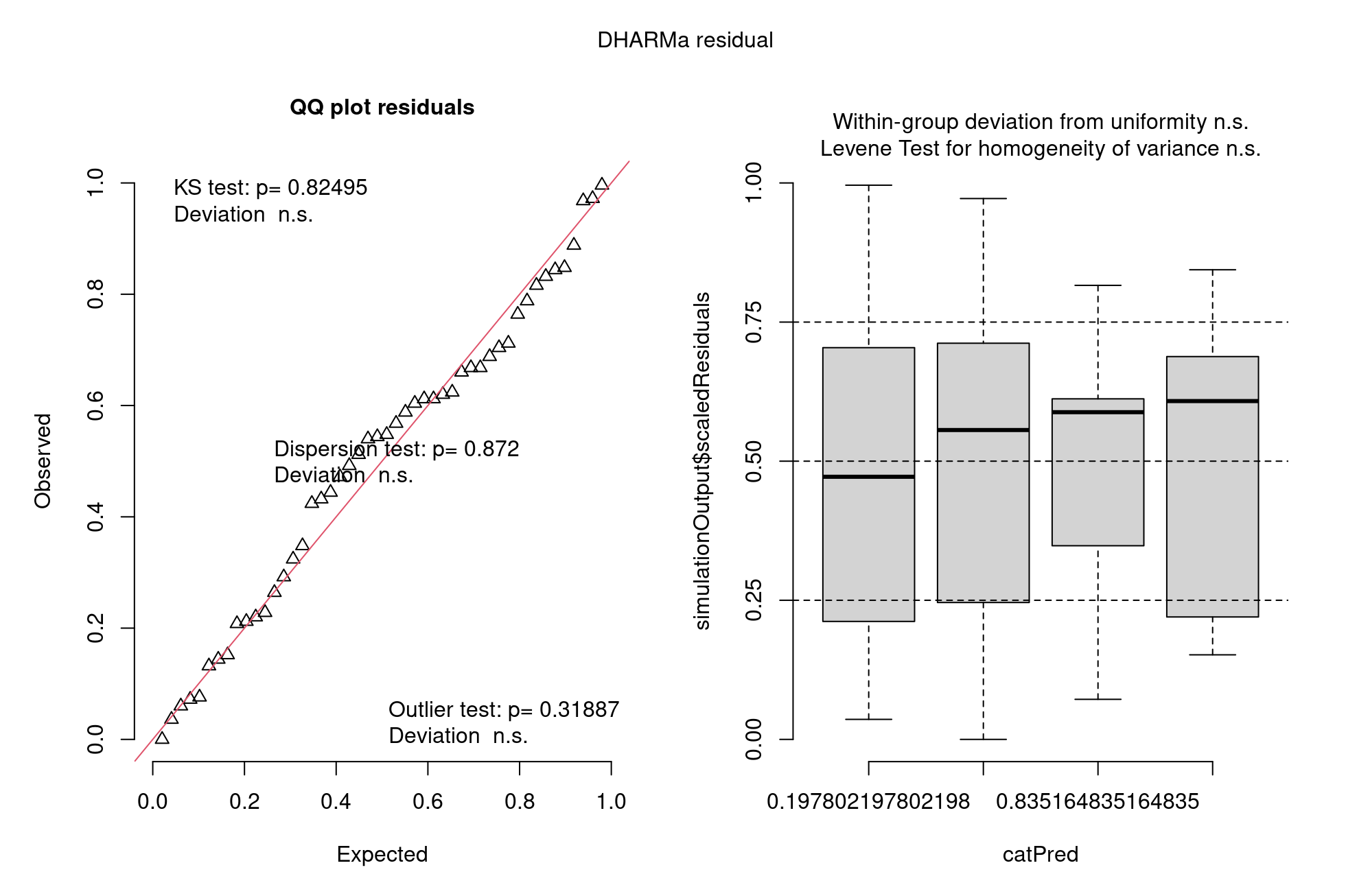


1. UniFrac


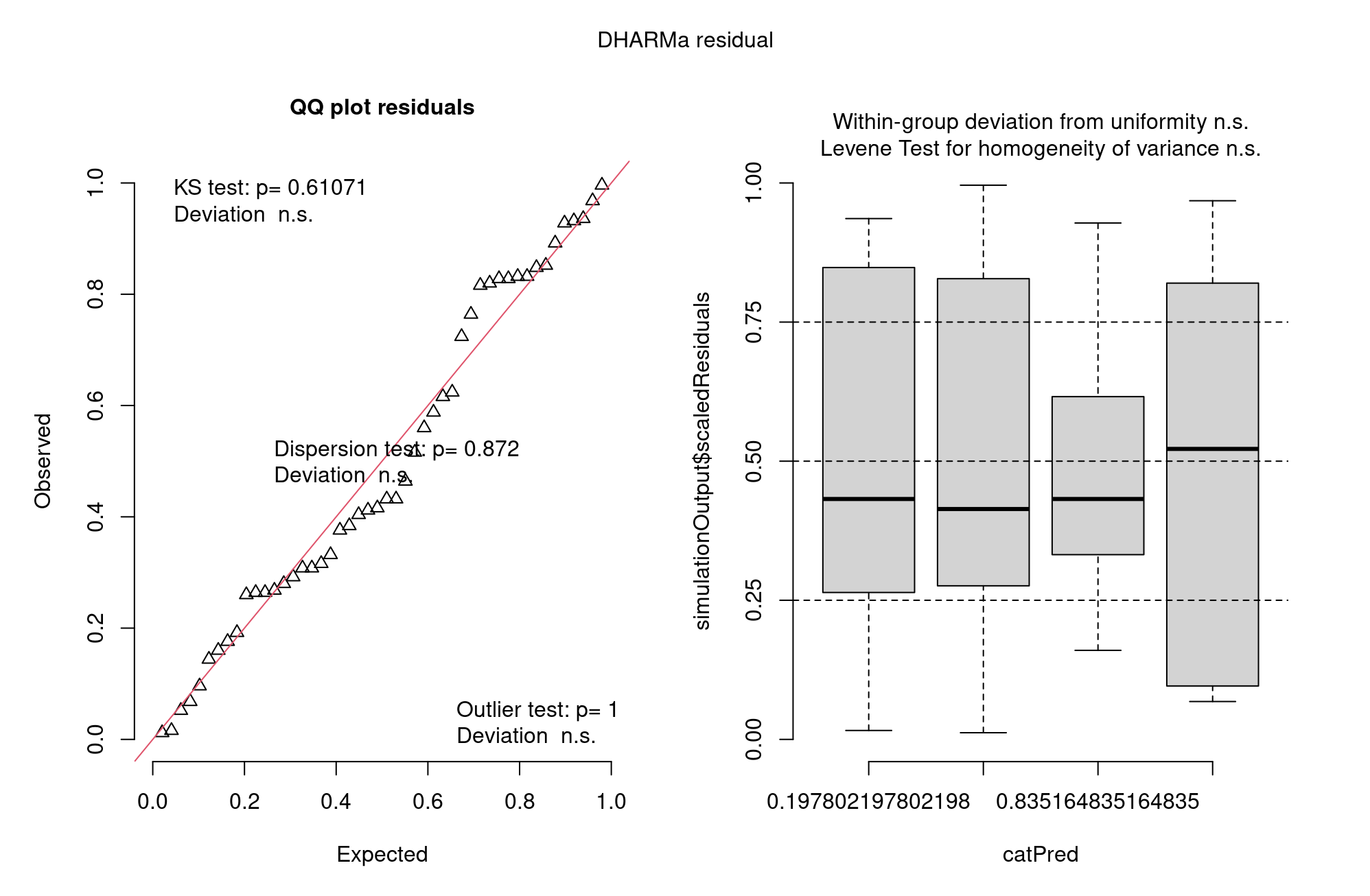


1. weighted UniFrac


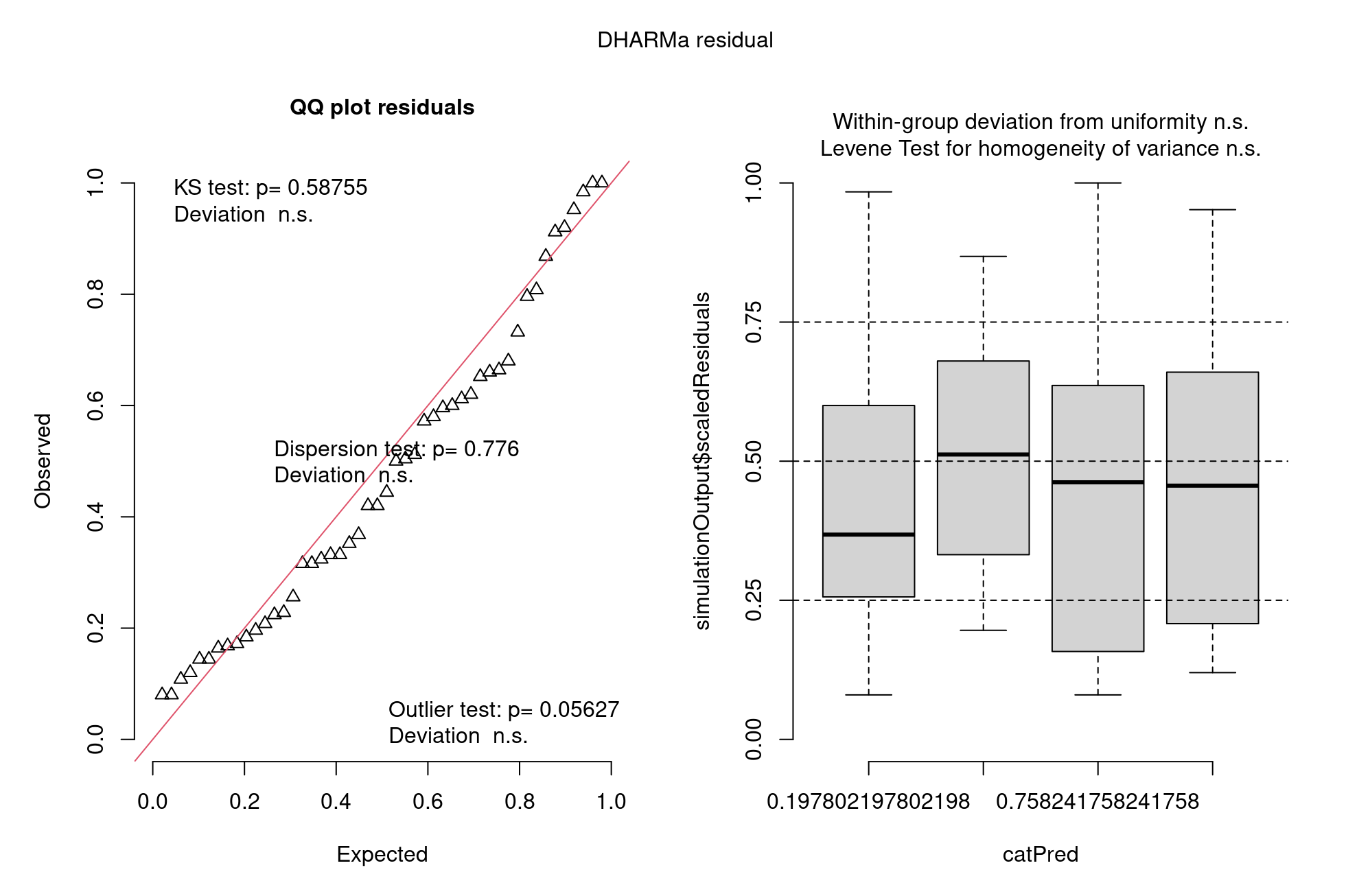


Supplementary Figures 8a, b, c, d. Model diagnostic plots and tests of simulated residuals (output from the package *DHARMa* v.0.4.6 that examines whether there are substantial deviations from model assumptions by examining uniformity of residuals, over/under dispersion, and occurrence of outliers). Diagnostic analyses completed for generalised linear mixed effects models (GLMMs) that examine predictors of variation in mean temporal change in beta diversity (for four measures of beta diversity) in gut bacteria from bank voles (*Clethrionomys glareolus*) inhabiting contaminated and uncontaminated areas or field enclosures located within the Chornobyl Exclusion Zone (CEZ), Ukraine. Model predictors were the fixed effects of Treatment and Time interval and their interaction, with the random effects of capture location (or enclosure) and animal identity. Measures of beta diversity were (a) Jaccard’s index, (b) Bray-Curtis index, (c) UniFrac metric, and (d) weighted UniFrac metric.

1. Jaccard


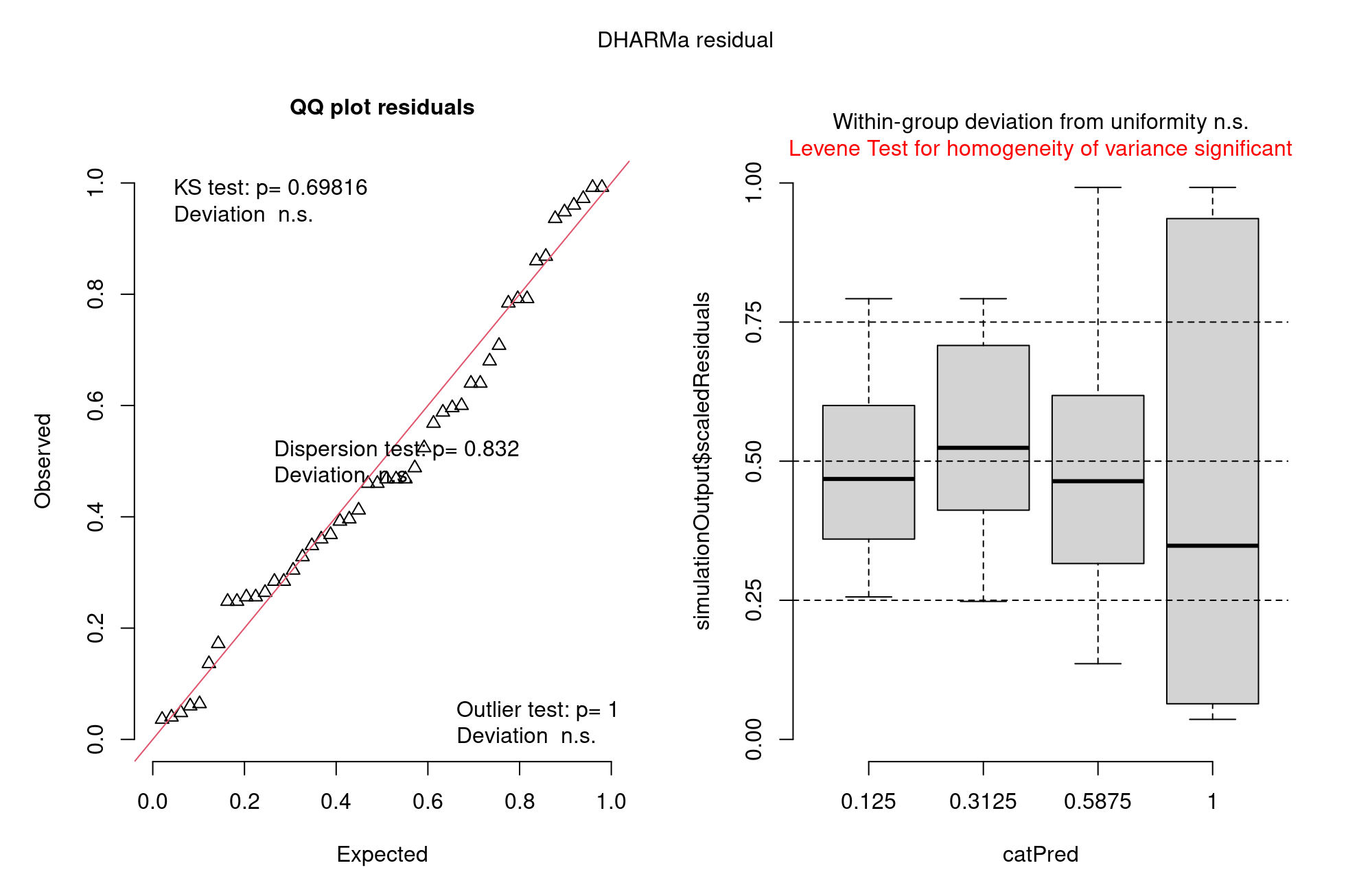


1. Bray


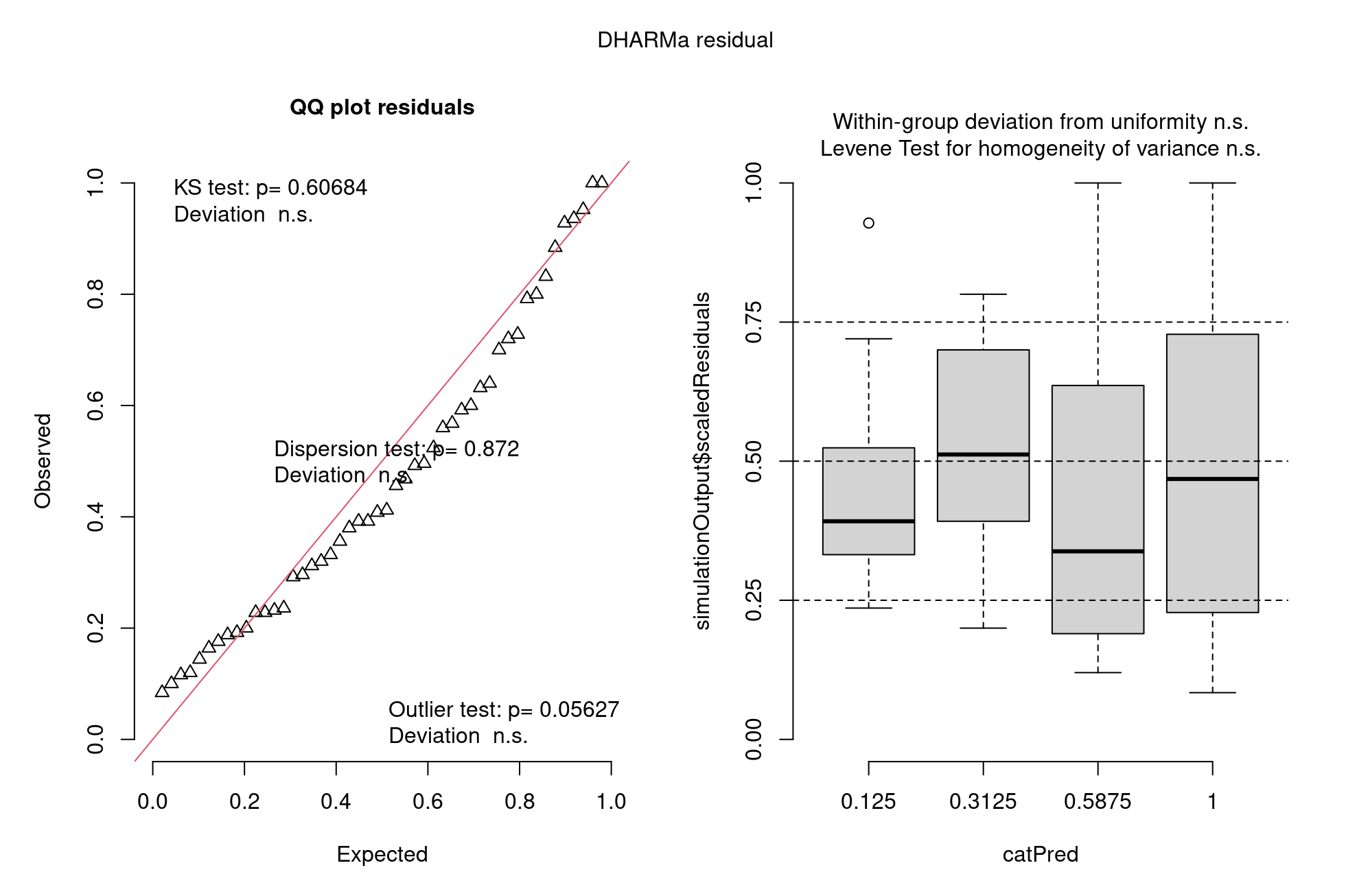


1. UniFrac


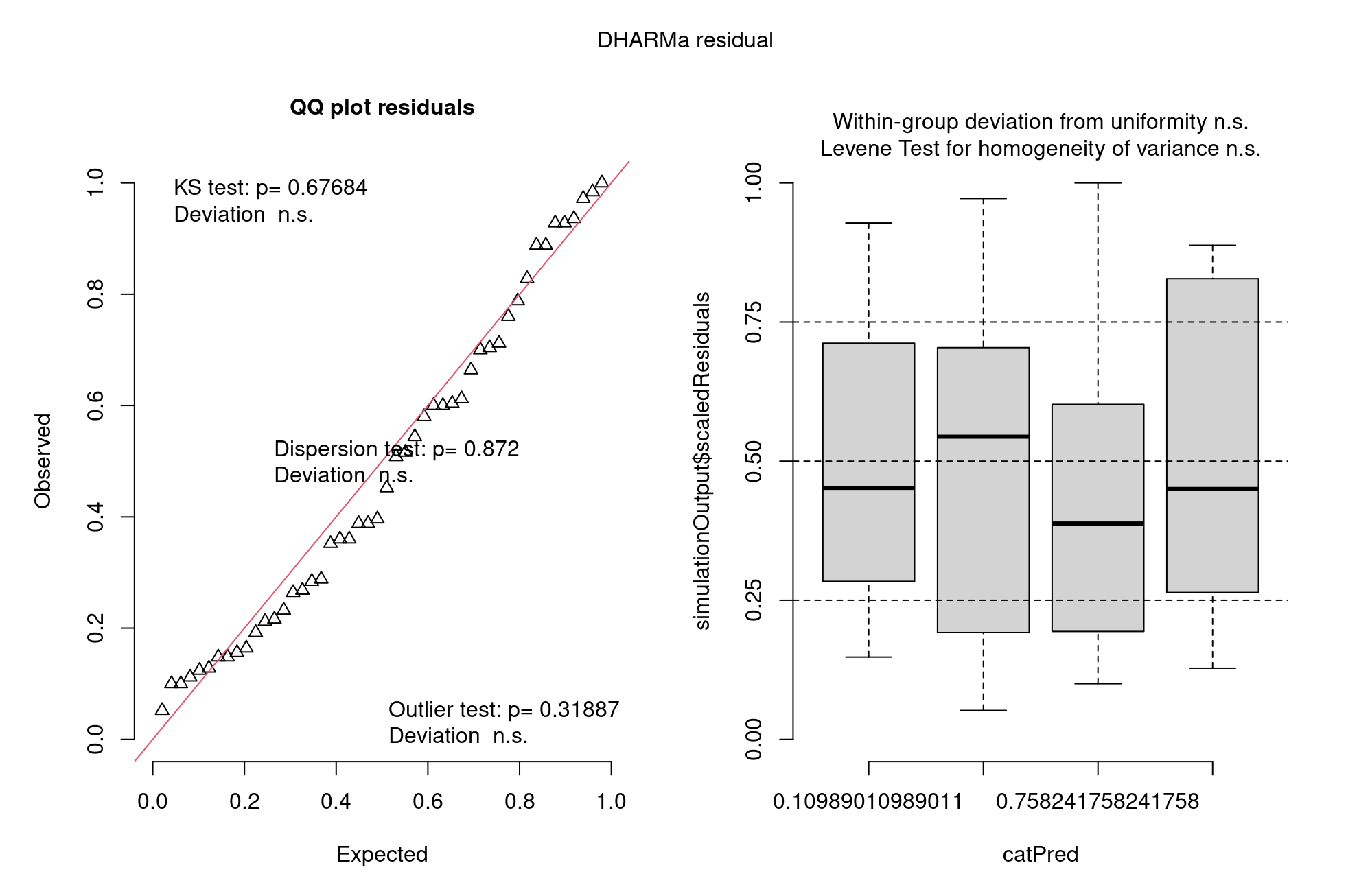


1. weighted UniFrac


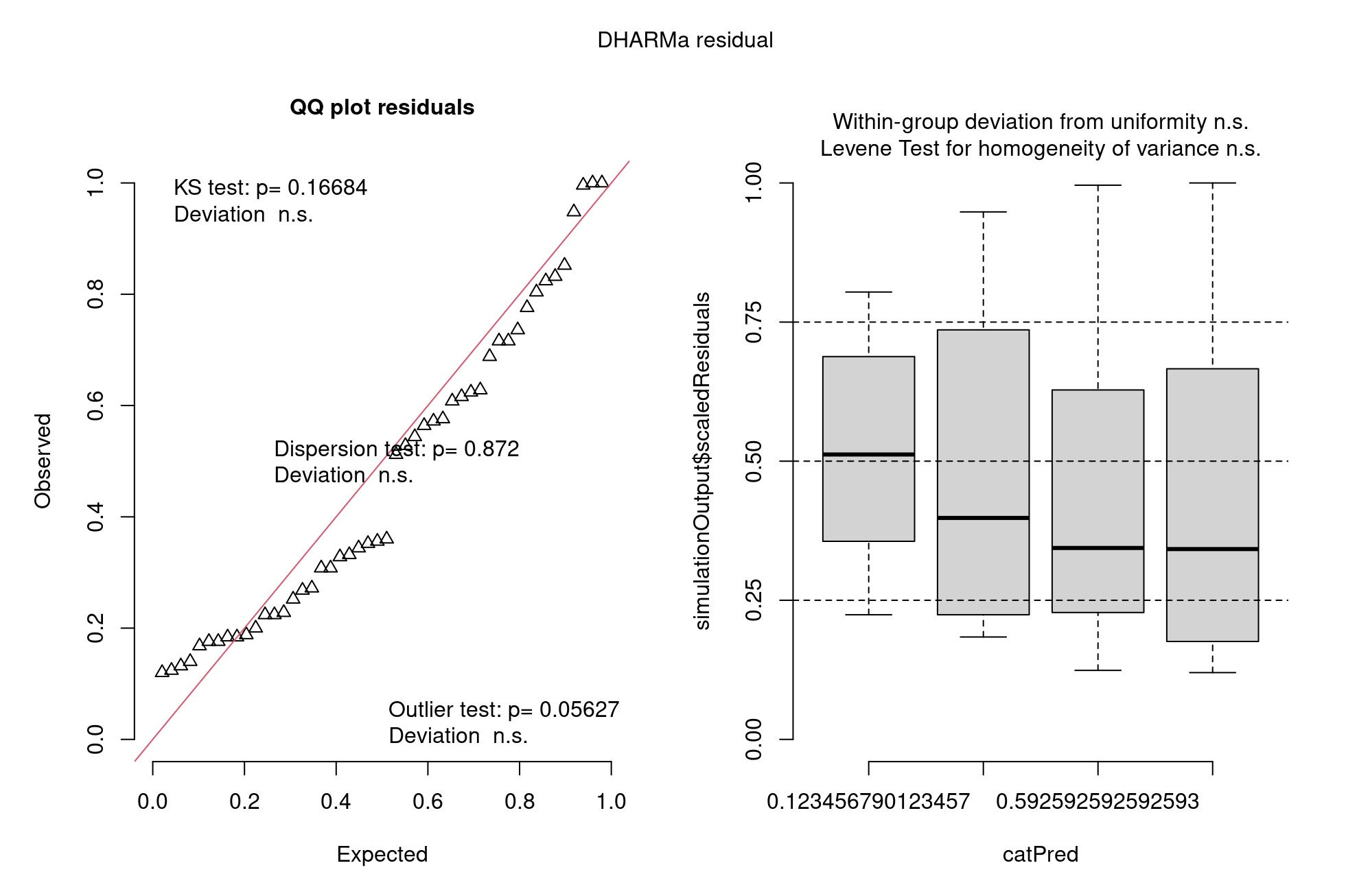


Supplementary Figures 9a, b, c, d. Model diagnostic plots and tests of simulated residuals (output from the package *DHARMa* v.0.4.6 that examines whether there are substantial deviations from model assumptions by examining uniformity of residuals, over/under dispersion, and occurrence of outliers). Diagnostic analyses completed for generalised linear mixed effects models (GLMMs) that examine predictors of variation in the dispersion of temporal changes in beta diversity, quantified as the absolute deviation of pairwise temporal distances from their group mean, in gut bacteria from bank voles (*Clethrionomys glareolus*) inhabiting contaminated and uncontaminated areas or field enclosures located within the Chornobyl Exclusion Zone (CEZ), Ukraine. Model predictors were the fixed effects of Treatment and Time interval and their interaction, with the random effects of capture location (or enclosure) and animal identity. Measures of beta diversity were (a) Jaccard’s index, (b) Bray-Curtis index, (c) UniFrac metric, and (d) weighted UniFrac metric.

Supplementary Table 1. Sample sizes and summary statistics for absorbed doses of radiation experienced by bank voles (*Clethrionomys glareolus*) inhabiting contaminated and uncontaminated areas (July) or field enclosures (August and October) located within the Chornobyl Exclusion Zone (CEZ), Ukraine. *n*, sample size; *IQR*, interquartile range.

|  |  |  | absorbed dose (mGy) | | | |
| --- | --- | --- | --- | --- | --- | --- |
| Treatment | Time | *n* | median | *IQR* | minimum | maximum |
| contaminated | July | 23 | 38.7 | 116 | 9.4 | 292.0 |
|  | August | 29 | 122 | 91.1 | 43.1 | 342.0 |
|  | October | 25 | 225 | 111 | 52.9 | 367.0 |
| uncontaminated | July | 29 | 0.53 | 0.42 | 0.216 | 1.32 |
|  | August | 20 | 0.64 | 0.30 | 0.365 | 1.59 |
|  | October | 20 | 1.66 | 5.61 | 0.400 | 23.3 |

Supplementary Table 2. Statistical significance of pairwise tests (Dunn’s pairwise comparisons with Benjamini-Hochberg correction for multiple testing) for differences in the absorbed dose of radiation experienced by bank voles (*Clethrionomys glareolus*) inhabiting contaminated and uncontaminated areas (July) or field enclosures (August and October) located within the Chornobyl Exclusion Zone (CEZ), Ukraine. *Z*, test statistic; *p*, statistical significance; *p.adj*, adjusted statistical significance).

| comparison |  | *Z* | *p* | *p.adj.* |
| --- | --- | --- | --- | --- |
| uncontaminated Jul | uncontaminated Aug | -0,751 | 0,453 | 0,453 |
| uncontaminated Jul | uncontaminated Oct | -2,272 | 0,023 | 0,031 |
| uncontaminated Aug | uncontaminated Oct | -1,370 | 0,171 | 0,197 |
| contaminated Jul | contaminated Aug | -1,065 | 0,287 | 0,308 |
| contaminated Jul | contaminated Oct | -2,605 | 0,009 | 0,014 |
| contaminated Aug | contaminated Oct | -1,623 | 0,105 | 0,131 |
|  |  |  |  |  |
| contaminated Jul | uncontaminated Jul | 5,753 | 0,000 | 0,000 |
| contaminated Aug | uncontaminated Aug | 5,633 | 0,000 | 0,000 |
| contaminated Oct | uncontaminated Oct | 5,662 | 0,000 | 0,000 |
|  |  |  |  |  |
| contaminated Jul | uncontaminated Aug | 4,467 | 0,000 | 0,000 |
| contaminated Jul | uncontaminated Oct | 3,094 | 0,002 | 0,003 |
| contaminated Aug | uncontaminated Jul | 7,136 | 0,000 | 0,000 |
| contaminated Aug | uncontaminated Oct | 4,230 | 0,000 | 0,000 |
| contaminated Oct | uncontaminated Jul | 8,644 | 0,000 | 0,000 |
| contaminated Oct | uncontaminated Aug | 7,023 | 0,000 | 0,000 |

Supplementary Table 3a. Likelihood ratio test results for fixed effects in generalized linear mixed-effects models (GLMMs) examining predictors of variation in three measures of alpha diversity in the gut microbiota of bank voles (*Clethrionomys glareolus*) from contaminated and uncontaminated areas in the Chornobyl Exclusion Zone (CEZ), Ukraine. (a) observed features, (b) Shannon index, and (c) Faith’s phylogenetic diversity. Chisq., chi-square statistic; *df*, degrees of freedom; *p*, statistical significance.

| **Predictor** | **Chisq.** | ***df*** | ***p*** |
| --- | --- | --- | --- |
| **(a) observed features** |  |  |  |
| (Intercept) | 3519.432 | 1 | <0.001 |
| Treatment | 0.512 | 1 | 0.474 |
| Time | 25.902 | 2 | <0.001 |
| Sex | 1.334 | 1 | 0.248 |
| Body Mass | 0.451 | 1 | 0.502 |
| Treatment:Time | 0.962 | 2 | 0.618 |
|  |  |  |  |
| **(b) Shannon index** |  |  |  |
| (Intercept) | 548.430 | 1 | <0.001 |
| Treatment | 0.060 | 1 | 0.807 |
| Time | 27.451 | 2 | <0.001 |
| Sex | 0.468 | 1 | 0.494 |
| Body Mass | 2.031 | 1 | 0.154 |
| Treatment:Time | 0.678 | 2 | 0.712 |
|  |  |  |  |
| **(c) Faith's phylogenetic diversity** |  |  |  |
| (Intercept) | 1683.155 | 1 | <0.001 |
| Treatment | 0.254 | 1 | 0.615 |
| Time | 24.960 | 2 | <0.001 |
| Sex | 1.875 | 1 | 0.171 |
| Body Mass | 0.434 | 1 | 0.510 |
| Treatment:Time | 0.862 | 2 | 0.650 |

Supplementary Table 3b. Statistical significance of generalised linear mixed effects models (GLMMs) (with an interaction between Treatment and Time) that examine predictors of variation in three measures of alpha diversity in gut bacteria of bank voles (*Clethrionomys glareolus*) inhabiting contaminated and uncontaminated areas or field enclosures located within the Chornobyl Exclusion Zone (CEZ), Ukraine. (a) observed features, (b) Shannon index, and (c) Faith’s phylogenetic diversity. Est., estimate; SE, standard Error; CI lower/CI upper, lower and upper confidence intervals; *p*, statistical significance.

| **Predictor** | **Est.** | **SE** | **CI lower** | **CI upper** | ***p*** |
| --- | --- | --- | --- | --- | --- |
| **(a) observed features** |  |  |  |  |  |
| (Intercept) | 4.865 | 0.082 | 4.704 | 5.026 | <0.001 |
| Treatment - uncontaminated | 0.076 | 0.106 | -0.132 | 0.285 | 0.474 |
| Time - Aug | -0.149 | 0.111 | -0.368 | 0.069 | 0.180 |
| Time - Oct | 0.228 | 0.114 | 0.004 | 0.453 | 0.046 |
| Sex - Male | 0.057 | 0.049 | -0.039 | 0.153 | 0.248 |
| Body Mass | -0.018 | 0.027 | -0.071 | 0.035 | 0.502 |
| Treatment - Uncontaminated:Time - Aug | -0.144 | 0.160 | -0.457 | 0.170 | 0.369 |
| Treatment - Uncontaminated: Time - Oct | -0.070 | 0.161 | -0.385 | 0.246 | 0.666 |
| marginal R² | 0.302 |  |  |  |  |
| conditional R² | 0.379 |  |  |  |  |
| **(b) Shannon index** | **Est.** | **SE** | **CI lower** | **CI upper** | **p** |
| (Intercept) | 3.386 | 0.145 | 3.103 | 3.669 | <0.001 |
| Treatment - uncontaminated | 0.046 | 0.188 | -0.322 | 0.414 | 0.807 |
| Time - Aug | -0.229 | 0.179 | -0.580 | 0.122 | 0.201 |
| Time - Oct | 0.572 | 0.190 | 0.200 | 0.944 | 0.003 |
| Sex - Male | 0.072 | 0.105 | -0.134 | 0.278 | 0.494 |
| Body Mass | -0.084 | 0.059 | -0.201 | 0.032 | 0.154 |
| Treatment - Uncontaminated:Time - Aug | -0.193 | 0.269 | -0.721 | 0.335 | 0.473 |
| Treatment - Uncontaminated:Time - Oct | -0.222 | 0.284 | -0.780 | 0.335 | 0.435 |
| marginal R² | 0.309 |  |  |  |  |
| conditional R² | 0.430 |  |  |  |  |
| **(c) Faith's phylogenetic diversity** | **Est.** | **SE** | **CI lower** | **CI upper** | **p** |
| (Intercept) | 2.387 | 0.058 | 2.273 | 2.501 | <0.001 |
| Treatment - uncontaminated | 0.038 | 0.075 | -0.110 | 0.186 | 0.615 |
| Time - Aug | -0.154 | 0.080 | -0.310 | 0.002 | 0.053 |
| Time - Oct | 0.117 | 0.082 | -0.044 | 0.278 | 0.154 |
| Sex - Male | 0.049 | 0.036 | -0.021 | 0.119 | 0.171 |
| Body Mass | -0.013 | 0.020 | -0.052 | 0.026 | 0.510 |
| Treatment - Uncontaminated:Time - Aug | -0.041 | 0.112 | -0.261 | 0.179 | 0.715 |
| Treatment - Uncontaminated:Time - Oct | 0.034 | 0.114 | -0.189 | 0.257 | 0.766 |
| marginal R² | 0.307 |  |  |  |  |
| conditional R² | 0.378 |  |  |  |  |

Supplementary Table 3c. Statistical significance of generalised linear mixed effects models (GLMMs) that examine predictors (only the main effects of Treatment and Time) of variation in three measures of alpha diversity in gut bacteria of bank voles (*Clethrionomys glareolus*) inhabiting contaminated and uncontaminated areas or field enclosures located within the Chornobyl Exclusion Zone (CEZ), Ukraine. (a) observed features, (b) Shannon index, and (c) Faith’s phylogenetic diversity. Est., estimate; SE, standard Error; CI lower/CI upper, lower and upper confidence intervals; *p*, statistical significance.

| **Predictor** | **Est.** | **SE** | **CI lower** | **CI upper** | ***p*** |
| --- | --- | --- | --- | --- | --- |
| **(a) observed features** |  |  |  |  |  |
| (Intercept) | 4.925 | 0.060 | 4.808 | 5.043 | <0.001 |
| Treatment - uncontaminated | 0.017 | 0.067 | -0.115 | 0.148 | 0.803 |
| Time - Aug | -0.224 | 0.071 | -0.363 | -0.085 | 0.002 |
| Time - Oct | 0.194 | 0.071 | 0.054 | 0.334 | 0.007 |
| marginal R² | 0.285 |  |  |  |  |
| conditional R² | 0.339 |  |  |  |  |
|  |  |  |  |  |  |
| **(b) Shannon index** |  |  |  |  |  |
| (Intercept) | 3.478 | 0.106 | 3.271 | 3.685 | <0.001 |
| Treatment - uncontaminated | -0.090 | 0.116 | -0.317 | 0.137 | 0.436 |
| Time - Aug | -0.346 | 0.115 | -0.571 | -0.120 | 0.003 |
| Time - Oct | 0.536 | 0.121 | 0.298 | 0.774 | <0.001 |
| marginal R² | 0.292 |  |  |  |  |
| conditional R² | 0.427 |  |  |  |  |
|  |  |  |  |  |  |
| **(c) Faith's phylogenetic diversity** |  |  |  |  |  |
| (Intercept) | 2.412 | 0.044 | 2.325 | 2.498 | <0.001 |
| Treatment - uncontaminated | 0.033 | 0.050 | -0.064 | 0.131 | 0.504 |
| Time - Aug | -0.174 | 0.053 | -0.277 | -0.071 | <0.001 |
| Time - Oct | 0.134 | 0.053 | 0.030 | 0.238 | 0.012 |
| marginal R² | 0.284 |  |  |  |  |
| conditional R² | 0.341 |  |  |  |  |

Supplementary Table 4a. Statistical significance (by PERMANOVA) and effect sizes of exposure to environmental radionuclides (Treatment=contaminated or uncontaminated areas) or temporal changes (Time=sampling in July, August, or October) on variation in beta diversity of gut bacteria from the bank vole (*Clethrionomys glareolus*) inhabiting contaminated and uncontaminated areas (July) or field enclosures (August and October) located within the Chornobyl Exclusion Zone (CEZ), Ukraine. Models were run with (Treatment*Time) and without (Treatment+Time) an interaction between Treatment and Time. *df*, degrees of freedom; *SS*, sum of squares; *MS* mean square; *R^2^*, effect size; *F*, test statistic; *p*, statistical significance. PCoA visualisations of beta diversity are given in Figure 3 main text (Jaccard’s metric) and in Supplementary Figures 5a, b, c (all other metrics).

|  | **Treatment*Time** | | | | |  | **Treatment + Time** | | | | |
| --- | --- | --- | --- | --- | --- | --- | --- | --- | --- | --- | --- |
| **Predictor** | **df** | **SS** | ***R^2^*** | ***F*** | ***p*** |  | **df** | **SS** | ***R^2^*** | ***F*** | ***p*** |
| **(a) Jacccard** |  |  |  |  |  |  | |  |  |  |  |
| Treatment |  |  |  |  |  |  | 1 | 0.462 | 0.013 | 2.022 | <0.001 |
| Time |  |  |  |  |  |  | 2 | 1.855 | 0.052 | 4.060 | <0.001 |
| Sex | 1 | 0.256 | 0.007 | 1.128 | 0.002 |  | 1 | 0.256 | 0.007 | 1.123 | <0.001 |
| Body Mass | 1 | 0.314 | 0.009 | 1.382 | 0.460 |  | 1 | 0.307 | 0.009 | 1.342 | 0.436 |
| Treatment :Time | 2 | 0.644 | 0.018 | 1.417 | 0.048 |  | |  |  |  |  |
| Residual | 138 | 31.337 | 0.884 |  |  |  | 140 | 31.981 | 0.902 |  |  |
| Total | 145 | 35.458 | 1.000 |  |  |  | 145 | 35.458 | 1.000 |  |  |
| **(b) Bray-Curtis** |  |  |  |  |  |  | |  |  |  |  |
| Treatment |  |  |  |  |  |  | 1 | 0.289 | 0.009 | 1.458 | 0.003 |
| Time |  |  |  |  |  |  | 2 | 1.902 | 0.061 | 4.804 | <0.001 |
| Sex | 1 | 0.132 | 0.004 | 0.674 | 0.041 |  | 1 | 0.140 | 0.004 | 0.706 | 0.017 |
| Body Mass | 1 | 0.380 | 0.012 | 1.935 | 0.328 |  | 1 | 0.365 | 0.012 | 1.844 | 0.236 |
| Treatment :Time | 2 | 0.584 | 0.019 | 1.486 | 0.155 |  | |  |  |  |  |
| Residual | 138 | 27.127 | 0.866 |  |  |  | 140 | 27.711 | 0.885 |  |  |
| Total | 145 | 31.321 | 1.000 |  |  |  | 145 | 31.321 | 1.000 |  |  |
| **(c) UniFrac** |  |  |  |  |  |  | |  |  |  |  |
| Treatment |  |  |  |  |  |  | 1 | 0.282 | 0.014 | 2.162 | 0.002 |
| Time |  |  |  |  |  |  | 2 | 1.240 | 0.060 | 4.750 | <0.001 |
| Sex | 1 | 0.124 | 0.006 | 0.954 | 0.004 |  | 1 | 0.121 | 0.006 | 0.930 | 0.002 |
| Body Mass | 1 | 0.178 | 0.009 | 1.368 | 0.312 |  | 1 | 0.171 | 0.008 | 1.307 | 0.375 |
| Treatment :Time | 2 | 0.348 | 0.017 | 1.338 | 0.099 |  | |  |  |  |  |
| Residual | 138 | 17.929 | 0.872 |  |  |  | 140 | 18.277 | 0.889 |  |  |
| Total | 145 | 20.561 | 1.000 |  |  |  | 145 | 20.561 | 1.000 |  |  |
| **(d) weighted UniFrac** |  |  |  |  |  |  | |  |  |  |  |
| Treatment |  |  |  |  |  |  | 1 | 0.058 | 0.007 | 1.133 | 0.003 |
| Time |  |  |  |  |  |  | 2 | 0.598 | 0.073 | 5.876 | <0.001 |
| Sex | 1 | 0.037 | 0.004 | 0.720 | 0.025 |  | 1 | 0.031 | 0.004 | 0.617 | 0.045 |
| Body Mass | 1 | 0.088 | 0.011 | 1.740 | 0.364 |  | 1 | 0.084 | 0.010 | 1.656 | 0.321 |
| Treatment :Time | 2 | 0.136 | 0.017 | 1.339 | 0.075 |  | |  |  |  |  |
| Residual | 138 | 6.992 | 0.853 |  |  |  | 140 | 7.128 | 0.870 |  |  |
| Total | 145 | 8.193 | 1.000 |  |  |  | 145 | 8.193 | 1.000 |  |  |

Supplementary Table 4b. Statistical significance (by PERMANOVA) and effect sizes of exposure to environmental radionuclides (treatment=total absorbed dose in mGy) or temporal changes (time=three sampling times - July, August, or October) on variation in beta diversity of gut bacteria from the bank vole (*Clethrionomys glareolus*) inhabiting contaminated and uncontaminated areas (July) or field enclosures (August and October) located within the Chornobyl Exclusion Zone (CEZ), Ukraine. *df*, degrees of freedom; *SS*, sum of squares; *MS* mean square; *R^2^* , effect size; *F*, test statistic; *p*, statistical significance. PCoA visualisations of beta diversity are given in Figure 3 main text (Jaccard’s metric) and in Supplementary Figures 5a, b, c (all other metrics).

|  | **Total dose (mGy)*Time** | | | | |  | **Total dose (mGy) + Time** | | | | |
| --- | --- | --- | --- | --- | --- | --- | --- | --- | --- | --- | --- |
| **Predictor** | **df** | **SS** | **R2** | **F** | **p** |  | **df** | **SS** | **R2** | **F** | **p** |
| **(a) Jaccard** |  |  |  |  |  |  | |  |  |  |  |
| Total dose (mGy/s) |  |  |  |  |  |  | 1 | 0.457 | 0.013 | 2.001 | 0.068 |
| Time |  |  |  |  |  |  | 2 | 1.776 | 0.050 | 3.886 | <0.001 |
| Sex | 1 | 0.266 | 0.008 | 1.168 | <0.001 |  | 1 | 0.264 | 0.007 | 1.154 | 0.001 |
| Body Mass | 1 | 0.342 | 0.010 | 1.502 | 0.405 |  | 1 | 0.343 | 0.010 | 1.500 | 0.394 |
| TotaldosemGyS:Time | 2 | 0.534 | 0.015 | 1.172 | 0.176 |  | |  |  |  |  |
| Residual | 138 | 31.451 | 0.887 |  |  |  | 140 | 31.986 | 0.902 |  |  |
| Total | 145 | 35.458 | 1.000 |  |  |  | 145 | 35.458 | 1.000 |  |  |
| **(b) Bray-Curtis** |  |  |  |  |  |  | |  |  |  |  |
| Total dose (mGy/s) |  |  |  |  |  |  | 1 | 0.330 | 0.011 | 1.668 | 0.314 |
| Time |  |  |  |  |  |  | 2 | 1.754 | 0.056 | 4.437 | <0.001 |
| Sex | 1 | 0.144 | 0.005 | 0.732 | 0.013 |  | 1 | 0.142 | 0.005 | 0.719 | 0.022 |
| Body Mass | 1 | 0.407 | 0.013 | 2.066 | 0.286 |  | 1 | 0.410 | 0.013 | 2.073 | 0.243 |
| TotaldosemGyS:Time | 2 | 0.490 | 0.016 | 1.244 | 0.583 |  | |  |  |  |  |
| Residual | 138 | 27.180 | 0.868 |  |  |  | 140 | 27.670 | 0.883 |  |  |
| Total | 145 | 31.321 | 1.000 |  |  |  | 145 | 31.321 | 1.000 |  |  |
| **(c) unweighted UniFrac** |  |  |  |  |  |  | |  |  |  |  |
| Total dose (mGy/s) |  |  |  |  |  |  | 1 | 0.301 | 0.015 | 2.308 | 0.028 |
| Time |  |  |  |  |  |  | 2 | 1.201 | 0.058 | 4.604 | <0.001 |
| Sex | 1 | 0.132 | 0.006 | 1.016 | <0.001 |  | 1 | 0.129 | 0.006 | 0.992 | <0.001 |
| Body Mass | 1 | 0.205 | 0.010 | 1.573 | 0.315 |  | 1 | 0.206 | 0.010 | 1.577 | 0.273 |
| TotaldosemGyS:Time | 2 | 0.305 | 0.015 | 1.172 | 0.213 |  | |  |  |  |  |
| Residual | 138 | 17.953 | 0.873 |  |  |  | 140 | 18.258 | 0.888 |  |  |
| Total | 145 | 20.561 | 1.000 |  |  |  | 145 | 20.561 | 1.000 |  |  |
| **(d) weighted UniFrac** |  |  |  |  |  |  | |  |  |  |  |
| Total dose (mGy/s) |  |  |  |  |  |  | 1 | 0.072 | 0.009 | 1.426 | 0.061 |
| Time |  |  |  |  |  |  | 2 | 0.556 | 0.068 | 5.470 | <0.001 |
| Sex | 1 | 0.038 | 0.005 | 0.745 | 0.044 |  | 1 | 0.034 | 0.004 | 0.671 | 0.043 |
| Body Mass | 1 | 0.098 | 0.012 | 1.947 | 0.380 |  | 1 | 0.100 | 0.012 | 1.973 | 0.283 |
| TotaldosemGyS:Time | 2 | 0.131 | 0.016 | 1.292 | 0.679 |  | |  |  |  |  |
| Residual | 138 | 6.982 | 0.852 |  |  |  | 140 | 7.113 | 0.868 |  |  |
| Total | 145 | 8.193 | 1.000 |  |  |  | 145 | 8.193 | 1.000 |  |  |

Supplementary Table 5a. Statistical significance of differences in dispersion in beta diversity of gut bacteria from the bank vole (*Clethrionomys glareolus*) inhabiting contaminated and uncontaminated areas (July) or field enclosures (August and October) located within the Chornobyl Exclusion Zone (CEZ), Ukraine. *df*, degrees of freedom; *SS*, sum of squares; *MS* mean square; *F*, test statistic; *p*, statistical significance. PCoA visualisations of beta diversity are given in Figure 3 (Jaccard’s dissimilarity) and Supplementary Figures 2a, b, c (all other metrics).

| distance metric | time | *df* | *SS* | *MS* | *F* | *p* |
| --- | --- | --- | --- | --- | --- | --- |
| Jaccard's dissimilarity | July | 1 | 0.00043 | 0.00042 | 0.17 | 0.676 |
|  | August | 1 | 0.01168 | 0.01168 | 3.02 | 0.087 |
|  | October | 1 | 0.00137 | 0.00137 | 1.10 | 0.289 |
| Bray-Curtis | July | 1 | 0.02300 | 0.02300 | 5.25 | 0.026 |
|  | August | 1 | 0.02468 | 0.02468 | 2.75 | 0.107 |
|  | October | 1 | 0.00363 | 0.00363 | 1.06 | 0.306 |
| unweighted UniFrac | July | 1 | 0.00137 | 0.00137 | 0.75 | 0.414 |
|  | August | 1 | 0.00286 | 0.00286 | 1.35 | 0.248 |
|  | October | 1 | 0.00734 | 0.00734 | 5.92 | 0.012 |
| weighted UniFrac | July | 1 | 0.00579 | 0.00579 | 0.54 | 0.490 |
|  | August | 1 | 0.00232 | 0.00232 | 0.32 | 0.582 |
|  | October | 1 | 0.00009 | 0.00010 | 0.04 | 0.846 |

Supplementary Table 5b. Statistical significance of differences in dispersion in beta diversity of gut bacteria from the bank vole (*Clethrionomys glareolus*) inhabiting contaminated and uncontaminated areas (July) or field enclosures (August and October) located within the Chornobyl Exclusion Zone (CEZ), Ukraine. *df*, degrees of freedom; *SS*, sum of squares; *MS* mean square; *F*, test statistic; *p*, statistical significance. PCoA visualisations of beta diversity are given in Figure 3 (Jaccard’s dissimilarity) and Supplementary Figures 2a, b, c (all other metrics).

| **Distance metric** | **Factor** | **Source** | **df** | **SS** | **MS** | **F** | **p** |
| --- | --- | --- | --- | --- | --- | --- | --- |
| **Jaccard** | Treatment | groups | 1 | 0.005 | 0.005 | 3.954 | 0.049 |
|  |  | residuals | 144 | 0.179 | 0.001 |  |  |
|  | Time | groups | 2 | 0.011 | 0.005 | 3.026 | 0.052 |
|  |  | residuals | 143 | 0.256 | 0.002 |  |  |
|  | Treatment:Time | groups | 5 | 0.028 | 0.006 | 3.006 | 0.013 |
|  |  | residuals | 140 | 0.262 | 0.002 |  |  |
| **Bray-Curtis** | Treatment | groups | 1 | 0.026 | 0.026 | 5.974 | 0.016 |
|  |  | residuals | 144 | 0.621 | 0.004 |  |  |
|  | Time | groups | 2 | 0.012 | 0.006 | 1.067 | 0.347 |
|  |  | residuals | 143 | 0.825 | 0.006 |  |  |
|  | Treatment:Time | groups | 5 | 0.062 | 0.012 | 2.239 | 0.054 |
|  |  | residuals | 140 | 0.781 | 0.006 |  |  |
| **UniFrac** | Treatment | groups | 1 | 0.002 | 0.002 | 1.328 | 0.251 |
|  |  | residuals | 144 | 0.258 | 0.002 |  |  |
|  | Time | groups | 2 | 0.037 | 0.019 | 11.550 | <0.001 |
|  |  | residuals | 143 | 0.229 | 0.002 |  |  |
|  | Treatment:Time | groups | 5 | 0.053 | 0.011 | 6.280 | <0.001 |
|  |  | residuals | 140 | 0.236 | 0.002 |  |  |
| **weighted UniFrac** | Treatment | groups | 1 | 0.005 | 0.005 | 0.780 | 0.379 |
|  |  | residuals | 144 | 0.906 | 0.006 |  |  |
|  | Time | groups | 2 | 0.013 | 0.006 | 0.955 | 0.387 |
|  |  | residuals | 143 | 0.944 | 0.007 |  |  |
|  | Treatment:Time | groups | 5 | 0.023 | 0.005 | 0.648 | 0.663 |
|  |  | residuals | 140 | 0.984 | 0.007 |  |  |

Supplementary Table 6a. Outcomes from generalised linear mixed models (GLMMs) testing the effects of Treatment (uncontaminated or contaminated habitat) and Time interval (July-August and Augst-October) on mean temporal changes in beta diversity of gut bacteria of bank voles (*Clethrionomys glareolus*) inhabiting contaminated and uncontaminated areas and field enclosures located within the Chornobyl Exclusion Zone (CEZ), Ukraine. Models were fitted for each metric of beta diversity, with Treatment, Time interval, and their interaction as fixed effects (and individual identity and sample location as random effects). (a) Jaccard’s metric, (b) Bray-Curtis index, (c) UniFrac metric, and (d) weighted UniFrac metric. Est., estimate; SE, standard Error; CI lower/CI upper, lower and upper confidence intervals; *p*, statistical significance.

| **Predictor** | **Est.** | **SE** | **CI lower** | **CI upper** | ***p*** |
| --- | --- | --- | --- | --- | --- |
| **(a) Jaccard** | | | | | |
| (Intercept) | 0.686 | 0.014 | 0.658 | 0.715 | <0.001 |
| Treatment - uncontaminated | -0.013 | 0.020 | -0.052 | 0.027 | 0.531 |
| Time interval - Aug-Oct | 0.023 | 0.024 | -0.024 | 0.071 | 0.333 |
| Treatment - uncontaminated:time interval - Aug-Oct | 0.005 | 0.037 | -0.067 | 0.077 | 0.895 |
| marginal R² | 0.054 | |  |  |  |
| conditional R² | 0.054 | |  |  |  |
| **(b) Bray Curtis** |  | |  |  |  |
| (Intercept) | 0.650 | 0.029 | 0.593 | 0.707 | <0.001 |
| Treatment - uncontaminated | -0.051 | 0.041 | -0.131 | 0.028 | 0.206 |
| Time interval - Aug-Oct | 0.003 | 0.049 | -0.092 | 0.099 | 0.945 |
| Treatment - uncontaminated:time interval - Aug-Oct | 0.097 | 0.074 | -0.048 | 0.242 | 0.188 |
| marginal R² | 0.077 | |  |  |  |
| conditional R² | 0.077 | |  |  |  |
| **(c) UniFrac** |  | |  |  |  |
| (Intercept) | 0.523 | 0.015 | 0.494 | 0.552 | <0.001 |
| Treatment - uncontaminated | -0.008 | 0.021 | -0.048 | 0.033 | 0.706 |
| Time interval - Aug-Oct | 0.013 | 0.025 | -0.035 | 0.061 | 0.598 |
| Treatment - uncontaminated:time interval - Aug-Oct | 0.020 | 0.037 | -0.054 | 0.093 | 0.595 |
| marginal R² | 0.035 | |  |  |  |
| conditional R² | 0.035 | |  |  |  |
| **(d) weighted UniFrac** |  | |  |  |  |
| (Intercept) | 0.355 | 0.059 | 0.240 | 0.470 | <0.001 |
| Treatment - uncontaminated | -0.026 | 0.079 | -0.180 | 0.129 | 0.746 |
| Time interval - Aug-Oct | -0.064 | 0.091 | -0.242 | 0.114 | 0.478 |
| Treatment - uncontaminated:time interval - Aug-Oct | 0.076 | 0.115 | -0.149 | 0.302 | 0.507 |
| marginal R² | 0.029 | |  |  |  |
| conditional R² | 0.857 | |  |  |  |

Supplementary Table 6b. Outcomes from generalised linear mixed models (GLMMs) testing the effects of Treatment (uncontaminated or contaminated habitat) and Time interval (July-August and Augst-October) on the dispersion of temporal changes (deviation of observations from the group mean) in beta diversity of gut bacteria of bank voles (*Clethrionomys glareolus*) inhabiting contaminated and uncontaminated areas and field enclosures located within the Chornobyl Exclusion Zone (CEZ), Ukraine. Models were fitted for each metric of beta diversity, with Treatment, Time interval, and their interaction as fixed effects (and individual identity and sample location as random effects). (a) Jaccard’s metric, (b) Bray-Curtis index, (c) UniFrac metric, and (d) weighted UniFrac metric. Est., estimate; SE, standard Error; CI lower/CI upper, lower and upper confidence intervals; *p*, statistical significance.

| **Predictor** | **Est.** | **SE** | **CI lower** | **CI upper** | **p** |
| --- | --- | --- | --- | --- | --- |
| **(a) Jaccard** | | | | | |
| (Intercept) | 0.040 | 0.008 | 0.024 | 0.057 | <0.001 |
| Treatment - uncontaminated | 0.021 | 0.012 | -0.002 | 0.043 | 0.074 |
| Time interval - Aug-Oct | -0.006 | 0.014 | -0.033 | 0.020 | 0.633 |
| Treatment - uncontaminated:time interval - Aug-Oct | -0.017 | 0.022 | -0.060 | 0.025 | 0.423 |
| marginal R² | 0.106 | |  |  |  |
| conditional R² | 0.195 | |  |  |  |
| **(b) Bray Curtis** |  | |  |  |  |
| (Intercept) | 0.096 | 0.019 | 0.060 | 0.133 | <0.001 |
| Treatment - uncontaminated | 0.005 | 0.026 | -0.046 | 0.056 | 0.852 |
| Time interval - Aug-Oct | -0.033 | 0.031 | -0.094 | 0.029 | 0.296 |
| Treatment - uncontaminated:time interval - Aug-Oct | -0.004 | 0.047 | -0.097 | 0.089 | 0.939 |
| marginal R² | 0.045 | |  |  |  |
| conditional R² | 0.045 | |  |  |  |
| **(c) UniFrac** |  | |  |  |  |
| (Intercept) | 0.050 | 0.008 | 0.034 | 0.066 | <0.001 |
| Treatment - uncontaminated | 0.000 | 0.011 | -0.023 | 0.022 | 0.966 |
| Time interval - Aug-Oct | -0.014 | 0.014 | -0.040 | 0.013 | 0.314 |
| Treatment - uncontaminated:time interval - Aug-Oct | 0.030 | 0.021 | -0.010 | 0.071 | 0.146 |
| marginal R² | 0.059 | |  |  |  |
| conditional R² | 0.059 | |  |  |  |
| **(d) weighted UniFrac** |  | |  |  |  |
| (Intercept) | 0.115 | 0.027 | 0.063 | 0.168 | <0.001 |
| Treatment - uncontaminated | -0.020 | 0.036 | -0.091 | 0.051 | 0.578 |
| Time interval - Aug-Oct | -0.043 | 0.041 | -0.123 | 0.037 | 0.289 |
| Treatment - uncontaminated:time interval - Aug-Oct | 0.042 | 0.060 | -0.075 | 0.159 | 0.483 |
| marginal R² | 0.037 | |  |  |  |
| conditional R² | 0.143 | |  |  |  |

Supplementary Table 7. Relative abundance of the dominant phyla, orders, and families of gut bacteria from bank voles (*Clethrionomys glareolus*) inhabiting contaminated and uncontaminated areas (July) or field enclosures (August and October) located within the Chornobyl Exclusion Zone (CEZ), Ukraine.

|  | contaminated | | | uncontaminated | | |  |
| --- | --- | --- | --- | --- | --- | --- | --- |
|  | July | August | October | July | August | October | average |
| ***Phylum*** |  |  |  |  |  |  |  |
| Actinobacteriota | 0.030 | 0.040 | 0.019 | 0.023 | 0.030 | 0.017 | 0.027 |
| Bacteroidota (=Bacteroidetes) | 0.490 | 0.492 | 0.426 | 0.458 | 0.520 | 0.526 | 0.485 |
| Desulfobacterota | 0.030 | 0.068 | 0.035 | 0.025 | 0.041 | 0.038 | 0.039 |
| Bacillota (=Firmicutes) | 0.433 | 0.385 | 0.497 | 0.479 | 0.379 | 0.402 | 0.429 |
| Spirochaetota | 0.010 | 0.011 | 0.013 | 0.000 | 0.025 | 0.000 | 0.010 |
| <1% abundance features | 0.007 | 0.004 | 0.010 | 0.015 | 0.005 | 0.017 | 0.010 |
| ***Order*** |  |  |  |  |  |  |  |
| Bacteroidales | 0.480 | 0.488 | 0.415 | 0.452 | 0.516 | 0.512 | 0.477 |
| Christensenellales | 0.064 | 0.033 | 0.038 | 0.054 | 0.042 | 0.037 | 0.045 |
| Coriobacteriales | 0.029 | 0.040 | 0.000 | 0.023 | 0.030 | 0.000 | 0.020 |
| Desulfovibrionales | 0.030 | 0.068 | 0.035 | 0.025 | 0.041 | 0.038 | 0.039 |
| Lachnospirales | 0.082 | 0.045 | 0.154 | 0.070 | 0.022 | 0.113 | 0.081 |
| Lactobacillales | 0.208 | 0.188 | 0.155 | 0.286 | 0.281 | 0.118 | 0.206 |
| Oscillospirales | 0.050 | 0.037 | 0.122 | 0.048 | 0.000 | 0.102 | 0.060 |
| Erysipelotrichales | 0.000 | 0.072 | 0.000 | 0.000 | 0.000 | 0.000 | 0.012 |
| Spirochaetales | 0.000 | 0.000 | 0.000 | 0.000 | 0.025 | 0.000 | 0.004 |
| Clostridia_vadin BB60_group | 0.000 | 0.000 | 0.000 | 0.000 | 0.000 | 0.021 | 0.003 |
| <2% abundance features | 0.057 | 0.029 | 0.082 | 0.041 | 0.044 | 0.060 | 0.052 |
| ***Family*** |  |  |  |  |  |  |  |
| Christensenellaceae | 0.064 | 0.033 | 0.038 | 0.054 | 0.042 | 0.037 | 0.045 |
| Desulfovibrionaceae | 0.030 | 0.068 | 0.035 | 0.025 | 0.041 | 0.038 | 0.039 |
| Eggerthellaceae | 0.024 | 0.032 | 0.000 | 0.000 | 0.024 | 0.000 | 0.013 |
| Lachnospiraceae | 0.082 | 0.045 | 0.154 | 0.070 | 0.022 | 0.113 | 0.081 |
| Lactobacillaceae | 0.206 | 0.185 | 0.154 | 0.284 | 0.279 | 0.114 | 0.204 |
| Muribaculaceae | 0.470 | 0.480 | 0.393 | 0.436 | 0.504 | 0.486 | 0.462 |
| Oscillospiraceae | 0.029 | 0.024 | 0.085 | 0.031 | 0.000 | 0.070 | 0.040 |
| Erysipelotrichaceae | 0.000 | 0.072 | 0.000 | 0.000 | 0.000 | 0.000 | 0.012 |
| Ruminococcaceae | 0.000 | 0.000 | 0.031 | 0.000 | 0.000 | 0.027 | 0.010 |
| Spirochaetaceae | 0.000 | 0.000 | 0.000 | 0.000 | 0.025 | 0.000 | 0.004 |
| Clostridia_vadinBB60_group | 0.000 | 0.000 | 0.000 | 0.000 | 0.000 | 0.021 | 0.003 |
| <2% abundance features | 0.097 | 0.061 | 0.111 | 0.100 | 0.064 | 0.094 | 0.088 |

Supplementary Table 8. Results of ANCOM-BC2 analysis showing estimated coefficients for differential abundance of gut bacterial taxa in relation to radiation dose and time from bank voles (*Clethrionomys glareolus*) inhabiting contaminated and uncontaminated areas located within the Chornobyl Exclusion Zone (CEZ), Ukraine. *lfc*, log fold change (mGy^-1^); *se*, standard error of lfc; *W*, test statistic; *q*, statistical significance adjusted for multiple testing.

|  | ***lfc*** |  |  |  |  |  | ***se*** |  |  |  |  |  |
| --- | --- | --- | --- | --- | --- | --- | --- | --- | --- | --- | --- | --- |
| **taxon** | **intercept** | **Dose** | **Time-Aug** | **Time-Oct** | **Dose:Time-Aug** | **Dose:Time-Oct** | **intercept** | **Dose** | **Time-Aug** | **Time-Oct** | **Dose:Time-Aug** | **Dose:Time-Oct** |
| GCA-900066575 | -32.97 | 1.55 | 46.62 | 32.34 | -1.66 | -1.55 | 28.43 | 0.01 | 26.95 | 27.12 | 0.01 | 0.01 |
| c_Alphaproteobacteria | 0.33 | -0.13 | 0.49 | -0.81 | 0.14 | 0.13 | 27.57 | 0.01 | 26.13 | 26.30 | 0.01 | 0.01 |
| Campylobacter | 20.93 | -37.78 | 0.00 | -22.11 | 0.00 | 36.55 | 30.86 | 0.01 | 29.25 | 29.45 | 0.01 | 0.01 |
| Jeotgalicoccus | -59.64 | 2.35 | 59.49 | -303.67 | -2.19 | NA | 30.66 | 0.01 | 29.06 | 29.26 | 0.01 | NA |
| [Eubacterium]_ruminantium_group | -4.26 | 3.99 | 9.45 | 5.27 | -4.04 | -3.99 | 30.07 | 0.01 | 28.50 | 28.69 | 0.01 | 0.01 |
| Lachnospiraceae_UCG-006 | 1.25 | -3.17 | 697.33 | -1.97 | NA | 3.05 | 30.27 | 0.01 | 28.71 | 28.90 | NA | 0.03 |
| o_Rickettsiales | -0.50 | 0.05 | 1.86 | 0.25 | -0.04 | -0.04 | 26.90 | 0.01 | 25.53 | 25.68 | 0.05 | 0.03 |
| o_Oscillospirales | 9.06 | -11.53 | 1503.89 | -9.94 | NA | 11.62 | 30.66 | 0.01 | 29.08 | 29.27 | NA | 0.03 |
| Pygmaiobacter | 0.42 | 0.03 | 0.48 | 0.28 | -0.03 | -0.03 | 28.21 | 0.01 | 26.74 | 26.92 | 0.01 | 0.01 |
| Corynebacterium | -0.95 | 1.46 | 0.48 | -0.98 | NA | NA | 31.05 | 0.01 | 29.43 | 29.63 | NA | NA |
| Erysipelotrichaceae | 1.78 | 0.39 | -1.34 | -2.50 | NA | -0.39 | 30.57 | 0.01 | 28.97 | 29.17 | NA | 0.01 |
| Enterococcus | 0.54 | -0.03 | 0.63 | 0.32 | -0.04 | NA | 30.27 | 0.01 | 28.69 | 28.88 | 0.01 | NA |
| [Clostridium]_methylpentosum_group | -1.38 | 0.05 | 2.70 | -12.88 | -0.04 | NA | 30.37 | 0.01 | 28.78 | 28.98 | 0.01 | NA |
| Ureaplasma | -0.29 | 0.04 | 0.00 | -0.25 | 0.00 | NA | 30.86 | 0.01 | 29.25 | 29.45 | 0.01 | NA |
| f_Christensenellaceae | -0.69 | 0.01 | 1.81 | 0.20 | -0.01 | -0.01 | 12.53 | 0.01 | 11.98 | 11.98 | 0.06 | 0.03 |
| f_Actinomycetaceae | -0.60 | -0.01 | 1.51 | -1.93 | 0.00 | 0.01 | 29.87 | 0.01 | 28.31 | 28.50 | 0.01 | 0.01 |
| Brevibacterium | 0.68 | 0.00 | 1.35 | 0.00 | NA | 0.00 | 31.05 | 0.01 | 29.43 | 29.63 | NA | 0.01 |
| Brachybacterium | -2.76 | 2.53 | 0.00 | 2.53 | 0.00 | -2.53 | 31.15 | 0.01 | 29.52 | 29.72 | 0.01 | 0.01 |
| f_Microbacteriaceae | NA | NA | NA | NA | NA | NA | NA | NA | NA | NA | NA | NA |
| Glutamicibacter | -0.60 | -0.01 | 1.70 | 0.24 | -0.01 | NA | 30.76 | 0.01 | 29.15 | 29.35 | 0.01 | NA |
| Streptomyces | 0.80 | -0.01 | NA | 0.00 | NA | 0.00 | 31.44 | 0.01 | NA | 30.00 | NA | 0.01 |
| f_Atopobiaceae | -26.47 | 35.13 | -4526.10 | 0.00 | NA | 0.00 | 31.34 | 0.01 | 29.70 | 29.91 | NA | 0.01 |
| f_Coriobacteriales_Incertae_Sedis | 1.03 | 0.00 | 0.88 | -1.23 | -0.01 | -0.01 | 14.95 | 0.01 | 14.26 | 14.29 | 0.06 | 0.03 |
| DNF00809 | 1.31 | 0.00 | 0.00 | -2.13 | 0.00 | NA | 31.34 | 0.01 | 29.70 | 29.91 | 0.01 | NA |
| Enterorhabdus | 1.30 | 0.00 | 0.75 | -1.80 | 0.00 | 0.00 | 10.09 | 0.01 | 9.70 | 9.67 | 0.06 | 0.03 |
| f_Eggerthellaceae | 1.25 | 0.00 | 0.18 | -1.64 | 0.00 | 0.00 | 9.12 | 0.00 | 8.80 | 8.76 | 0.06 | 0.03 |
| f_Marinifilaceae | -0.16 | 0.00 | 0.68 | 0.45 | 0.00 | 0.00 | 17.38 | 0.01 | 16.51 | 16.59 | 0.01 | 0.01 |
| Odoribacter | -0.95 | 0.00 | 1.20 | 0.92 | 0.00 | 0.00 | 20.56 | 0.01 | 19.51 | 19.62 | 0.01 | 0.01 |
| f_Muribaculaceae | -0.92 | 0.00 | 1.99 | 1.05 | -0.01 | 0.00 | 22.65 | 0.01 | 21.52 | 21.63 | 0.05 | 0.03 |
| Muribaculaceae | -0.01 | 0.00 | 1.63 | 0.01 | 0.00 | 0.00 | 8.00 | 0.00 | 7.77 | 7.71 | 0.06 | 0.03 |
| Alistipes | 0.38 | 0.00 | -0.01 | 0.20 | 0.00 | 0.00 | 14.54 | 0.01 | 13.87 | 13.90 | 0.05 | 0.03 |
| f_Rikenellaceae | 1.61 | 0.00 | -0.34 | -1.98 | 0.00 | 0.00 | 24.57 | 0.01 | 23.30 | 23.44 | 0.01 | 0.01 |
| Rikenella | -1.04 | 0.00 | 1.30 | 1.14 | 0.01 | 0.00 | 23.05 | 0.01 | 21.86 | 21.99 | 0.01 | 0.01 |
| Rs-E47_termite_group | 2.11 | 0.00 | -0.26 | -1.21 | 0.00 | 0.00 | 17.55 | 0.01 | 16.67 | 16.75 | 0.01 | 0.01 |
| c_Bacteroidia | 0.53 | -0.01 | -0.28 | -0.72 | 0.01 | 0.01 | 21.42 | 0.01 | 20.33 | 20.44 | 0.01 | 0.01 |
| Gastranaerophilales | -0.23 | 0.00 | 0.68 | 0.87 | 0.00 | 0.00 | 19.97 | 0.01 | 18.98 | 19.07 | 0.06 | 0.03 |
| Mucispirillum | -0.54 | -0.01 | -5.47 | 0.29 | 0.03 | 0.00 | 29.87 | 0.01 | 28.31 | 28.50 | 0.01 | 0.01 |
| Bilophila | 0.54 | 0.00 | -0.62 | -1.04 | 0.01 | 0.00 | 27.13 | 0.01 | 25.72 | 25.88 | 0.01 | 0.01 |
| Desulfovibrio | 1.99 | 0.00 | -0.08 | -2.32 | 0.00 | 0.00 | 9.13 | 0.01 | 8.81 | 8.77 | 0.06 | 0.03 |
| f_Desulfovibrionaceae | 1.35 | 0.00 | 0.05 | -1.49 | 0.01 | 0.00 | 18.88 | 0.01 | 17.96 | 18.03 | 0.06 | 0.03 |
| c_Bacilli | NA | NA | NA | NA | NA | NA | NA | NA | NA | NA | NA | NA |
| Allobaculum | 0.67 | 0.00 | -0.28 | -2.19 | 0.01 | 0.01 | 27.78 | 0.01 | 26.37 | 26.53 | 0.06 | 0.03 |
| f_Erysipelotrichaceae | 0.28 | 0.00 | 0.43 | 0.29 | 0.65 | NA | 29.87 | 0.01 | 28.31 | 28.50 | 0.01 | NA |
| Aerococcus | 35.85 | -1.35 | -33.07 | 0.00 | NA | 0.00 | 31.05 | 0.01 | 29.43 | 29.63 | NA | 0.01 |
| f_Lactobacillaceae | 2.48 | -1.99 | -2.85 | 0.00 | 1.96 | 0.00 | 31.05 | 0.01 | 29.43 | 29.63 | 0.01 | 0.01 |
| Lactobacillus | 0.71 | 0.00 | 1.58 | -1.41 | 0.00 | 0.01 | 9.47 | 0.01 | 9.12 | 9.09 | 0.05 | 0.03 |
| o_Lactobacillales | -0.36 | -0.03 | 1.86 | 5.20 | NA | NA | 30.96 | 0.01 | 29.34 | 29.54 | NA | NA |
| Lactococcus | -0.49 | 0.03 | 0.00 | 0.29 | 0.00 | NA | 31.34 | 0.01 | 29.70 | 29.91 | 0.01 | NA |
| Streptococcus | 1.65 | 0.00 | -0.13 | -1.94 | -0.01 | -0.01 | 17.72 | 0.01 | 16.87 | 16.93 | 0.06 | 0.03 |
| Mycoplasma | -1.04 | 0.00 | 0.34 | 0.03 | 0.01 | 0.00 | 29.77 | 0.01 | 28.22 | 28.41 | 0.01 | 0.01 |
| RF39 | 1.16 | 0.00 | -0.24 | -1.30 | 0.00 | 0.00 | 23.56 | 0.01 | 22.35 | 22.49 | 0.01 | 0.01 |
| c_Clostridia | 1.25 | 0.00 | 0.38 | -2.21 | -0.02 | -0.01 | 26.90 | 0.01 | 25.51 | 25.67 | 0.01 | 0.01 |
| Christensenella | 1.54 | -0.01 | 0.00 | -1.30 | 0.00 | NA | 31.25 | 0.01 | 29.61 | 29.82 | 0.01 | NA |
| Christensenellaceae_R-7_group | NA | NA | NA | NA | NA | NA | NA | NA | NA | NA | NA | NA |
| Clostridia_UCG-014 | 1.29 | 0.00 | -0.23 | -1.09 | 0.00 | 0.00 | 16.12 | 0.01 | 15.32 | 15.38 | 0.01 | 0.01 |
| Clostridia_vadinBB60_group | 0.11 | 0.00 | -0.04 | 1.04 | 0.01 | 0.00 | 13.23 | 0.01 | 12.64 | 12.65 | 0.06 | 0.03 |
| Candidatus_Arthromitus | 0.04 | 0.01 | 1.40 | -0.49 | -0.01 | 0.00 | 27.68 | 0.01 | 26.24 | 26.41 | 0.01 | 0.01 |
| Anaerofustis | -0.31 | 0.01 | NA | 0.00 | NA | 0.00 | 31.44 | 0.01 | NA | 30.00 | NA | 0.01 |
| [Eubacterium]_ventriosum_group | -3.08 | -0.02 | 1.40 | 3.06 | NA | NA | 30.86 | 0.01 | 29.25 | 29.45 | NA | NA |
| [Eubacterium]_xylanophilum_group | -0.08 | 0.00 | 0.39 | -0.34 | 0.00 | 0.00 | 20.71 | 0.01 | 19.68 | 19.78 | 0.06 | 0.03 |
| A2 | 0.63 | -0.01 | 0.82 | -0.31 | 0.01 | 0.01 | 25.64 | 0.01 | 24.32 | 24.47 | 0.01 | 0.01 |
| Acetatifactor | 1.74 | 0.01 | -1.64 | -1.43 | 0.00 | -0.01 | 23.69 | 0.01 | 22.47 | 22.61 | 0.01 | 0.01 |
| Anaerostipes | 0.17 | 0.00 | 0.97 | 0.39 | -0.05 | -0.01 | 29.05 | 0.01 | 27.54 | 27.72 | 0.01 | 0.01 |
| ASF356 | 1.70 | 0.00 | -1.43 | -1.10 | NA | 0.00 | 27.13 | 0.01 | 25.72 | 25.89 | NA | 0.01 |
| Blautia | 1.15 | 0.00 | 0.60 | -0.67 | 0.00 | 0.00 | 27.46 | 0.01 | 26.03 | 26.20 | 0.01 | 0.01 |
| Coprococcus | -0.24 | 0.00 | 0.00 | -0.90 | 0.00 | NA | 31.25 | 0.01 | 29.61 | 29.82 | 0.01 | NA |
| Dorea | -0.76 | -0.01 | 1.31 | 1.72 | 0.01 | 0.01 | 27.46 | 0.01 | 26.03 | 26.20 | 0.01 | 0.01 |
| f_Lachnospiraceae | 1.94 | 0.00 | -1.70 | -1.40 | 0.00 | 0.00 | 8.78 | 0.00 | 8.48 | 8.43 | 0.06 | 0.03 |
| Lachnoclostridium | -0.06 | 0.01 | 0.03 | 0.10 | -0.01 | -0.01 | 22.65 | 0.01 | 21.49 | 21.62 | 0.01 | 0.01 |
| Lachnospiraceae_FCS020_group | -0.17 | 0.00 | 0.00 | 0.21 | 0.00 | 0.00 | 28.43 | 0.01 | 26.95 | 27.12 | 0.01 | 0.01 |
| Lachnospiraceae_NC2004_group | -0.09 | 0.00 | 2.17 | -0.07 | NA | 0.00 | 29.97 | 0.01 | 28.41 | 28.60 | NA | 0.01 |
| Lachnospiraceae_NK4A136_group | 0.51 | 0.00 | 0.11 | -0.12 | 0.00 | 0.00 | 11.50 | 0.01 | 11.03 | 11.02 | 0.06 | 0.03 |
| Lachnospiraceae_NK4B4_group | 0.82 | 0.00 | 0.00 | -0.10 | 0.00 | NA | 31.15 | 0.01 | 29.52 | 29.72 | 0.01 | NA |
| Lachnospiraceae_UCG-001 | -0.31 | 0.00 | 1.15 | 0.70 | -0.01 | 0.00 | 25.53 | 0.01 | 24.20 | 24.36 | 0.01 | 0.01 |
| Lachnospiraceae_UCG-008 | -1.94 | 2.13 | 0.00 | NA | 0.00 | NA | 31.44 | 0.01 | 29.79 | NA | 0.01 | NA |
| Lachnospiraceae_UCG-010 | 0.67 | 0.00 | 0.00 | -0.44 | 0.00 | NA | 30.57 | 0.01 | 28.97 | 29.17 | 0.01 | NA |
| Roseburia | 1.91 | 0.00 | -1.69 | -1.81 | 0.00 | 0.00 | 17.21 | 0.01 | 16.34 | 16.42 | 0.01 | 0.01 |
| Tyzzerella | 1.87 | 0.00 | -1.53 | -1.92 | 0.00 | 0.00 | 24.69 | 0.01 | 23.41 | 23.56 | 0.01 | 0.01 |
| Monoglobus | 1.60 | 0.00 | -1.02 | -1.70 | 0.00 | 0.00 | 20.85 | 0.01 | 19.79 | 19.90 | 0.01 | 0.01 |
| [Eubacterium]_coprostanoligenes_group | 0.67 | 0.00 | 0.13 | -0.40 | 0.00 | 0.00 | 21.00 | 0.01 | 19.92 | 20.03 | 0.01 | 0.01 |
| Butyricicoccus | 0.97 | 0.01 | -3.71 | -1.43 | NA | -0.02 | 30.07 | 0.01 | 28.50 | 28.69 | NA | 0.01 |
| f_Butyricicoccaceae | -1.11 | 0.00 | 2.72 | 0.49 | -0.02 | -0.01 | 29.67 | 0.01 | 28.12 | 28.31 | 0.01 | 0.01 |
| UCG-009 | -1.26 | 0.00 | 2.38 | 1.17 | -0.02 | 0.01 | 29.16 | 0.01 | 27.64 | 27.82 | 0.01 | 0.01 |
| Colidextribacter | 1.51 | 0.00 | -1.42 | -1.00 | 0.00 | 0.00 | 17.89 | 0.01 | 16.99 | 17.07 | 0.01 | 0.01 |
| f_Oscillospiraceae | 2.47 | 0.00 | -2.56 | -1.51 | 0.00 | 0.00 | 9.46 | 0.01 | 9.12 | 9.08 | 0.06 | 0.03 |
| Intestinimonas | 1.28 | 0.00 | 0.00 | -1.54 | 0.00 | NA | 30.66 | 0.01 | 29.06 | 29.26 | 0.01 | NA |
| Oscillibacter | 1.73 | 0.00 | -1.61 | -1.81 | 0.00 | 0.00 | 23.69 | 0.01 | 22.47 | 22.61 | 0.01 | 0.01 |
| Oscillospira | -0.67 | 0.00 | 0.00 | 0.70 | 0.00 | NA | 30.96 | 0.01 | 29.34 | 29.54 | 0.01 | NA |
| Papillibacter | 0.57 | 0.00 | 0.00 | -1.33 | 0.00 | NA | 31.05 | 0.01 | 29.43 | 29.63 | 0.01 | NA |
| UCG-005 | 0.83 | 0.00 | -0.08 | -0.81 | 0.01 | 0.00 | 26.57 | 0.01 | 25.19 | 25.35 | 0.01 | 0.01 |
| V9D2013_group | -0.77 | 0.00 | 0.00 | 0.83 | 0.00 | NA | 31.25 | 0.01 | 29.61 | 29.82 | 0.01 | NA |
| [Eubacterium]_siraeum_group | 1.88 | 0.00 | -0.73 | -2.41 | 0.01 | 0.01 | 21.00 | 0.01 | 19.95 | 20.05 | 0.06 | 0.03 |
| Anaerofilum | NA | NA | NA | NA | NA | NA | NA | NA | NA | NA | NA | NA |
| Anaerotruncus | -1.14 | 0.00 | 0.00 | 1.30 | 0.00 | NA | 31.34 | 0.01 | 29.70 | 29.91 | 0.01 | NA |
| Candidatus_Soleaferrea | -32.16 | 59.20 | -6949.34 | 0.00 | NA | 0.00 | 31.34 | 0.01 | 29.70 | 29.91 | NA | 0.01 |
| Caproiciproducens | -0.20 | 0.00 | 2.60 | 0.00 | NA | 0.00 | 31.34 | 0.01 | 29.70 | 29.91 | NA | 0.01 |
| f_Ruminococcaceae | 1.69 | 0.00 | -0.84 | -0.99 | 0.00 | 0.00 | 13.23 | 0.01 | 12.64 | 12.65 | 0.06 | 0.03 |
| Harryflintia | 13.13 | 0.00 | -12.23 | -3.70 | 0.06 | -0.01 | 29.67 | 0.01 | 28.12 | 28.31 | 0.01 | 0.01 |
| Incertae_Sedis | 0.49 | 0.00 | -0.69 | -0.45 | 0.00 | 0.00 | 21.84 | 0.01 | 20.75 | 20.86 | 0.05 | 0.03 |
| Paludicola | -17.13 | 0.13 | 0.00 | -12.42 | 0.00 | NA | 31.15 | 0.01 | 29.52 | 29.72 | 0.01 | NA |
| Ruminococcaceae | 0.02 | -0.01 | 1.03 | -0.33 | 0.01 | 0.00 | 29.16 | 0.01 | 27.64 | 27.82 | 0.01 | 0.01 |
| Ruminococcus | 3.05 | 0.00 | -2.50 | -2.32 | 0.01 | 0.01 | 16.67 | 0.01 | 15.88 | 15.93 | 0.06 | 0.03 |
| UBA1819 | 0.72 | 0.00 | -0.75 | -0.90 | 0.01 | 0.01 | 18.72 | 0.01 | 17.77 | 17.86 | 0.01 | 0.01 |
| UCG-010 | -0.90 | 0.01 | 1.13 | 1.02 | 0.00 | 0.00 | 21.84 | 0.01 | 20.72 | 20.84 | 0.01 | 0.01 |
| f_Peptococcaceae | 0.38 | 0.00 | 0.45 | 0.02 | 0.00 | 0.00 | 19.81 | 0.01 | 18.81 | 18.91 | 0.01 | 0.01 |
| Peptococcus | 0.23 | 0.00 | -0.55 | -0.69 | NA | 0.00 | 28.21 | 0.01 | 26.74 | 26.92 | NA | 0.01 |
| [Eubacterium]_brachy_group | 1.70 | 0.00 | -0.59 | -1.97 | 0.00 | 0.00 | 20.41 | 0.01 | 19.37 | 19.48 | 0.01 | 0.01 |
| [Eubacterium]_nodatum_group | 0.48 | 0.01 | 0.00 | -3.13 | 0.00 | NA | 30.96 | 0.01 | 29.34 | 29.54 | 0.01 | NA |
| f_Anaerovoracaceae | NA | NA | NA | NA | NA | NA | NA | NA | NA | NA | NA | NA |
| Family_XIII_AD3011_group | 1.63 | 0.00 | -0.85 | -1.68 | 0.00 | 0.00 | 24.69 | 0.01 | 23.44 | 23.57 | 0.06 | 0.03 |
| Family_XIII_UCG-001 | 0.13 | -0.01 | 0.33 | -0.26 | 0.01 | 0.01 | 25.17 | 0.01 | 23.87 | 24.02 | 0.01 | 0.01 |
| o_Peptostreptococcales-Tissierellales | -3.34 | 4.89 | NA | 0.00 | NA | 0.00 | 31.44 | 0.01 | NA | 30.00 | NA | 0.01 |
| Quinella | -0.08 | 0.00 | 0.73 | NA | NA | NA | 31.34 | 0.01 | 29.70 | NA | NA | NA |
| Veillonella | NA | NA | NA | NA | NA | NA | NA | NA | NA | NA | NA | NA |
| p_Firmicutes | NA | NA | NA | NA | NA | NA | NA | NA | NA | NA | NA | NA |
| Candidatus_Saccharimonas | 1.03 | 0.00 | 0.19 | -1.32 | 0.01 | 0.00 | 20.56 | 0.01 | 19.54 | 19.64 | 0.05 | 0.03 |
| f_Paracaedibacteraceae | 2.78 | -0.01 | -0.90 | -2.84 | 0.01 | 0.01 | 25.88 | 0.01 | 24.54 | 24.69 | 0.01 | 0.01 |
| o_Rhodospirillales | 0.75 | -0.01 | -0.01 | -1.14 | 0.02 | 0.01 | 25.88 | 0.01 | 24.54 | 24.69 | 0.01 | 0.01 |
| o_Burkholderiales | 0.99 | 0.00 | 0.01 | -1.79 | 0.00 | 0.00 | 26.57 | 0.01 | 25.19 | 25.35 | 0.01 | 0.01 |
| f_Oxalobacteraceae | 2.60 | 0.00 | -1.79 | -2.50 | 0.01 | 0.00 | 25.76 | 0.01 | 24.43 | 24.58 | 0.01 | 0.01 |
| c_Gammaproteobacteria | 1.41 | 0.01 | 0.44 | -2.34 | -0.02 | -0.01 | 28.74 | 0.01 | 27.27 | 27.44 | 0.05 | 0.03 |
| Rickettsiella | NA | NA | NA | NA | NA | NA | NA | NA | NA | NA | NA | NA |
| Hafnia-Obesumbacterium | 3.31 | -0.12 | -0.72 | 0.00 | NA | 0.00 | 31.05 | 0.01 | 29.43 | 29.63 | NA | 0.01 |
| f_Pasteurellaceae | -1.20 | 0.01 | 8.56 | 1.75 | -0.03 | NA | 30.37 | 0.01 | 28.78 | 28.98 | 0.01 | NA |
| Brachyspira | -0.77 | 0.00 | 1.02 | 0.29 | NA | NA | 30.96 | 0.01 | 29.34 | 29.54 | NA | NA |
| Treponema | -1.22 | 0.01 | 2.27 | -0.10 | -0.01 | 0.00 | 17.56 | 0.01 | 16.67 | 16.75 | 0.01 | 0.01 |
| f_Puniceicoccaceae | -1.14 | -0.04 | 3.62 | 0.00 | NA | 0.00 | 31.34 | 0.01 | 29.70 | 29.91 | NA | 0.01 |
| Citricoccus | 1.19 | 0.00 | NA | 0.00 | NA | 0.00 | 31.44 | 0.01 | NA | 30.00 | NA | 0.01 |
| o_Coriobacteriales | 1.02 | 0.01 | 0.79 | -1.53 | -0.01 | -0.01 | 15.93 | 0.01 | 15.18 | 15.22 | 0.06 | 0.03 |
| o_Bacteroidales | -15.20 | 0.05 | 0.00 | 14.83 | 0.00 | NA | 31.25 | 0.01 | 29.61 | 29.82 | 0.01 | NA |
| Helicobacter | 1.69 | 0.00 | -1.80 | -1.76 | 0.00 | 0.00 | 20.41 | 0.01 | 19.37 | 19.48 | 0.01 | 0.01 |
| Anaeroplasma | 1.24 | -0.01 | -0.78 | -0.57 | 0.02 | 0.00 | 24.69 | 0.01 | 23.44 | 23.57 | 0.05 | 0.03 |
| Carnobacterium | 1.56 | -1.31 | 149.53 | 0.00 | NA | 0.00 | 31.15 | 0.01 | 29.52 | 29.72 | NA | 0.01 |
| Christensenellaceae | -0.02 | 0.00 | 1.54 | -0.18 | 0.00 | 0.00 | 24.93 | 0.01 | 23.64 | 23.79 | 0.01 | 0.01 |
| Ruminiclostridium | -1.23 | 0.09 | 0.00 | -1.47 | 0.00 | NA | 31.05 | 0.01 | 29.43 | 29.63 | 0.01 | NA |
| Tuzzerella | 0.22 | 0.00 | 0.00 | NA | 0.00 | NA | 31.44 | 0.01 | 29.79 | NA | 0.01 | NA |
| NK4A214_group | 1.00 | 0.00 | -1.34 | -1.73 | 0.00 | 0.00 | 23.31 | 0.01 | 22.11 | 22.24 | 0.01 | 0.01 |
| Acinetobacter | -806.42 | 2.76 | 804.88 | 799.43 | NA | NA | 31.25 | 0.01 | 29.61 | 29.82 | NA | NA |

Supplementary Table 8 continued.

|  | **W** |  |  |  |  |  | **q** |  |  |  |  |  |
| --- | --- | --- | --- | --- | --- | --- | --- | --- | --- | --- | --- | --- |
| **taxon** | **intercept** | **Dose** | **Time-Aug** | **Time-Oct** | **Dose:Time-Aug** | **Dose:Time-Oct** | **intercept** | **Dose** | **Time-Aug** | **Time-Oct** | **Dose:Time-Aug** | **Dose:Time-Oct** |
| GCA-900066575 | -1.16 | 160.88 | 1.73 | 1.19 | -261.34 | -231.52 | 1.00 | 0.00 | 1.00 | 1.00 | 0.00 | 0.00 |
| c_Alphaproteobacteria | 0.01 | -14.08 | 0.02 | -0.03 | 22.00 | 20.44 | 1.00 | 0.00 | 1.00 | 1.00 | 0.00 | 0.00 |
| Campylobacter | 0.68 | -3654.10 | 0.00 | -0.75 | 0.00 | 5199.98 | 1.00 | 0.00 | 1.00 | 1.00 | 1.00 | 0.00 |
| Jeotgalicoccus | -1.94 | 228.08 | 2.05 | -10.38 | -329.45 | NA | 1.00 | 0.00 | 1.00 | 0.26 | 0.00 | 1.00 |
| [Eubacterium]_ruminantium_group | -0.14 | 394.43 | 0.33 | 0.18 | -614.90 | -577.62 | 1.00 | 0.00 | 1.00 | 1.00 | 0.00 | 0.00 |
| Lachnospiraceae_UCG-006 | 0.04 | -311.67 | 24.29 | -0.07 | NA | 105.30 | 1.00 | 0.00 | 0.23 | 1.00 | 1.00 | 0.00 |
| o_Rickettsiales | -0.02 | 4.93 | 0.07 | 0.01 | -0.75 | -1.55 | 1.00 | 0.00 | 1.00 | 1.00 | 1.00 | 1.00 |
| o_Oscillospirales | 0.30 | -1121.57 | 51.71 | -0.34 | NA | 407.06 | 1.00 | 0.01 | 0.85 | 1.00 | 1.00 | 0.03 |
| Pygmaiobacter | 0.01 | 3.55 | 0.02 | 0.01 | -4.94 | -4.95 | 1.00 | 0.03 | 1.00 | 1.00 | 0.00 | 0.00 |
| Corynebacterium | -0.03 | 140.05 | 0.02 | -0.03 | NA | NA | 1.00 | 0.06 | 1.00 | 1.00 | 1.00 | 1.00 |
| Erysipelotrichaceae | 0.06 | 38.17 | -0.05 | -0.09 | NA | -56.53 | 1.00 | 0.21 | 1.00 | 1.00 | 1.00 | 0.19 |
| Enterococcus | 0.02 | -3.20 | 0.02 | 0.01 | -5.67 | NA | 1.00 | 0.21 | 1.00 | 1.00 | 0.02 | 1.00 |
| [Clostridium]_methylpentosum_group | -0.05 | 4.78 | 0.09 | -0.44 | -6.56 | NA | 1.00 | 0.44 | 1.00 | 1.00 | 0.18 | 1.00 |
| Ureaplasma | -0.01 | 4.33 | 0.00 | -0.01 | 0.00 | NA | 1.00 | 0.49 | 1.00 | 1.00 | 1.00 | 1.00 |
| f_Christensenellaceae | -0.05 | 1.74 | 0.15 | 0.02 | -0.26 | -0.36 | 1.00 | 0.78 | 1.00 | 1.00 | 1.00 | 1.00 |
| f_Actinomycetaceae | -0.02 | -0.77 | 0.05 | -0.07 | -0.63 | 1.54 | 1.00 | 1.00 | 1.00 | 1.00 | 1.00 | 1.00 |
| Brevibacterium | 0.02 | 0.18 | 0.05 | 0.00 | NA | 0.00 | 1.00 | 1.00 | 1.00 | 1.00 | 1.00 | 1.00 |
| Brachybacterium | -0.09 | 243.11 | 0.00 | 0.09 | 0.00 | -357.61 | 1.00 | 1.00 | 1.00 | 1.00 | 1.00 | 1.00 |
| f_Microbacteriaceae | NA | NA | NA | NA | NA | NA | 1.00 | 1.00 | 1.00 | 1.00 | 1.00 | 1.00 |
| Glutamicibacter | -0.02 | -1.27 | 0.06 | 0.01 | -0.92 | NA | 1.00 | 1.00 | 1.00 | 1.00 | 1.00 | 1.00 |
| Streptomyces | 0.03 | -0.65 | NA | 0.00 | NA | 0.00 | 1.00 | 1.00 | 1.00 | 1.00 | 1.00 | 1.00 |
| f_Atopobiaceae | -0.84 | 3353.58 | -152.38 | 0.00 | NA | 0.00 | 1.00 | 1.00 | 1.00 | 1.00 | 1.00 | 1.00 |
| f_Coriobacteriales_Incertae_Sedis | 0.07 | 0.67 | 0.06 | -0.09 | -0.16 | -0.21 | 1.00 | 1.00 | 1.00 | 1.00 | 1.00 | 1.00 |
| DNF00809 | 0.04 | -0.35 | 0.00 | -0.07 | 0.00 | NA | 1.00 | 1.00 | 1.00 | 1.00 | 1.00 | 1.00 |
| Enterorhabdus | 0.13 | 0.16 | 0.08 | -0.19 | -0.06 | 0.00 | 1.00 | 1.00 | 1.00 | 1.00 | 1.00 | 1.00 |
| f_Eggerthellaceae | 0.14 | 0.64 | 0.02 | -0.19 | -0.05 | -0.13 | 1.00 | 1.00 | 1.00 | 1.00 | 1.00 | 1.00 |
| f_Marinifilaceae | -0.01 | -0.11 | 0.04 | 0.03 | 0.09 | 0.10 | 1.00 | 1.00 | 1.00 | 1.00 | 1.00 | 1.00 |
| Odoribacter | -0.05 | 0.46 | 0.06 | 0.05 | -0.36 | -0.47 | 1.00 | 1.00 | 1.00 | 1.00 | 1.00 | 1.00 |
| f_Muribaculaceae | -0.04 | 0.19 | 0.09 | 0.05 | -0.12 | -0.16 | 1.00 | 1.00 | 1.00 | 1.00 | 1.00 | 1.00 |
| Muribaculaceae | 0.00 | 0.00 | 0.21 | 0.00 | 0.00 | -0.01 | 1.00 | 1.00 | 1.00 | 1.00 | 1.00 | 1.00 |
| Alistipes | 0.03 | 0.21 | 0.00 | 0.01 | 0.05 | 0.01 | 1.00 | 1.00 | 1.00 | 1.00 | 1.00 | 1.00 |
| f_Rikenellaceae | 0.07 | 0.09 | -0.01 | -0.08 | -0.40 | -0.19 | 1.00 | 1.00 | 1.00 | 1.00 | 1.00 | 1.00 |
| Rikenella | -0.05 | -0.33 | 0.06 | 0.05 | 1.51 | 0.71 | 1.00 | 1.00 | 1.00 | 1.00 | 0.81 | 1.00 |
| Rs-E47_termite_group | 0.12 | 0.17 | -0.02 | -0.07 | 0.37 | -0.24 | 1.00 | 1.00 | 1.00 | 1.00 | 1.00 | 1.00 |
| c_Bacteroidia | 0.02 | -0.96 | -0.01 | -0.04 | 2.48 | 1.80 | 1.00 | 1.00 | 1.00 | 1.00 | 0.14 | 0.88 |
| Gastranaerophilales | -0.01 | -0.57 | 0.04 | 0.05 | 0.01 | -0.11 | 1.00 | 1.00 | 1.00 | 1.00 | 1.00 | 1.00 |
| Mucispirillum | -0.02 | -1.33 | -0.19 | 0.01 | 4.24 | -0.69 | 1.00 | 1.00 | 1.00 | 1.00 | 0.08 | 1.00 |
| Bilophila | 0.02 | -0.19 | -0.02 | -0.04 | 1.42 | 0.75 | 1.00 | 1.00 | 1.00 | 1.00 | 0.88 | 1.00 |
| Desulfovibrio | 0.22 | 0.28 | -0.01 | -0.26 | -0.06 | -0.08 | 1.00 | 1.00 | 1.00 | 1.00 | 1.00 | 1.00 |
| f_Desulfovibrionaceae | 0.07 | -0.59 | 0.00 | -0.08 | 0.10 | 0.16 | 1.00 | 1.00 | 1.00 | 1.00 | 1.00 | 1.00 |
| c_Bacilli | NA | NA | NA | NA | NA | NA | 1.00 | 1.00 | 1.00 | 1.00 | 1.00 | 1.00 |
| Allobaculum | 0.02 | -0.01 | -0.01 | -0.08 | 0.18 | 0.24 | 1.00 | 1.00 | 1.00 | 1.00 | 1.00 | 1.00 |
| f_Erysipelotrichaceae | 0.01 | 0.05 | 0.02 | 0.01 | 98.97 | NA | 1.00 | 1.00 | 1.00 | 1.00 | 0.00 | 1.00 |
| Aerococcus | 1.15 | -129.40 | -1.12 | 0.00 | NA | 0.00 | 1.00 | 1.00 | 1.00 | 1.00 | 1.00 | 1.00 |
| f_Lactobacillaceae | 0.08 | -191.04 | -0.10 | 0.00 | 292.64 | 0.00 | 1.00 | 1.00 | 1.00 | 1.00 | 1.00 | 1.00 |
| Lactobacillus | 0.07 | -0.29 | 0.17 | -0.15 | 0.03 | 0.28 | 1.00 | 1.00 | 1.00 | 1.00 | 1.00 | 1.00 |
| o_Lactobacillales | -0.01 | -2.52 | 0.06 | 0.18 | NA | NA | 1.00 | 1.00 | 1.00 | 1.00 | 1.00 | 1.00 |
| Lactococcus | -0.02 | 3.13 | 0.00 | 0.01 | 0.00 | NA | 1.00 | 1.00 | 1.00 | 1.00 | 1.00 | 1.00 |
| Streptococcus | 0.09 | 0.27 | -0.01 | -0.11 | -0.12 | -0.18 | 1.00 | 1.00 | 1.00 | 1.00 | 1.00 | 1.00 |
| Mycoplasma | -0.03 | 0.45 | 0.01 | 0.00 | 0.85 | -0.40 | 1.00 | 1.00 | 1.00 | 1.00 | 1.00 | 1.00 |
| RF39 | 0.05 | 0.03 | -0.01 | -0.06 | 0.43 | -0.20 | 1.00 | 1.00 | 1.00 | 1.00 | 1.00 | 1.00 |
| c_Clostridia | 0.05 | 0.49 | 0.01 | -0.09 | -2.84 | -0.84 | 1.00 | 1.00 | 1.00 | 1.00 | 0.08 | 1.00 |
| Christensenella | 0.05 | -0.60 | 0.00 | -0.04 | 0.00 | NA | 1.00 | 1.00 | 1.00 | 1.00 | 1.00 | 1.00 |
| Christensenellaceae_R-7_group | NA | NA | NA | NA | NA | NA | 1.00 | 1.00 | 1.00 | 1.00 | 1.00 | 1.00 |
| Clostridia_UCG-014 | 0.08 | -0.15 | -0.01 | -0.07 | 0.23 | 0.18 | 1.00 | 1.00 | 1.00 | 1.00 | 1.00 | 1.00 |
| Clostridia_vadinBB60_group | 0.01 | -0.25 | 0.00 | 0.08 | 0.12 | -0.05 | 1.00 | 1.00 | 1.00 | 1.00 | 1.00 | 1.00 |
| Candidatus_Arthromitus | 0.00 | 0.69 | 0.05 | -0.02 | -0.99 | -0.29 | 1.00 | 1.00 | 1.00 | 1.00 | 1.00 | 1.00 |
| Anaerofustis | -0.01 | 0.72 | NA | 0.00 | NA | 0.00 | 1.00 | 1.00 | 1.00 | 1.00 | 1.00 | 1.00 |
| [Eubacterium]_ventriosum_group | -0.10 | -2.11 | 0.05 | 0.10 | NA | NA | 1.00 | 1.00 | 1.00 | 1.00 | 1.00 | 1.00 |
| [Eubacterium]_xylanophilum_group | 0.00 | 0.09 | 0.02 | -0.02 | -0.06 | -0.03 | 1.00 | 1.00 | 1.00 | 1.00 | 1.00 | 1.00 |
| A2 | 0.02 | -0.89 | 0.03 | -0.01 | 1.03 | 1.28 | 1.00 | 1.00 | 1.00 | 1.00 | 1.00 | 1.00 |
| Acetatifactor | 0.07 | 0.86 | -0.07 | -0.06 | -0.73 | -0.93 | 1.00 | 1.00 | 1.00 | 1.00 | 1.00 | 1.00 |
| Anaerostipes | 0.01 | 0.14 | 0.04 | 0.01 | -6.95 | -2.01 | 1.00 | 1.00 | 1.00 | 1.00 | 0.00 | 0.85 |
| ASF356 | 0.06 | 0.27 | -0.06 | -0.04 | NA | -0.60 | 1.00 | 1.00 | 1.00 | 1.00 | 1.00 | 1.00 |
| Blautia | 0.04 | -0.10 | 0.02 | -0.03 | -0.38 | 0.44 | 1.00 | 1.00 | 1.00 | 1.00 | 1.00 | 1.00 |
| Coprococcus | -0.01 | 0.33 | 0.00 | -0.03 | 0.00 | NA | 1.00 | 1.00 | 1.00 | 1.00 | 1.00 | 1.00 |
| Dorea | -0.03 | -0.58 | 0.05 | 0.07 | 2.34 | 0.80 | 1.00 | 1.00 | 1.00 | 1.00 | 0.21 | 1.00 |
| f_Lachnospiraceae | 0.22 | -0.14 | -0.20 | -0.17 | -0.02 | -0.03 | 1.00 | 1.00 | 1.00 | 1.00 | 1.00 | 1.00 |
| Lachnoclostridium | 0.00 | 1.31 | 0.00 | 0.00 | -0.98 | -1.43 | 1.00 | 1.00 | 1.00 | 1.00 | 1.00 | 1.00 |
| Lachnospiraceae_FCS020_group | -0.01 | -0.07 | 0.00 | 0.01 | 0.00 | 0.31 | 1.00 | 1.00 | 1.00 | 1.00 | 1.00 | 1.00 |
| Lachnospiraceae_NC2004_group | 0.00 | 0.10 | 0.08 | 0.00 | NA | -0.68 | 1.00 | 1.00 | 1.00 | 1.00 | 1.00 | 1.00 |
| Lachnospiraceae_NK4A136_group | 0.04 | 0.49 | 0.01 | -0.01 | -0.07 | -0.12 | 1.00 | 1.00 | 1.00 | 1.00 | 1.00 | 1.00 |
| Lachnospiraceae_NK4B4_group | 0.03 | -0.48 | 0.00 | 0.00 | 0.00 | NA | 1.00 | 1.00 | 1.00 | 1.00 | 1.00 | 1.00 |
| Lachnospiraceae_UCG-001 | -0.01 | 0.30 | 0.05 | 0.03 | -1.54 | -0.59 | 1.00 | 1.00 | 1.00 | 1.00 | 0.81 | 1.00 |
| Lachnospiraceae_UCG-008 | -0.06 | 202.88 | 0.00 | NA | 0.00 | NA | 1.00 | 1.00 | 1.00 | 1.00 | 1.00 | 1.00 |
| Lachnospiraceae_UCG-010 | 0.02 | 0.38 | 0.00 | -0.01 | 0.00 | NA | 1.00 | 1.00 | 1.00 | 1.00 | 1.00 | 1.00 |
| Roseburia | 0.11 | 0.12 | -0.10 | -0.11 | -0.33 | 0.27 | 1.00 | 1.00 | 1.00 | 1.00 | 1.00 | 1.00 |
| Tyzzerella | 0.08 | 0.14 | -0.07 | -0.08 | 0.35 | 0.69 | 1.00 | 1.00 | 1.00 | 1.00 | 1.00 | 1.00 |
| Monoglobus | 0.08 | -0.12 | -0.05 | -0.09 | 0.47 | 0.29 | 1.00 | 1.00 | 1.00 | 1.00 | 1.00 | 1.00 |
| [Eubacterium]_coprostanoligenes_group | 0.03 | -0.13 | 0.01 | -0.02 | 0.04 | 0.38 | 1.00 | 1.00 | 1.00 | 1.00 | 1.00 | 1.00 |
| Butyricicoccus | 0.03 | 0.83 | -0.13 | -0.05 | NA | -2.33 | 1.00 | 1.00 | 1.00 | 1.00 | 1.00 | 0.85 |
| f_Butyricicoccaceae | -0.04 | 0.39 | 0.10 | 0.02 | -2.83 | -0.87 | 1.00 | 1.00 | 1.00 | 1.00 | 0.17 | 1.00 |
| UCG-009 | -0.04 | -0.42 | 0.09 | 0.04 | -3.19 | 0.78 | 1.00 | 1.00 | 1.00 | 1.00 | 0.07 | 1.00 |
| Colidextribacter | 0.08 | 0.14 | -0.08 | -0.06 | -0.06 | 0.23 | 1.00 | 1.00 | 1.00 | 1.00 | 1.00 | 1.00 |
| f_Oscillospiraceae | 0.26 | -0.60 | -0.28 | -0.17 | 0.02 | 0.01 | 1.00 | 1.00 | 1.00 | 1.00 | 1.00 | 1.00 |
| Intestinimonas | 0.04 | -0.18 | 0.00 | -0.05 | 0.00 | NA | 1.00 | 1.00 | 1.00 | 1.00 | 1.00 | 1.00 |
| Oscillibacter | 0.07 | -0.10 | -0.07 | -0.08 | 0.09 | 0.19 | 1.00 | 1.00 | 1.00 | 1.00 | 1.00 | 1.00 |
| Oscillospira | -0.02 | -0.04 | 0.00 | 0.02 | 0.00 | NA | 1.00 | 1.00 | 1.00 | 1.00 | 1.00 | 1.00 |
| Papillibacter | 0.02 | 0.12 | 0.00 | -0.04 | 0.00 | NA | 1.00 | 1.00 | 1.00 | 1.00 | 1.00 | 1.00 |
| UCG-005 | 0.03 | -0.25 | 0.00 | -0.03 | 1.29 | 0.67 | 1.00 | 1.00 | 1.00 | 1.00 | 1.00 | 1.00 |
| V9D2013_group | -0.02 | -0.19 | 0.00 | 0.03 | 0.00 | NA | 1.00 | 1.00 | 1.00 | 1.00 | 1.00 | 1.00 |
| [Eubacterium]_siraeum_group | 0.09 | -0.33 | -0.04 | -0.12 | 0.11 | 0.22 | 1.00 | 1.00 | 1.00 | 1.00 | 1.00 | 1.00 |
| Anaerofilum | NA | NA | NA | NA | NA | NA | 1.00 | 1.00 | 1.00 | 1.00 | 1.00 | 1.00 |
| Anaerotruncus | -0.04 | -0.07 | 0.00 | 0.04 | 0.00 | NA | 1.00 | 1.00 | 1.00 | 1.00 | 1.00 | 1.00 |
| Candidatus_Soleaferrea | -1.03 | 5650.37 | -233.96 | 0.00 | NA | 0.00 | 1.00 | 1.00 | 1.00 | 1.00 | 1.00 | 1.00 |
| Caproiciproducens | -0.01 | -0.22 | 0.09 | 0.00 | NA | 0.00 | 1.00 | 1.00 | 1.00 | 1.00 | 1.00 | 1.00 |
| f_Ruminococcaceae | 0.13 | 0.34 | -0.07 | -0.08 | -0.05 | -0.10 | 1.00 | 1.00 | 1.00 | 1.00 | 1.00 | 1.00 |
| Harryflintia | 0.44 | -0.09 | -0.43 | -0.13 | 9.09 | -1.17 | 1.00 | 1.00 | 1.00 | 1.00 | 0.00 | 1.00 |
| Incertae_Sedis | 0.02 | -0.43 | -0.03 | -0.02 | 0.07 | 0.13 | 1.00 | 1.00 | 1.00 | 1.00 | 1.00 | 1.00 |
| Paludicola | -0.55 | 12.11 | 0.00 | -0.42 | 0.00 | NA | 1.00 | 1.00 | 1.00 | 1.00 | 1.00 | 1.00 |
| Ruminococcaceae | 0.00 | -0.54 | 0.04 | -0.01 | 1.78 | 0.49 | 1.00 | 1.00 | 1.00 | 1.00 | 0.67 | 1.00 |
| Ruminococcus | 0.18 | -0.20 | -0.16 | -0.15 | 0.19 | 0.18 | 1.00 | 1.00 | 1.00 | 1.00 | 1.00 | 1.00 |
| UBA1819 | 0.04 | -0.66 | -0.04 | -0.05 | 1.40 | 1.10 | 1.00 | 1.00 | 1.00 | 1.00 | 0.88 | 1.00 |
| UCG-010 | -0.04 | 0.70 | 0.05 | 0.05 | -0.43 | -0.76 | 1.00 | 1.00 | 1.00 | 1.00 | 1.00 | 1.00 |
| f_Peptococcaceae | 0.02 | 0.00 | 0.02 | 0.00 | 0.05 | 0.17 | 1.00 | 1.00 | 1.00 | 1.00 | 1.00 | 1.00 |
| Peptococcus | 0.01 | -0.16 | -0.02 | -0.03 | NA | 0.36 | 1.00 | 1.00 | 1.00 | 1.00 | 1.00 | 1.00 |
| [Eubacterium]_brachy_group | 0.08 | 0.02 | -0.03 | -0.10 | 0.07 | -0.09 | 1.00 | 1.00 | 1.00 | 1.00 | 1.00 | 1.00 |
| [Eubacterium]_nodatum_group | 0.02 | 0.80 | 0.00 | -0.11 | 0.00 | NA | 1.00 | 1.00 | 1.00 | 1.00 | 1.00 | 1.00 |
| f_Anaerovoracaceae | NA | NA | NA | NA | NA | NA | 1.00 | 1.00 | 1.00 | 1.00 | 1.00 | 1.00 |
| Family_XIII_AD3011_group | 0.07 | -0.44 | -0.04 | -0.07 | 0.05 | -0.04 | 1.00 | 1.00 | 1.00 | 1.00 | 1.00 | 1.00 |
| Family_XIII_UCG-001 | 0.01 | -0.60 | 0.01 | -0.01 | 2.20 | 1.69 | 1.00 | 1.00 | 1.00 | 1.00 | 0.24 | 1.00 |
| o_Peptostreptococcales-Tissierellales | -0.11 | 465.24 | NA | 0.00 | NA | 0.00 | 1.00 | 1.00 | 1.00 | 1.00 | 1.00 | 1.00 |
| Quinella | 0.00 | 0.36 | 0.02 | NA | NA | NA | 1.00 | 1.00 | 1.00 | 1.00 | 1.00 | 1.00 |
| Veillonella | NA | NA | NA | NA | NA | NA | 1.00 | 1.00 | 1.00 | 1.00 | 1.00 | 1.00 |
| p_Firmicutes | NA | NA | NA | NA | NA | NA | 1.00 | 1.00 | 1.00 | 1.00 | 1.00 | 1.00 |
| Candidatus_Saccharimonas | 0.05 | -0.40 | 0.01 | -0.07 | 0.10 | 0.08 | 1.00 | 1.00 | 1.00 | 1.00 | 1.00 | 1.00 |
| f_Paracaedibacteraceae | 0.11 | -0.81 | -0.04 | -0.11 | 1.50 | 1.62 | 1.00 | 1.00 | 1.00 | 1.00 | 0.82 | 1.00 |
| o_Rhodospirillales | 0.03 | -0.70 | 0.00 | -0.05 | 3.30 | 2.28 | 1.00 | 1.00 | 1.00 | 1.00 | 0.03 | 0.43 |
| o_Burkholderiales | 0.04 | -0.12 | 0.00 | -0.07 | -0.39 | 0.11 | 1.00 | 1.00 | 1.00 | 1.00 | 1.00 | 1.00 |
| f_Oxalobacteraceae | 0.10 | 0.48 | -0.07 | -0.10 | 1.69 | 0.18 | 1.00 | 1.00 | 1.00 | 1.00 | 0.67 | 1.00 |
| c_Gammaproteobacteria | 0.05 | 0.93 | 0.02 | -0.09 | -0.40 | -0.28 | 1.00 | 1.00 | 1.00 | 1.00 | 1.00 | 1.00 |
| Rickettsiella | NA | NA | NA | NA | NA | NA | 1.00 | 1.00 | 1.00 | 1.00 | 1.00 | 1.00 |
| Hafnia-Obesumbacterium | 0.11 | -11.25 | -0.02 | 0.00 | NA | 0.00 | 1.00 | 1.00 | 1.00 | 1.00 | 1.00 | 1.00 |
| f_Pasteurellaceae | -0.04 | 1.40 | 0.30 | 0.06 | -4.57 | NA | 1.00 | 1.00 | 1.00 | 1.00 | 0.07 | 1.00 |
| Brachyspira | -0.02 | 0.32 | 0.03 | 0.01 | NA | NA | 1.00 | 1.00 | 1.00 | 1.00 | 1.00 | 1.00 |
| Treponema | -0.07 | 0.84 | 0.14 | -0.01 | -1.18 | -0.65 | 1.00 | 1.00 | 1.00 | 1.00 | 1.00 | 1.00 |
| f_Puniceicoccaceae | -0.04 | -3.38 | 0.12 | 0.00 | NA | 0.00 | 1.00 | 1.00 | 1.00 | 1.00 | 1.00 | 1.00 |
| Citricoccus | 0.04 | -0.39 | NA | 0.00 | NA | 0.00 | 1.00 | 1.00 | 1.00 | 1.00 | 1.00 | 1.00 |
| o_Coriobacteriales | 0.06 | 1.55 | 0.05 | -0.10 | -0.25 | -0.42 | 1.00 | 1.00 | 1.00 | 1.00 | 1.00 | 1.00 |
| o_Bacteroidales | -0.49 | 5.07 | 0.00 | 0.50 | 0.00 | NA | 1.00 | 1.00 | 1.00 | 1.00 | 1.00 | 1.00 |
| Helicobacter | 0.08 | 0.02 | -0.09 | -0.09 | -0.06 | 0.15 | 1.00 | 1.00 | 1.00 | 1.00 | 1.00 | 1.00 |
| Anaeroplasma | 0.05 | -0.82 | -0.03 | -0.02 | 0.32 | 0.16 | 1.00 | 1.00 | 1.00 | 1.00 | 1.00 | 1.00 |
| Carnobacterium | 0.05 | -125.35 | 5.06 | 0.00 | NA | 0.00 | 1.00 | 1.00 | 1.00 | 1.00 | 1.00 | 1.00 |
| Christensenellaceae | 0.00 | -0.44 | 0.07 | -0.01 | 0.54 | 0.57 | 1.00 | 1.00 | 1.00 | 1.00 | 1.00 | 1.00 |
| Ruminiclostridium | -0.04 | 8.19 | 0.00 | -0.05 | 0.00 | NA | 1.00 | 1.00 | 1.00 | 1.00 | 1.00 | 1.00 |
| Tuzzerella | 0.01 | -0.24 | 0.00 | NA | 0.00 | NA | 1.00 | 1.00 | 1.00 | 1.00 | 1.00 | 1.00 |
| NK4A214_group | 0.04 | 0.31 | -0.06 | -0.08 | -0.26 | -0.60 | 1.00 | 1.00 | 1.00 | 1.00 | 1.00 | 1.00 |
| Acinetobacter | -25.81 | 264.38 | 27.18 | 26.81 | NA | NA | 1.00 | 1.00 | 1.00 | 1.00 | 1.00 | 1.00 |
